# Supplementary material for: Givinostat enhances antisense oligonucleotide efficacy in the mdx52 model of Duchenne muscular dystrophy
Source: Mol Ther Nucleic Acids. 2026 Aug 3;37(3):103047. doi: 10.1016/j.omtn.2026.103047 (PMC13521170; doi:10.1016/j.omtn.2026.103047)
Supplement: Supplementary file 1 [file mmc1.pdf]

OMTN, Volume *37*

Supplemental information

Givinostat enhances antisense oligonucleotide efficacy in the *mdx52* model of Duchenne muscular dystrophy

Xaysongkhame Phongsavanh, Cecile Gastaldi, Astrid Mottais, Mathilde Doisy, Olivier Le Coz, Sandra de Haan, Pietro Spitali, Luis Garcia, and Aurélie Goyenvalle

Supporting methods

Cell viability assay following HDACi treatment during early differentiation

To evaluate the cytotoxic effects of histone deacetylase inhibitors (HDACi) during early myogenic differentiation, cell viability was assessed using the MTT Cell Proliferation Assay Kit (VWR, OZBIMT01000) after 48 hours of treatment. Human and murine myoblasts were seeded in 6-well plates at a density of 300,000 cells per well in their respective proliferation media (see *Cell Culture, Differentiation, HDACi Treatment, and Oligonucleotide Transfection* for detailed compositions). Cells were maintained under standard incubation conditions (human cells: 37°C, 5% CO_2; murine cells: 33°C, 10% CO_2). Twenty-four hours after seeding, cells were treated with HDAC inhibitors at various concentrations: Givinostat (Givi) and trichostatin A (TSA) at 10 nM, 50 nM, 150 nM, and 300 nM; valproic acid (VPA) at 0.5 mM, 1 mM, 3 mM, and 10 mM. Differentiation was induced simultaneously by replacing the proliferation medium with the appropriate differentiation medium (see *Cell Culture* section for formulations), and cells were incubated under differentiation conditions (37°C, 5% CO_2) for 48 hours. Following treatment, the MTT assay was performed according to the manufacturer's instructions. Cells were incubated with MTT reagent for the recommended duration (typically 2–4 hours), after which the resulting formazan crystals were solubilized using the provided solubilization buffer. Absorbance was measured at 570 nm using a microplate reader. Cell viability was expressed as a percentage relative to untreated controls. All conditions were tested in triplicate (n = 3) and appropriate vehicle controls were included for each HDACi treatment.

ASO quantification by fluorescent hybridization assay

Tissues were homogenized using the Precellys 24 (Bertin Instruments, France) in lysis buffer (100 mmol/l Tris–HCl, pH 8.5, 200 mmol/l NaCl, 5 mmol/l EDTA, 0.2% sodium dodecyl sulfate) containing 2 mg/ml of proteinase K (Invitrogen), at a final tissue concentration of 50 mg per ml of buffer. The homogenates were incubated overnight at 55°C in a hybridization oven. After a 15-minute

centrifugation at 7,000 g (Sorval ST 8R centrifuge, 75005719 rotor), the supernatant was collected for further analysis. ASO quantification was performed using a hybridization assay with a molecular beacon probe, as previously described [39]. The concentration of tcDNA in tissues was determined by comparing the fluorescence signals to a standard curve generated from known amounts of tcDNA dissolved in tissue lysates from PBS-injected animals.

Table S1. Quantification of dystrophin restoration across muscles following 4- and 12-week treatment protocols.

Tables showing the values for quantification of dystrophin restoration for each muscle following the 4-week (n = 4 per group) and 12-week (n = 6 per group) treatment protocols (mean ± SEM, expressed as a percentage of wild-type dystrophin). Fold-change values between treatments are shown for the 12-week protocol. An average fold change of ~1.3-fold is shown and corresponds to the average fold change across all muscles, indicating that the combined Givi + ASO treatment increases dystrophin restoration compared with ASO monotherapy. TA: tibialis anterior, GAS: gastrocnemius, Q: quadriceps, TRI: triceps, DIA: diaphragm, heart.

4-weeks	TA	TRI	DIA	Heart	Average
**ASO**	**0.27** ± 0.07 %	**0.07** ± 0.04 %	**0.00** ± 0.00 %	**2.14** ± 0.8 %	**0.62** ± 0.22
**Givi+ASO**	**1.42**	**0.80**	**0.54**	**3.17**	**1.48**

12-weeks	TA	GAS	Q	TRI	DIA	Heart	Average
ASO	**2.17** ± 0.12 %	**2.7** ± 0.76 %	**3.44** ± 0.42 %	**2.10** ± 0.48 %	**2.25** ± 0.22 %	**5.21** ± 1.59 %	**2.99** ± 0.60
Givi+ASO	**2.09** ± 0.21 %	**4.22** ± 0.89 %	**3.77** ± 0.13 %	**2.91** ± 0.26 %	**3.48** ± 0.60 %	**7.26** ± 1.48 %	**3.86** ± 0.60
Fold change	**0.96**	**1.53**	**1.09**	**1.10**	**1.55**	**1.39**	**1.29**

Table S2. List of genes with their associated primers and TaqMan probes

Targets	Forward (5' →3')	Reverse (5'→3')	Probes
hex4-5 (Hs.PT.58.27519607)	TGCCCTGAACAATGTCAACA	CTGCCAGTGGAGGATTATATTCC	/56-FAM/CAAACCAAG/ZEN/AGTCAGTTTATGATTTCCATCTACGA/3IABkFQ/
hex23-24 (Hs.PT.5821042878)	GAAGAAGCTCTCCTCCCA	CTTCAGAAAAACATCAACTTCAGC	/56-FAM/CTCCGAAAA/ZEN/ATTCAGAATCACATACAAACCCTG/3IABkFQ/
hex49-50 (hDMD49-50 set 3)	GTTTACCGCCTTCCACTCA	TTTGTCTAAAGGGCAGCATTTG	/56-FAM/CTTCCTCTT/ZEN/CACTGGCTGAGTGGC/3IABkFQ/
hex53-54 (Hs.PT.58.24504618)	GTCCCTATACAGTAGATGCAATCC	ATAATCCCGGAGAAGTTTCAGG	/56-FAM/CACTGGCGG/ZEN/AGGTCTTTGGC/3IABkFQ/
hex58-59 (Hs.PT.58.21052090)	CTCTTGAGACTGTACGAATATTTCTG	GAGTGACATTCTGGGCTCTC	/56-FAM/TCTCTAGTC/ZEN/CTTCCAAAGGCTGCTCT/3IABkFQ/
hEx65-66 (Hs.PT.58.1307753)	CTCTGCGTGGATATGTGTCTG	CTGTACTTGTCTTCCAAATGTGC	/56-FAM/TGGCTGCTG/ZEN/AATGTTTATGATACGGGA/3IABkFQ/
hGAPDH (Hs.PT.39a22214836)	ACATCGCTCAGACACCATG	TGTAGTTGAGGTCAATGAAGGG	/56-FAM/AAGGTCGGA /ZEN/GTCAACGGATTTGGTC/3IABkFQ/
mex4-5 (Mm.PT.58.41636025)	ATCCACTATGTCAGTGCTTCC	AGGGATCTACAAGAGTTCATGC	/56-FAM/AACAATGTC /ZEN/AACAAGGCACTGCGG /3IABkFQ/
mex23-24 (Mm.PT.58.434322707)	CAGGCCATTCCTCTTTCAGG	GAAACTTTCCTCCCAGTTGGT	/56-FAM/TCAACTTCA /ZEN/GCCATCCATTTCTGTAAGGT/3IABkFQ/
mex49-50 (Mm.PT.58.6233636)	GATGTGGAAAGGCTTTTGTCG	CTCCCACTCAGACCTCAGAT	/56-FAM/AACCAAGCA /ZEN/CTCAGCCAGTGAAGA/3IABkFQ/
mex53-54 (Mm.PT. 58.41709800)	AGAAGGTCCTCACACAGTAGA	CAGCAGAATAGTCCCGAAGAAG	/56-FAM/CGGCAGATA/ZEN/AGTGTAGACGTGGCA/3IABkFQ/
mex58-59 (Mm.PT.58.43613256)	TTCTGACAGAGCAGCCTTTG	GAGCCGAGTGACATTCTGAG	/56-FAM/AGCCCAGAG /ZEN/AACTGCCTCCTG/3IABkFQ/
mex65-66 (Mm.PT.58.42993407)	GTGTCTCAACTGGCTTCTCAA	CTTTACACAGAAATGATGCCA	/56-FAM/TTATGATAC/ZEN/GGGACGAACAGGGAGGA/3IABkFQ/

mGAPDH (Mm.PT.39a.1)	AATGGTGAAGGTCGGTGTG	GTGGAGTCATACTGGAACATGTA	/56-FAM/TGCAAATGG/ZEN/CAGCCCTGGTG/3IABkFQ/
mex50-51 (MM.PT.58.41685801)	CAAAGCAGCCTGACCGT	TGACAGTTTCCTTAGTAACCACAG	/56-FAM/TGGACTGAG/ZEN/CACTACTGGAGCCT/3IABkFQ/
mexon 50-53	CTTCCAGCCATTGTGTTGAATC	GCACTACTGGAGCCTTTGAA	/56-FAM/ACAGCTGCA/ZEN/GAACAGGAGACAACA/3IABkFQ/
hex49F	AAACTGAAATAGCAGTTCAAGC		
hex53R		TTGCCTCCGGTTCTGAAGG	
mex50F	AGGAAGTTAGAAGATCTGAGG		
mex55R		GGAACTGCTGCAGTAATCTATGA	
CTGF	TTGACAGGCTTGGCGATT	GTTACCAATGACAATACCTTCTGC	
Col1a1	CATTGTGTATGCAGCTGACTTC	CGCAAAGAGTCTACATGTCTAGG	
Mfap4	CAGCTTGTAGTCGCTCCAG	CGTCTTCTGCGACATGACAA	
PGC-1α	TCGCTCAATAGTCTTGTTCTCAA	AGAAGTCCCATACACAACCG	
Myh1	GGACAAACTGCAATCAAAGGTC	CTGGATCTTGCGGAATTTGGG	
Apol9A	GCAGCTACTGTGACTGATGT	GGCAATCCTATCCACACTTGT	
Ddx60	CTCCTTGTTATGATCCGATGTCC	ATGGTACTGTCTACTTGCTTCC	
Isg15	CACAGTGATCAAGCATTTGCG	CCCCCATCATCTTTTATAACCAAC	
Oasl2	GCTTCACAGAGTTACAGAGACA	GCACCTCTTCCATATTTACGTCT	
MyoG	GACCGAACTCCAGTGCATT	CTTGCTCAGCTCCCTCAAC	

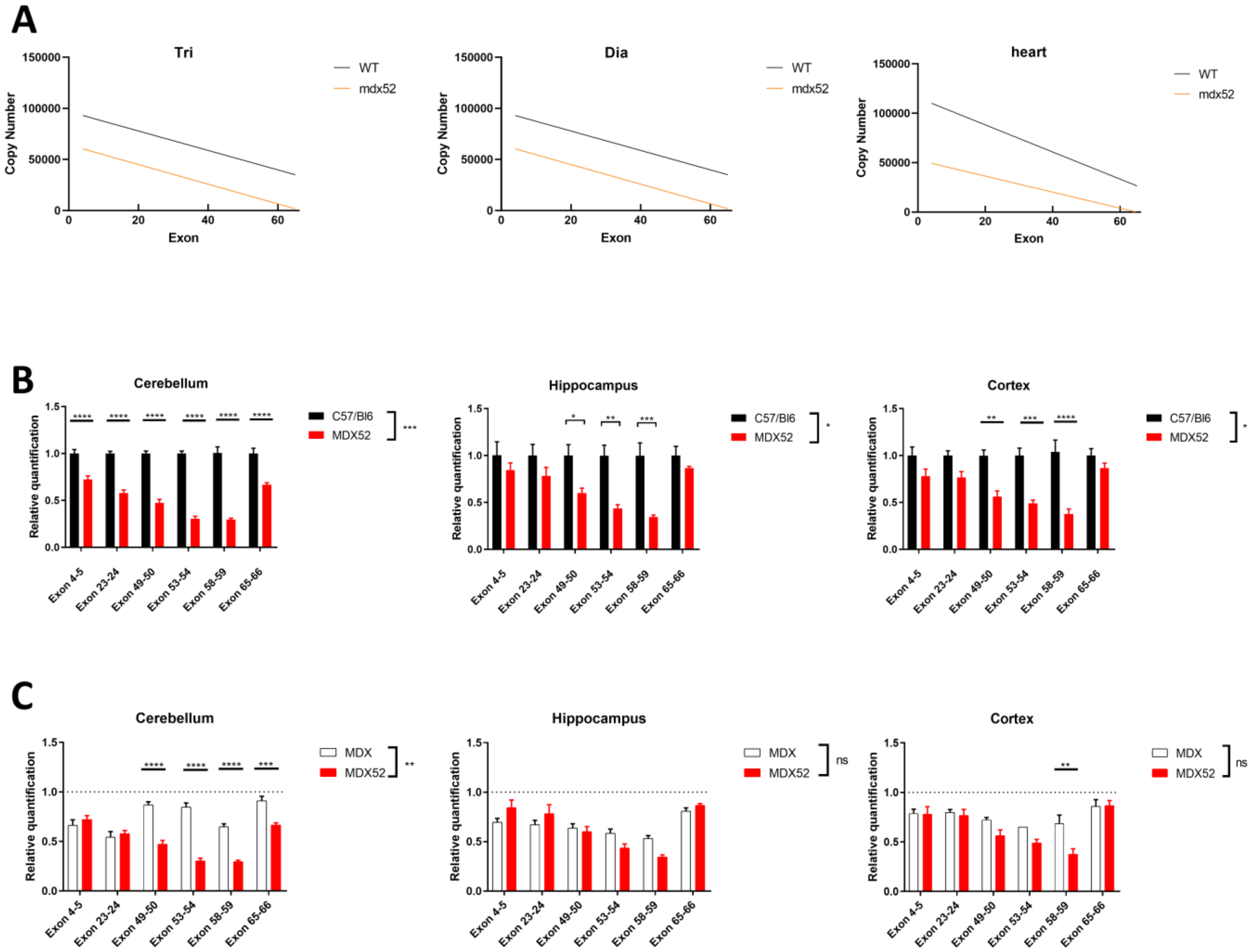

Figure S1. Additional transcriptomic and viability analyses. (A) Comparison of *Dmd* 5′–3′ transcript imbalance between *C57BL/6 WT* and *mdx52* mice. Linear regression analysis of 5′–3′ *Dmd* transcript imbalance was performed across different exon–exon junctions in triceps, diaphragm, and heart tissues from wild-type (*WT*; black) and *mdx52* (orange) mice (n = 3/group). **(B)** In vivo characterization of *Dmd* transcript imbalance in the brain by qPCR. Relative transcript levels across exon–exon junctions of the *Dmd* gene were measured in the cerebellum, hippocampus, and cortex of *mdx52* and *C57BL/6 WT* mice (n = 3/group), normalized to GAPDH and presented relative to WT. Significant imbalances: cerebellum (p = 0.0001), hippocampus (p = 0.0497), cortex (p = 0.0188); two-way ANOVA. **(C)** Comparison of *Dmd* transcript imbalance in *mdx* vs *mdx52* brain tissues. Transcript levels were quantified in cerebellum, hippocampus, and cortex (n = 3/group), normalized to *GAPDH* and WT. A significant difference was found in the cerebellum (p = 0.0083); hippocampus and cortex were not significantly different (p = 0.8773, p = 0.1538); two-way ANOVA.

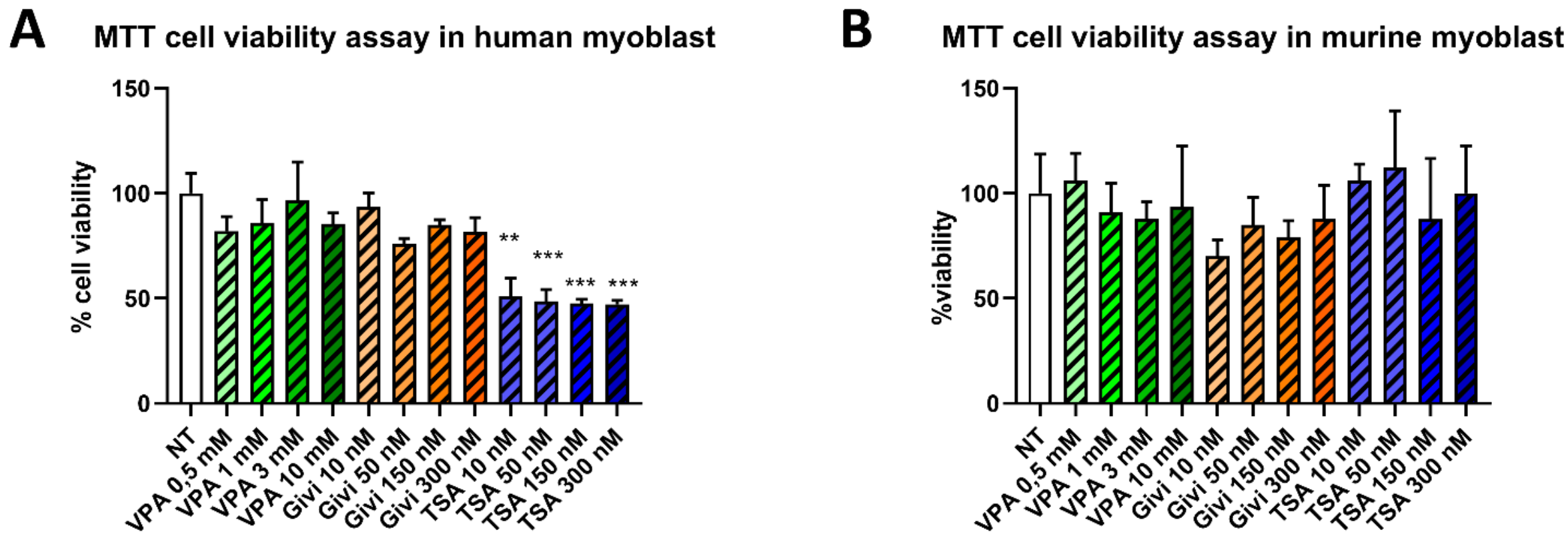

Figure S2. Cell Viability of Human and Murine Myoblasts Following HDACi Treatment. (A) Human and **(B)** murine myoblasts were plated at 300,000 cells/well, treated with 24h of Valproic Acid (VPA), Givinostat (Givi), or Trichostatin A (TSA), and differentiated for 2 days (n = 3/group). Cell viability was assessed using MTT assay. Left: viability in human myoblasts. Right: murine myoblasts. White = PBS; green, orange, blue = VPA, Givi, TSA; hatched = ASO. One-way ANOVA: Significant differences between PBS and human myotubes treated with TSA 10 nM, 50 nM, 150 nM and 300 nM (p = 0.0011, p = 0.0006, p = 0.0005 and p = 0.0004 respectively).

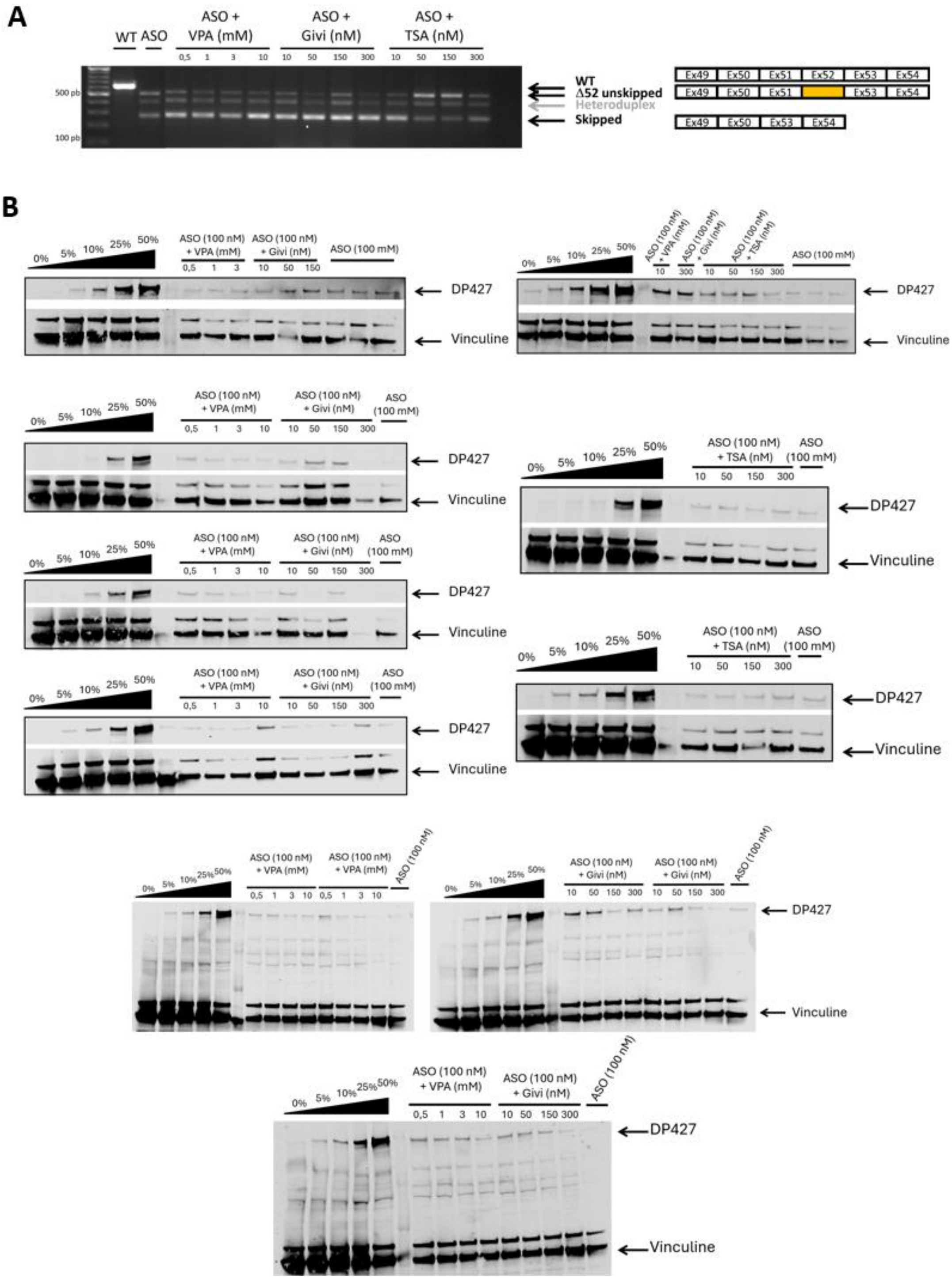

Figure S3. Effect of combined therapy in human cells. **(A)** Example of RT-PCR gel electrophoresis showing exon 51 skipping after treatment with ASO and increasing HDACi concentrations. Amplification spans exons 49 to 54. The untreated wild-type control shows the top band. In treated *KM571Δ52* samples, three bands are visible: unskipped Δ52 (top), a likely heteroduplex (middle) in gray, and skipped (bottom). **(B)** Western blot showing vinculin for normalization and dystrophin (DP427) restoration following ASO treatment alone or in combination with HDAC inhibitors. Three HDACi were tested at multiple doses: VPA (n=7 replicates for each dose 0.5, 1, 3, and 10 mM), Givinostat (n=7 replicates for each dose 10, 50, 150, and 300 nM), and TSA (n=3 for each dose 10, 50, 150, and 300 nM). A standard curve was generated using mixtures of WT and *mdx52*-PBS protein lysates containing 0–50% dystrophin.

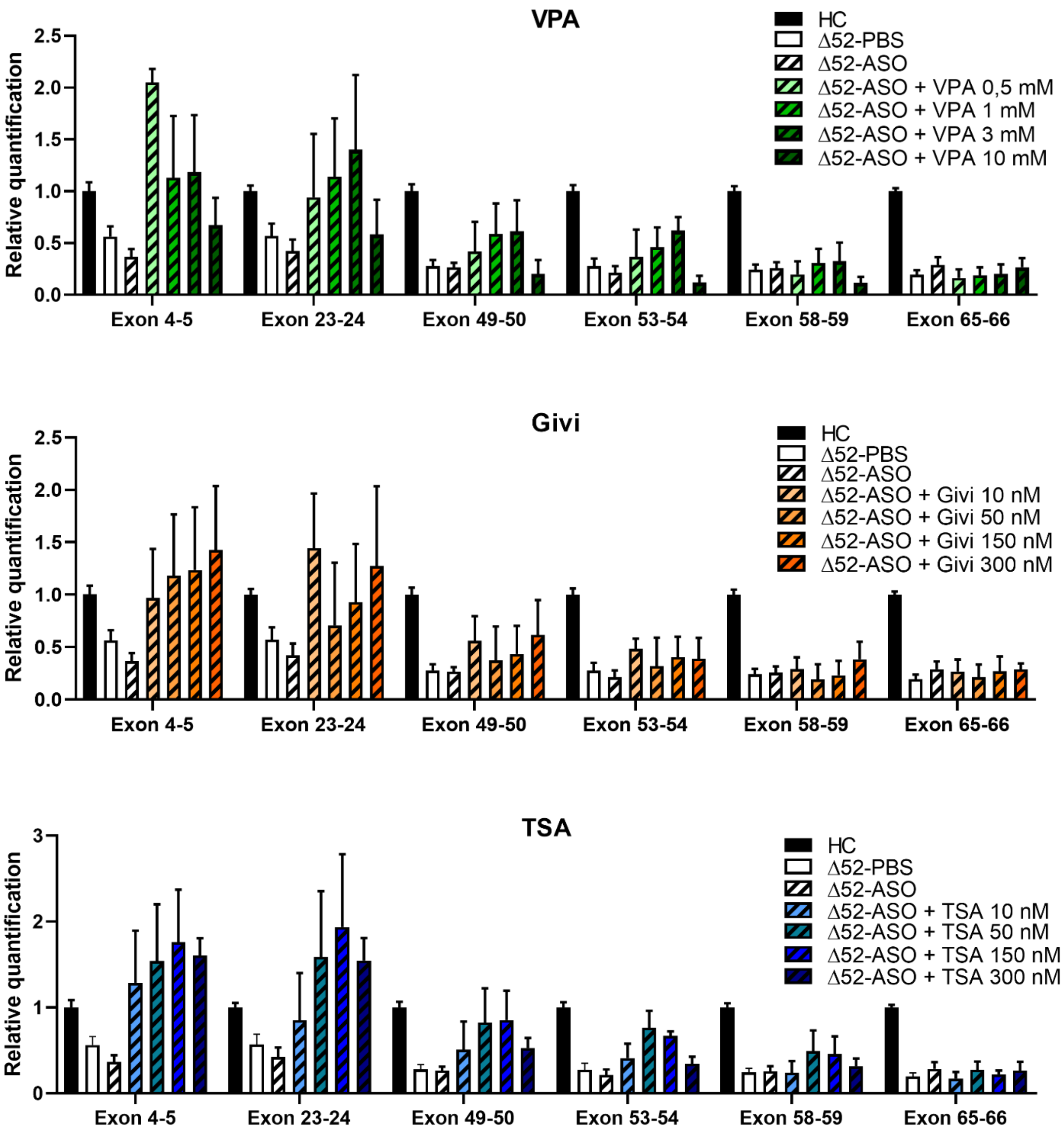

Figure S4. 5′–3′ *Dmd* transcript imbalance following HDACi+ASO treatment in murine cells. ddPCR quantification of *Dmd* transcripts across exon–exon junctions in murine myoblasts (n=3/group) treated with ASO and increasing HDACi concentrations. White = untreated; green, orange, blue = VPA, Givi, TSA; hatched = ASO-treated; black = healthy control (HC). Values normalized to *GAPDH*.

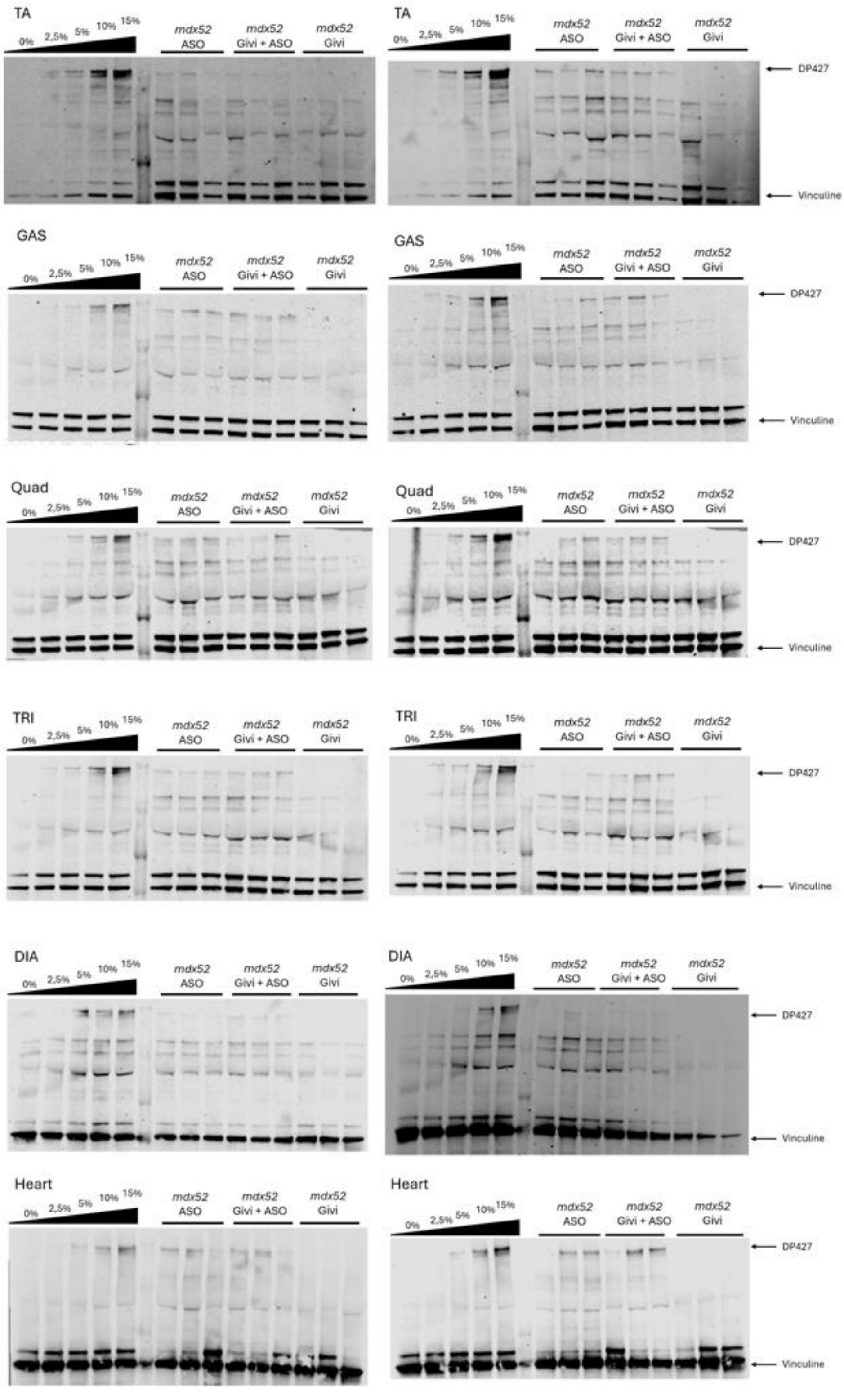

Figure S5. Western blot analysis of dystrophin restoration across muscles after 12-week Givi and/or ASO treatment. All western blot showing vinculin for normalization and dystrophin (DP427) restoration following 12-weeks of Givi or ASO treatment or combined therapy Givi + ASO in different muscles (TA, GAS, Quad, Tri, Dia, Heart). A standard curve was generated using mixtures of WT and *mdx52*-PBS protein lysates containing 0–15% dystrophin. N=6 per group.

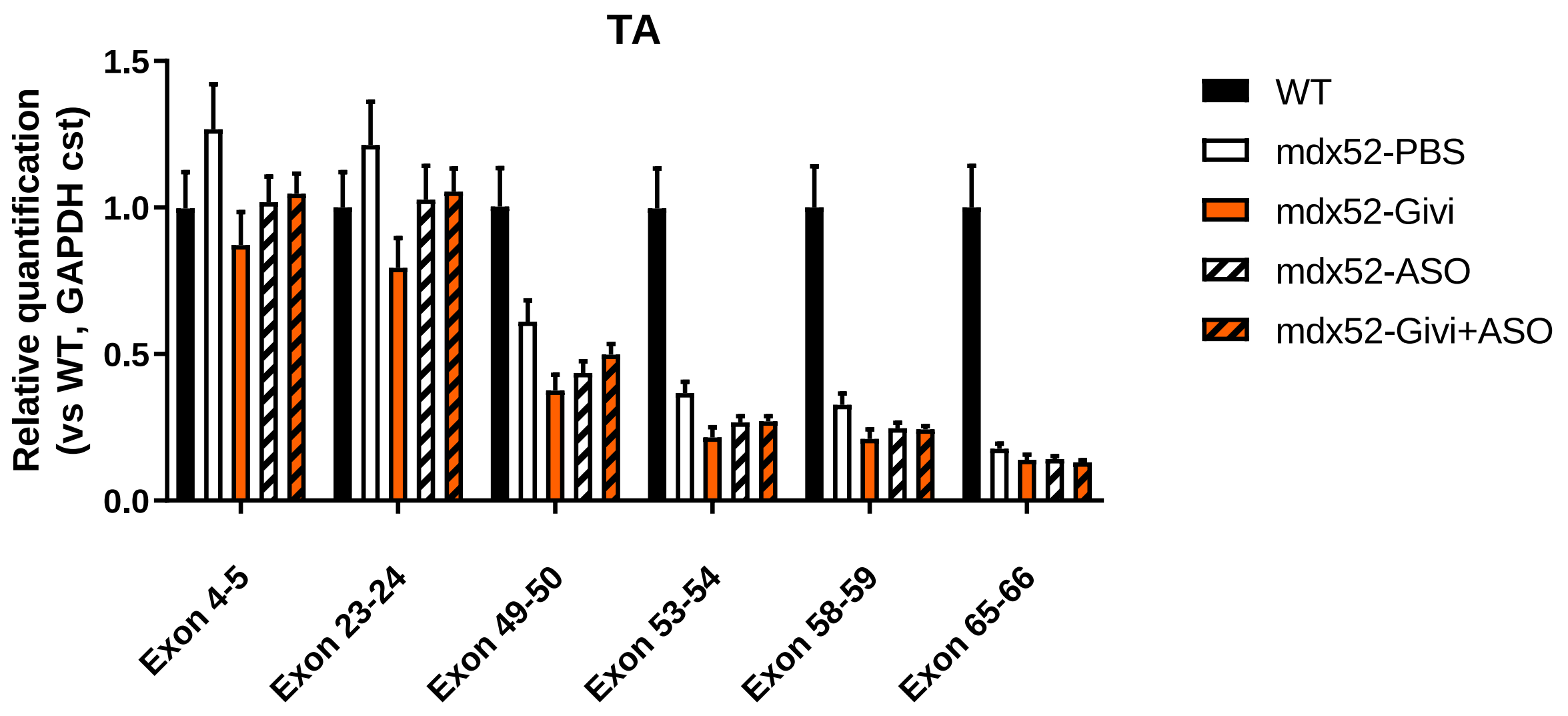

Figure S6. ***Dmd*** **transcript expression in tibialis anterior after 12-week treatment.**

Dmd expression in tibialis anterior (TA) was analyzed by ddPCR at various exon–exon junctions in *WT* and *mdx52* mice (n = 6/group) treated with saline, Givinostat (Givi), ASO, or Givi+ASO. Data were normalized to GAPDH and relative to WT. No significant differences were detected between HDACi-treated and saline groups (two-way ANOVA).

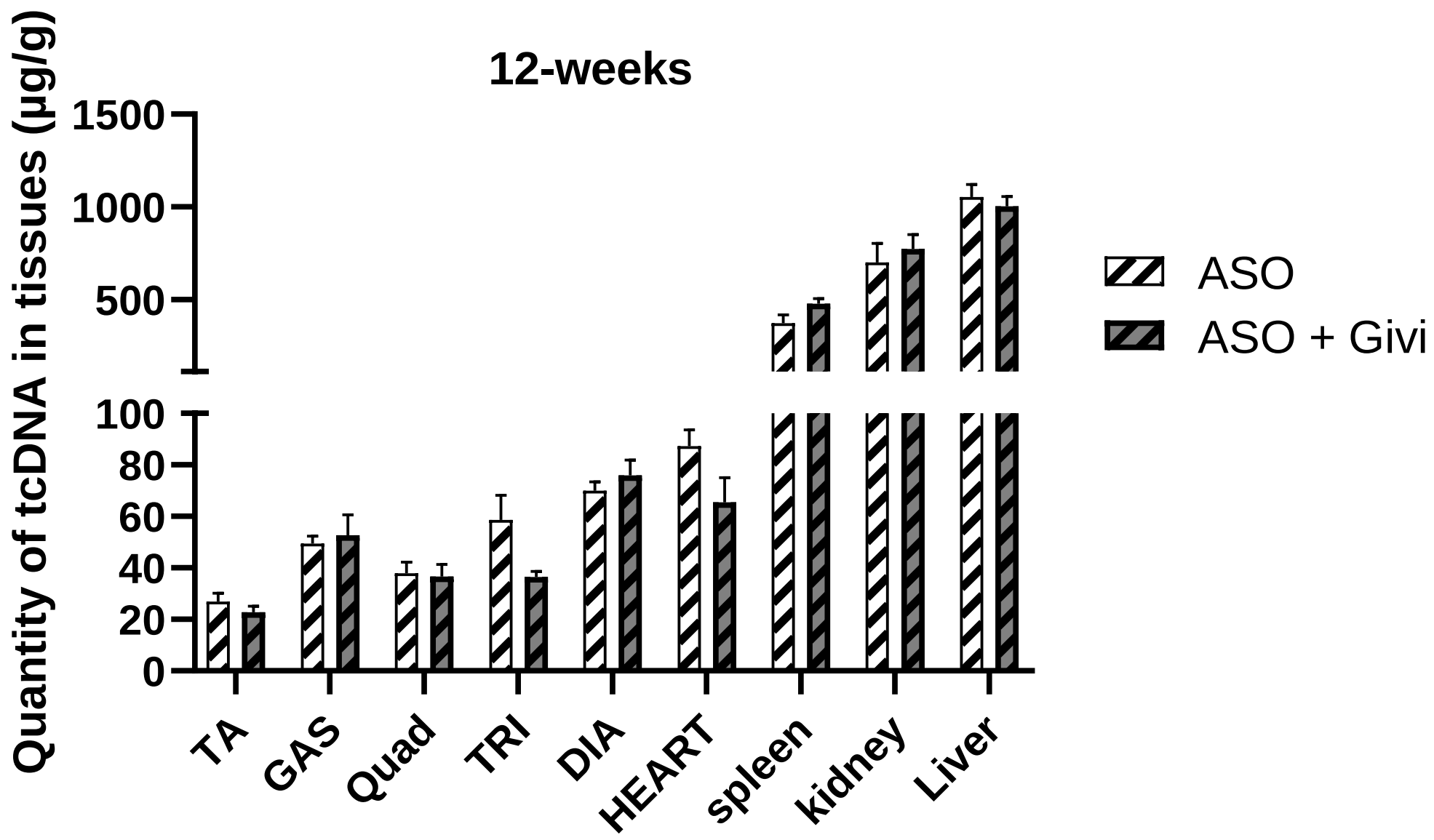

Tissue	TA	GAS	Quad	TRI	Dia	Heart	Spleen	Kidney	Liver
ASO vs Givi+ASO (*p* value)	> 0,9999	> 0,9999	> 0,9999	> 0,9999	> 0,9999	> 0,9999	0,4236	0,8358	0,9846

Figure S7. Quantification of ASO biodistribution in muscle and major organs after 12 weeks. ASO levels were measured in TA, gastrocnemius (GAS), quadriceps (QUAD), triceps (TRI), diaphragm (DIA), heart, and in spleen, liver, and kidney (n = 6/group). No statistical difference was detected between the ASO alone group and the ASO+Givi group using a two-way ANOVA (p = 0.5908). For full transparency, the table below reports the p values from Sidak's multiple comparisons post-hoc test following a two-way ANOVA comparing ASO and ASO+Givi treatments for each tissue after 12 weeks of treatment (n = 6/group).

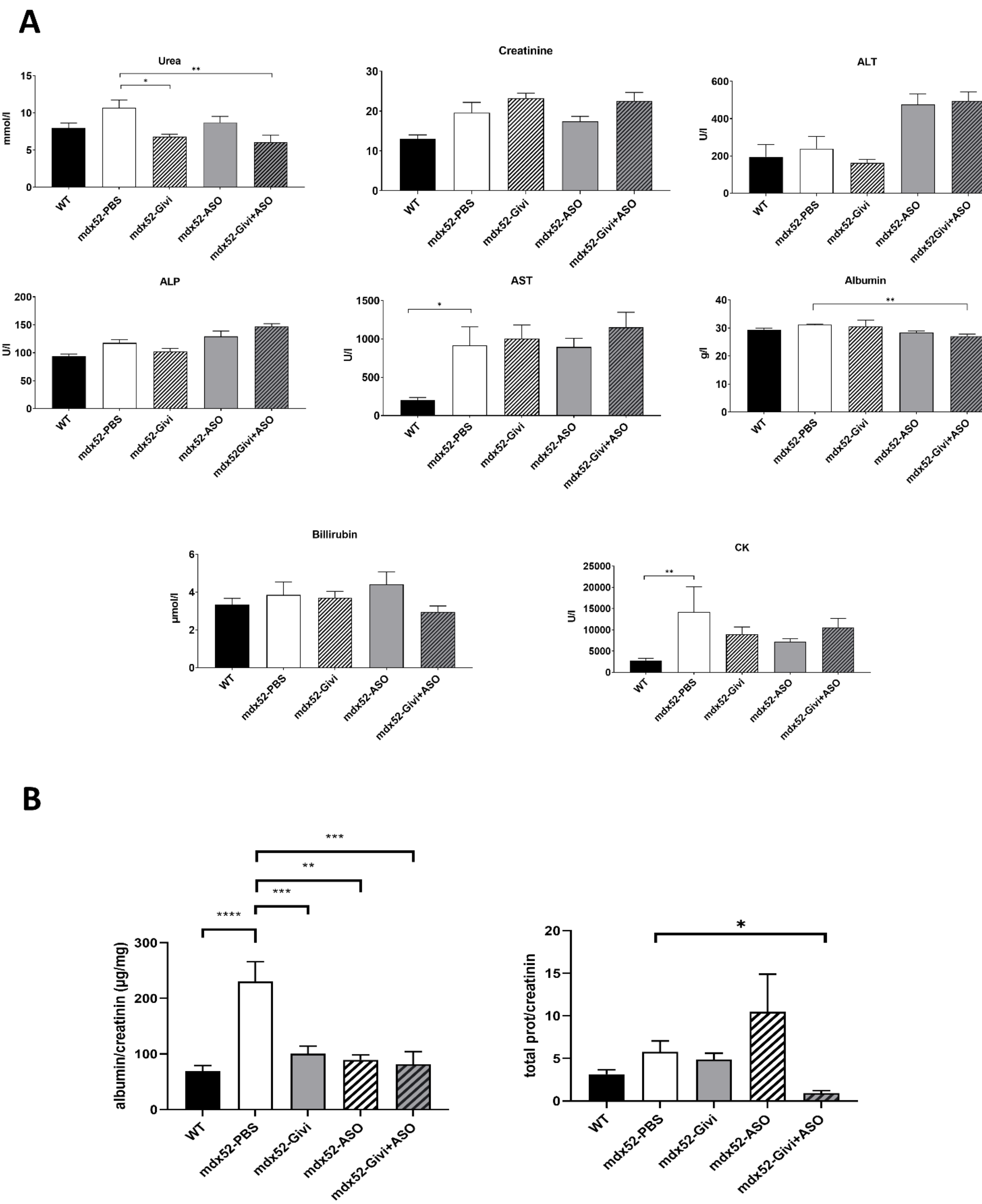

Figure S8. Evaluation of toxicity profile (A) Quantification of general toxicity biomarkers in serum collected two weeks after the last ASO injection. Serum levels of urea, creatinine, ALT, ALP, AST, albumin, bilirubin, and CK were measured in all groups (n = 6 mice per group). Urea levels differed significantly between *mdx52*-PBS and *mdx52*-Givi (p = 0.0291) as well as between *mdx52*-PBS and *mdx52*-Givi+ASO (p = 0.007; Kruskal–Wallis test). AST levels showed a significant difference between WT and *mdx52*-PBS (p = 0.0388; Kruskal–Wallis test). A significant difference in albumin levels was observed between *mdx52*-PBS and *mdx52*-Givi+ASO (p = 0.0012; Kruskal–Wallis test). CK levels were

significantly different between WT and *mdx52*-PBS ($p = 0.0054$; Kruskal–Wallis test). **(B)** Urine analysis. Albumin-to-creatinine ratio (left panel) and total protein-to-creatinine ratio (right panel) were measured in WT and *mdx52* mice treated with saline solution, ASO, Givinostat (Givi), or Givi+ASO (n = 6 per group). For the albumin-to-creatinine ratio, significant differences were observed between WT and *mdx52*-PBS ($p < 0.0001$) and between *mdx52*-PBS and treated groups (Givi: $p = 0.0009$; ASO: $p = 0.0026$; Givi+ASO: $p = 0.0002$; one-way ANOVA). For the total protein-to-creatinine ratio, a significant difference was detected only between *mdx52*-PBS and *mdx52*-Givi+ASO ($p = 0.0365$; one-way ANOVA).
