# Supplementary material for: Givinostat enhances antisense oligonucleotide efficacy in the mdx52 model of Duchenne muscular dystrophy
Source: Mol Ther Nucleic Acids. 2026 Aug 3;37(3):103047. doi: 10.1016/j.omtn.2026.103047 (PMC13521170; doi:10.1016/j.omtn.2026.103047)
Supplement: Supplementary file 2 [file mmc2.pdf]

Molecular Therapy
Nucleic Acids
Original Article

Givinostat enhances antisense oligonucleotide efficacy in the *mdx52* model of Duchenne muscular dystrophy

Xaysongkhame Phongsavanh,[1] Cecile Gastaldi,[2,3] Astrid Mottais,[1] Mathilde Doisy,[1] Olivier Le Coz,[1] Sandra de Haan,[4] Pietro Spitali,[4] Luis Garcia,[3,5] and Aurélie Goyenvalle[1,3]

[1]Université Paris-Saclay, UVSQ, Inserm, IMPROVE, 78000 Versailles, France; [2]Medical Biology Department, Centre Scientifique de Monaco, 98000 Principality of Monaco, MC, Monaco; [3]LIA BAHN, CSM-UVSQ, Principality of Monaco, Monaco; [4]Department of Human Genetics, Leiden University Medical Center, Leiden ZA 2333, the Netherlands; [5]SQY Therapeutics, UVSQ, 78180 Montigny le Bretonneux, France

Duchenne muscular dystrophy (DMD) is a severe X-linked neuromuscular disorder caused by mutations in the *DMD* gene, with deletions within the exon 45–55 hotspot being the most common. Despite advances in antisense oligonucleotide (ASO)-mediated exon skipping, therapeutic efficacy remains limited by suboptimal transcript availability and delivery barriers. We investigated whether histone deacetylase inhibitors (HDACis), particularly givinostat, can enhance the efficacy of an exon 51-targeting ASO in the clinically relevant *mdx52* mouse model. For the first time, we identified a pronounced 5′–3′ DMD transcript imbalance associated with exon 52 deletion, contrasting with the classical *mdx* model and potentially compromising ASO effectiveness. In human DMD myoblasts carrying an exon 52 deletion, givinostat significantly improved exon skipping and dystrophin restoration. *In vivo*, givinostat and ASO co-treatment resulted in a modest but significant increase in dystrophin levels compared to ASO alone (1.3-fold), along with reduced muscle fibrosis, improved fiber morphology, and better extracellular matrix organization. Notably, treated mice showed improved muscle function, as evidenced by a significant reduction in force loss after eccentric contractions, a clinically meaningful outcome. Although the precise molecular mechanisms underlying these effects remain to be fully elucidated, these findings support the therapeutic potential of combining givinostat with ASOs to enhance dystrophin restoration and muscle preservation in DMD.

INTRODUCTION

Duchenne muscular dystrophy (DMD) is an X-linked genetic disorder and one of the most prevalent conditions affecting children and young adults. This severe, debilitating, and ultimately fatal disease occurs in approximately 1:3,500–1:5,000 boys. Affected individuals typically become wheelchair-bound by their teenage years, with death resulting from respiratory failure or cardiomyopathy. However, with supportive therapies and corticosteroid treatment, life expectancy can extend beyond 20 years.[1] The disorder is caused by different types of mutations in the *DMD* gene encoding dystrophin, a protein essential for muscle fiber integrity,[2,3] thus leading to an absence of functional dystrophin protein. The dystrophin gene is among the largest genes in the human genome, spanning approximately 2.1 million base pairs and comprising 79 exons, with thousands of recorded mutations.[4] Notably, 70% of DMD patients carry deletions within a mutation-prone region, often referred to as the "hot-spot," located in the central part of the gene (exons 45–53).[3,5,6] Dystrophin plays a fundamental mechanical and structural role in both skeletal and cardiac muscle by linking the intracellular contractile apparatus to the sarcolemma. Specifically, it connects filamentous actin (F-actin) in the cytoskeleton to the dystrophin-associated protein complex (DAPC), a transmembrane glycoprotein assembly that anchors muscle fibers to the extracellular matrix via proteins such as laminin. This structural arrangement confers essential mechanical stability during muscle contraction.[7–9] The lack of dystrophin leads to sarcolemma fragility, followed by muscle necrosis, and in the later stages, the replacement of muscle tissue with fibrous tissue. Moreover, disruption of the DAPC leads to the mislocalization of muscle-specific nitric oxide synthase (NOS), an enzyme essential for the intramuscular production of nitric oxide and for the regulation of microRNAs (miRNAs) involved in muscle integrity and regeneration. The resulting decrease in nitric oxide levels induces aberrant and sustained hyperactivity of histone deacetylases (HDACs), which in turn causes excessive removal of acetyl groups from histone proteins. This promotes a heterochromatic state that silences key genes required for muscle damage repair.[10–12] While the underlying pathophysiology remains incompletely understood, various alternative mechanisms have been proposed, including disruptions in cell signaling, vascular adaptations, inflammatory responses, and repair processes.[13–15] Chronic inflammation, muscle weakness, and degeneration are key features of the disease.[16] Although significant progress has been made with genetic

Received 22 August 2025; accepted 30 July 2026;
https://doi.org/10.1016/j.omtn.2026.103047.

Correspondence: Aurélie Goyenvalle, Université Paris-Saclay, UVSQ, Inserm, IMPROVE, 78000 Versailles, France.
E-mail: aurelie.goyenvalle@uvsq.fr

© 2026 The Author(s). Published by Elsevier Inc. on behalf of The American Society of Gene and Cell Therapy.
This is an open access article under the CC BY license (http://creativecommons.org/licenses/by/4.0/).

approaches aimed at restoring dystrophin or its function, targeting secondary pathological mechanisms, such as preventing fibrosis or inflammation, is crucial to improve the efficacy of therapeutic interventions. These approaches must address both the genetic basis of the disease and its downstream consequences.

One of the most promising therapeutic approach for DMD is the antisense-mediated exon-skipping, which aims to eliminate one or several exons from the mRNA, by masking key splicing sites with antisense oligonucleotides (ASOs) during the pre-mRNA splicing process. The resulting mRNA will have a restored reading frame and therefore allow the expression of a BMD (Beker muscular dystrophin)-like dystrophin. Several ASO-based exon-skipping drugs have already been approved by the US FDA for the treatment of DMD (eteplirsen, golodirsen, viltolarsen, and casimersen targeting exons 51, 53, 53, and 45).[17] Approval was based on small but measurable increases in dystrophin expression (usually in the 1%–5% range) in muscle biopsies. To improve dystrophin restoration, extensive research has focused on enhancing ASO biodistribution to target tissues,[18–20] either through the development of alternative ASO chemistries or by conjugating ASOs with other molecules to facilitate their delivery to their muscle target.[21,22] Among these approaches, tricyclo-DNA (tcDNA) chemistry conjugated with palmitic acid has recently demonstrated promising potential in promoting dystrophin expression[23] and these ASOs are currently under clinical evaluation (NCT05753462). However, another challenge, far less studied than ASO biodistribution in target tissues, is the reduced availability of dystrophin transcripts in DMD patients. This phenomenon was previously attributed to the degradation of premature termination codon-carrying transcripts and reduced synthesis of nascent RNA.[24–26] Quantification of *DMD* transcripts along the coding sequence leads to an evident 5′–3′ imbalance.[26,27] Given that the *DMD* gene is one of the largest genes in the human genome (2.1 Mb), one hypothesis is that HDAC overexpression promotes a heterochromatic state that silences transcription, leading to premature disengagement of RNA polymerase II and preventing full-length transcription of the *DMD* gene. A similar, yet milder, 3′-end transcript loss can be detected in healthy individuals, suggesting a baseline physiological phenomenon.[28] Previous studies using cycloheximide (CHX), an inhibitor of nonsense-mediated decay (NMD), a quality control mechanism for mRNA in eukaryotic cells, have shown that NMD is only partially responsible for the observed reduction in *DMD* transcript levels.[27,29] In addition to HDAC overexpression observed in DMD models, studies have also reported increased expression of histone methyltransferases (HMTs), which often act in concert with HDACs to promote the deposition of repressive methyl groups on histone tails. In the *mdx* mouse model, this epigenetic dysregulation leads to elevated levels of H3K9me3 (trimethylation of lysine 9 on histone H3), a transcriptionally repressive histone mark.[27] This modification is found to be significantly enriched across the *Dmd* gene in *mdx* mice compared to control animals.

In this context, the use of histone deacetylase inhibitors (HDACis) may promote histone acetylation and thereby limit the deposition of transcriptionally repressive epigenetic marks, such as H3K9me3. By shifting the chromatin state toward a more permissive euchromatin configuration, HDACi may help restore the 5′–3′ transcript imbalance observed in DMD, ultimately increasing the abundance of *DMD* transcripts that serve as targets for exon-skipping therapeutic strategies.

Although HDACi have been developed for years and for various diseases, givinostat (Givi) (a pan-HDAC inhibitor) is the first non-steroidal molecule approved for the treatment of DMD.[30] Rather than targeting the genetic cause of the disease (mutations in the *DMD* gene), givinostat addresses downstream pathological consequences of dystrophin deficiency by reducing inflammation and fibrosis. Preclinical and clinical studies have demonstrated its ability to preserve muscle tissue, improve function, and slow disease progression in boys with DMD.[31,32] Based on these results, the FDA recently approved Givi (Duvyzat) for DMD, while it was conditionally approved by the EMA (European Medicines Agency).

We hypothesize here that on top of these beneficial effects, HDACi could significantly improve the effect of exon skipping strategies by increasing the level of *DMD* transcript. Our recent studies have highlighted the significant impact of 5′–3′ *Dmd* transcript imbalance in the *mdx* mouse model across various tissues, which could affect the efficacy of ASO technology.[33] We have also demonstrated that certain HDACi, such as Givi and valproic acid (VPA), can partially correct the 5′–3′ transcript imbalance of *Dmd* mRNA and thereby enhance dystrophin restoration when combined with an ASO in *mdx* mice. Moreover, additional studies have shown that long-term HDACi treatment (12-weeks VPA treatment) not only enhances dystrophin restoration across various muscle tissues but also leads to significant improvements in functional outcomes in *mdx* mice.[34] Yet, given the relatively proximal location of the mutation in *mdx* mice (nonsense mutation in exon 23), we hypothesize that the 5′–3′ transcript imbalance could have a greater impact on exon-skipping strategies targeting more distal regions, particularly within the hotspot spanning exons 45–55. This is particularly pertinent for DMD patients and may explain the low dystrophin expression often measured in clinical trials following exon 51 skipping.

In this study, we used the *mdx52* mouse model,[35] which carries a deletion in exon 52 located within a common mutation hotspot in the *DMD* gene,[3,5,6] making it highly relevant for testing exon 51-targeted therapies. For the first time, we show that the *mdx52* mouse displays a more exacerbated 5′–3′ *Dmd* transcript imbalance than the *mdx* model across various muscle and brain tissues. We then identified Givi as the most promising HDACi compared to VPA and trichostatin A (TSA). *In vivo*, combined treatment with Givi and exon 51-targeting ASOs modestly but significantly enhanced dystrophin expression and improved muscle histopathology and function in *mdx52* mice, without detectable adverse effects. Our findings support an additive benefit of combining Givi with ASO51, highlighting its translational potential for DMD patients, as both therapeutic approaches are already FDA-approved.

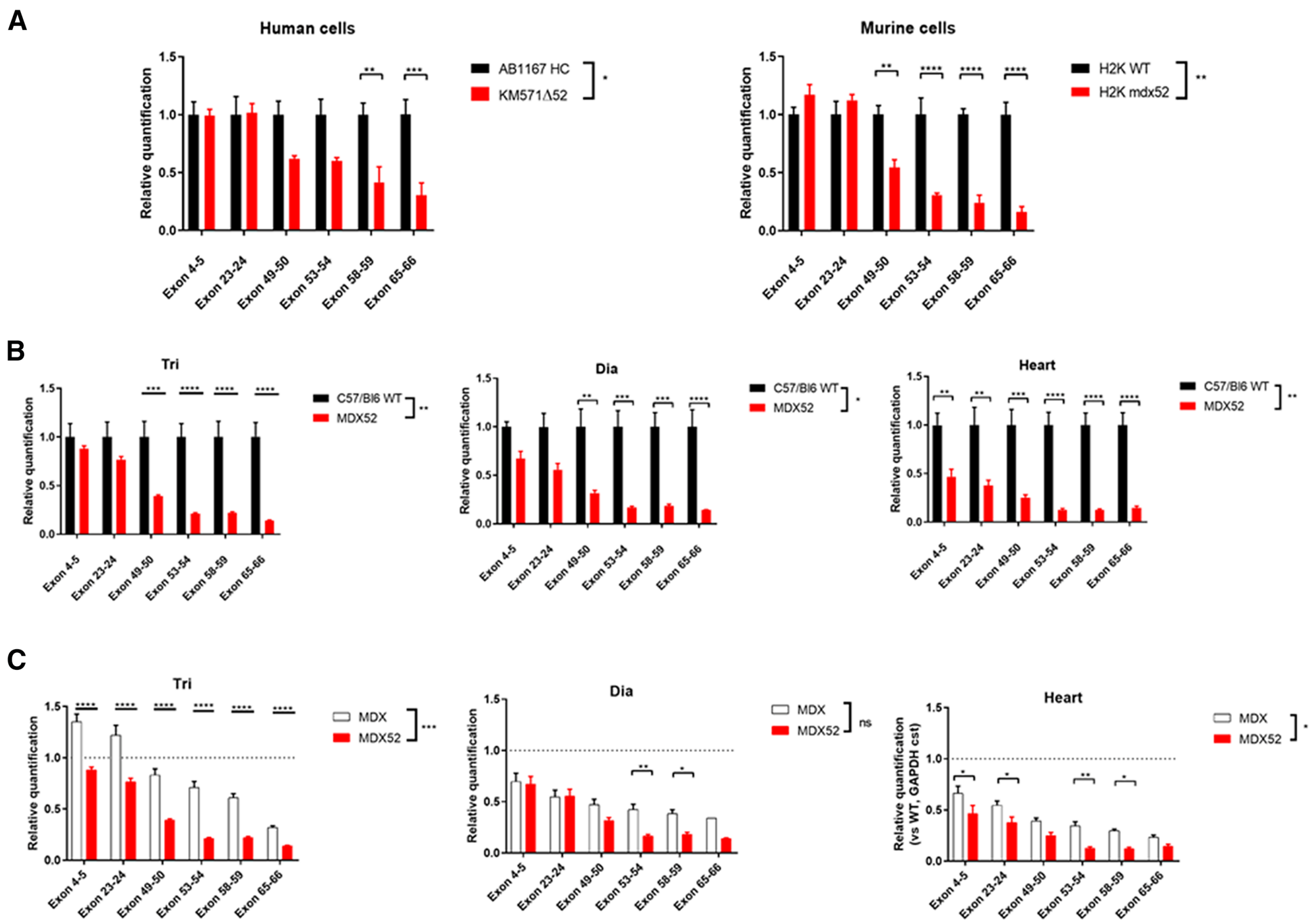

Figure 1. *In vitro* and *in vivo* characterization of 5′–3′ *DMD* transcript imbalance

(A) Analysis of *DMD* transcript imbalance in human (AB1167 HC, KM571Δ52) and murine (H2KWT, H2K*mdx52*) myotubes. Relative expression of *Dmd* transcripts was quantified at multiple exon-exon junctions by qPCR in *KM571Δ52* (n = 4) and *AB1167 HC* (n = 4) human myotubes, and in H2K*mdx52* (n = 4) and H2KWT (n = 4) murine myotubes. Values are normalized to GAPDH and expressed relative to wild-type levels. Statistical analysis was performed using two-way ANOVA, *$p < 0.05$, **$p < 0.01$, ***$p < 0.001$, and ***$p < 0.0001$. Significant differences were observed between AB1167 HC versus KM571Δ52 (p = 0.0445) and H2KWT versus H2K*mdx52* (p = 0.0013). (B) Tissue-specific assessment of *Dmd* transcript imbalance in *mdx52* (red bars) and *C57BL/6* wild-type (black bars) mice. Relative expression was measured across several exon-exon junctions in the triceps, diaphragm, and heart (n = 3 per group). Data were normalized to GAPDH and expressed relative to wild-type. Significant differences were observed in triceps (p = 0.0034), diaphragm (p = 0.0108), and heart (p = 0.0062), as determined by two-way ANOVA. (C) Comparison of *Dmd* transcript imbalance between *mdx* (white bars) and *mdx52* (red bars) mice. Expression was analyzed across multiple exon-exon junctions in the triceps, diaphragm, and heart (n = 3 per group). Significant differences were observed in triceps (p = 0.0004) and heart (p = 0.0203); diaphragm differences were not significant (p = 0.0769), analyzed by two-way ANOVA. *$p < 0.05$, **$p < 0.01$, ***$p < 0.001$, and ***$p < 0.0001$.

RESULTS

Characterization of the 5′–3′ *DMD* transcript imbalance in models carrying an exon 52 deletion

We first examined *DMD* gene expression at various exon-exon junctions in relevant human and murine *in vitro* models. Human healthy control (HC) myoblasts (AB1167 HC) and DMD myoblasts carrying a deletion of exon 52 (KM571Δ52) were differentiated into myotubes and RNA was analyzed. Quantitative PCR (qPCR) analysis revealed a progressive decrease in *DMD* transcript levels from the 5′ to the 3′ end of the gene, with up to a 70% reduction in transcript abundance observed at the exon 65–66 junction in Δ52 myotubes compared to wild-type (WT) (p = 0.0003 analyzed by two-way ANOVA) (Figure 1A). A global comparison of all exon-exon junctions also revealed a significant reduction in transcript levels in Δ52 versus WT myotubes (p = 0.0445 genotype effect analyzed by two-way ANOVA). The same experiment was performed in murine WT myoblasts (H2K WT) and exon 52-deleted myoblasts (H2K *mdx52*), also revealing a 5′-to-3′ *Dmd* transcript imbalance, which was even more pronounced. A reduction of up to 84% in *Dmd* transcript levels was observed at the most distal exon-exon junctions compared to WT myotubes (p = 0.0013). These *in vitro* models carrying an exon 52 deletion (clinically relevant and translatable to human DMD) represent valuable systems for studying the effects of HDACi and ASOs in this genetic context. We next characterized the 5′-to-3′ *Dmd* transcript imbalance *in vivo* in the *mdx52* mouse model compared to their C57BL/6 WT controls. qPCR analyses were performed on skeletal and cardiac muscles (triceps [TRI], diaphragm [DIA], heart) of 3-month-old *mdx52* mice, revealing a significant transcript imbalance across the *Dmd* gene in all three muscle types (p = 0.034 for TRIs, p = 0.0108 for DIA, and p = 0.0062 for heart) (Figure 1B). To better visualize this 5′-to-3′ transcript imbalance, we also plotted *Dmd* transcript amplification levels at various exon junctions using

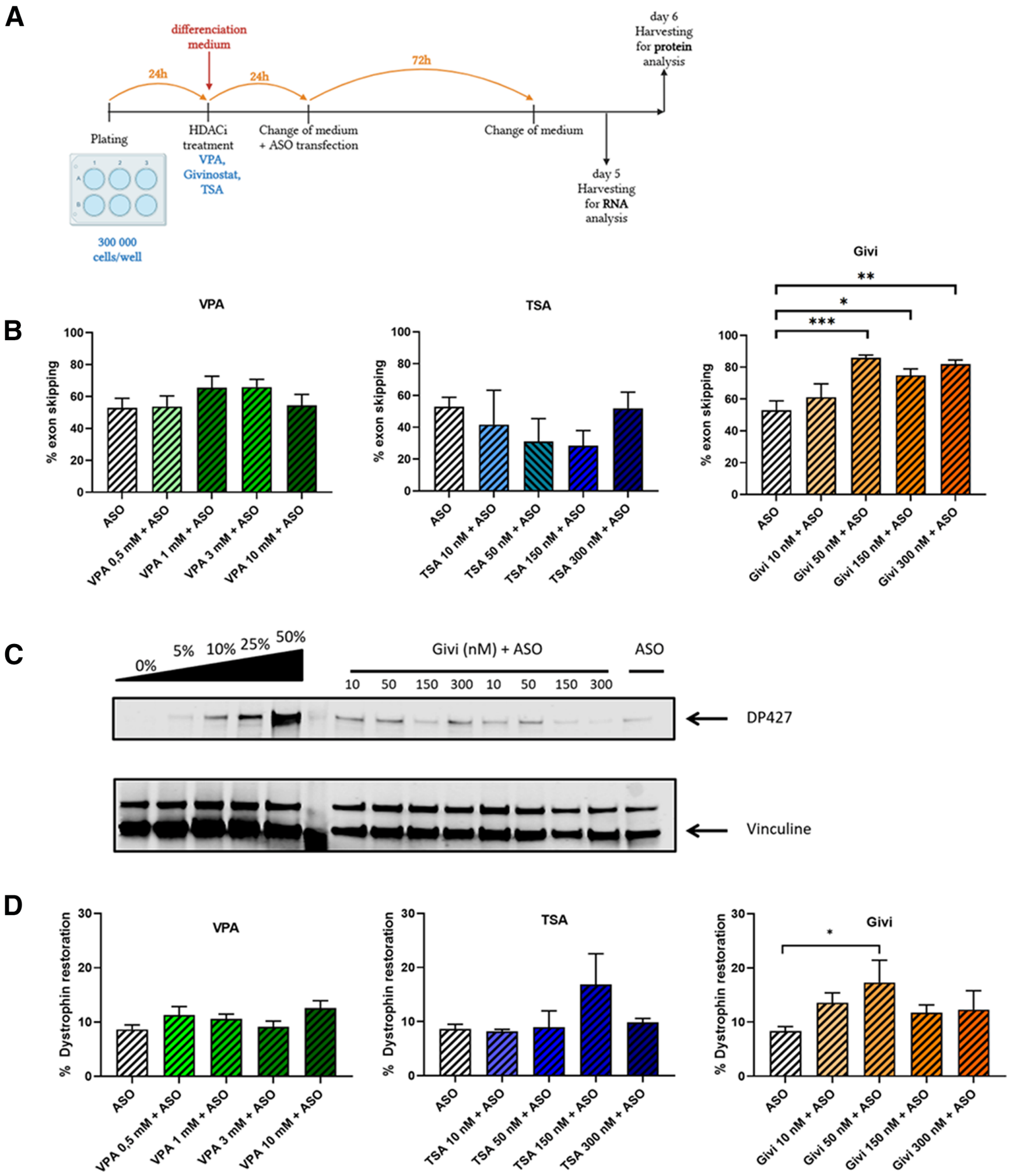

Figure 2. Effect of combined therapy in human cells

(A) Schematic overview of the cell culture protocol used for RNA and protein analysis. Human myoblasts were plated at 300,000 cells/well, cultured in proliferation medium for 24 h, treated with HDACi for another 24 h, and then induced to differentiate for 5 or 6 days (depending on RNA or protein analysis). (B) Digital droplet PCR (ddPCR) quantification of exon skipping efficiency in KM571Δ52 cells (n = 6/group) following ASO and HDACi treatment. Hatched bars = ASO-treated; green, blue and orange = valproic acid (VPA), trichostatin A (TSA) and givinostat (Givi), respectively. Each condition was performed in triplicate. Significant differences were only observed between ASO and ASO combined with Givi 50, 150, and 300 nM with a p value of 0.0005, 0.0197, and 0.0018, respectively, analyzed by one-way ANOVA. (C) Representative western blot showing dystrophin restoration in human myotubes after ASO or Givi + ASO treatment. Vinculin = loading control. A standard curve (0%–50% dystrophin) was generated by mixing AB1167 HC and KM571Δ52 lysates. (D) Quantification of dystrophin restoration (Empiria Studio). Hatched = ASO-treated, green = VPA, blue = TSA, orange = Givi; n = 7 (VPA + ASO, Givi + ASO), n = 3 (ASO + TSA), n = 10 (ASO-only). Givi + ASO (50 nM) significantly increased dystrophin vs. ASO (p = 0.0261 analyzed by one-way ANOVA, mean difference = 8.964 ± 3.397 (SEM), 95% confidence interval, CI: 1.763 à to 16.17, partial η^2 (effect sizes) = 0.3032, analyzed by t test).

linear regression in TRIs, DIA, and heart (Figure S1A). A similar transcript imbalance was detected in brain tissues (cerebellum, hippocampus, and cortex), with significant differences between *mdx52* and WT observed in cerebellum (p = 0.0001), hippocampus (p = 0.0497), and cortex (p = 0.0188) (Figure S1B). However, in brain tissues, expression of the Dp71 isoform (known to be highly expressed in the brain) was detected at the most distal exon junctions (exon 65–66), partially explaining the maintained 3′ transcript signal. As previously described in the literature, a natural 5′-to-3′ transcript imbalance is also present in WT mice, likely due to the extreme length of the *DMD* gene[27] (Figure S1A). Notably, the deletion of exon 52 in the *mdx52* model shifts this regression line downward, exacerbating the 5′-to-3′ transcript imbalance. Finally, we compared the 5′-to-3′ *Dmd* transcript imbalance between the *mdx52* model and the reference *mdx* model (which carries a nonsense mutation in exon 23) (Figure 1C). We observed that the *mdx52* mice exhibit a more pronounced transcript imbalance than the *mdx* mice in all muscles examined (TRIs [p = 0.0004], heart [p = 0.0203], and DIA [p = 0.0769]). Similar findings were observed in brain tissues between the *mdx* and the *mdx52* (Figure S1C). Overall, these data demonstrate that the *mdx52* mouse is a relevant model to study the effects of HDAC inhibitors and ASOs on DMD transcripts. The exacerbated 5′-to-3′ transcript imbalance in this model compared to the *mdx* model closely mimics the conditions observed in patients, making it particularly suitable for evaluating therapeutic interventions.

In vitro evaluation of the combined HDACi + ASO therapy

To investigate the effects of HDACi on human cells, we selected three HDACi (VPA, Givi, or TSA) and used human myoblasts *KM571Δ52* and their control AB1167 HC. After 24 h of treatment with HDACi at various doses, cells were transfected with an exon 51-targeting ASO for 3 days. The differentiation period was adapted depending on the type of analysis: 5 days for transcript analysis and 6 days for protein analysis, as only mature myotubes express dystrophin (Figure 2A). We checked the tolerability of these various doses of HDACi on human cells using the 3-(4,5-dimethylthiazol-2-yl)-2,5-diphenyltetrazolium bromide (MTT) viability assay, which revealed a slight toxicity of TSA treatment, while VPA and Givi were both well tolerated at all tested doses (Figure S2A). We assessed exon skipping efficiency by reverse-transcription PCR (RT-PCR) (Figure S3A) and RT-digital droplet PCR (ddPCR)

for more precise quantification (Figure 2B). VPA and TSA, when combined with the ASO targeting exon 51, did not produce a statistically significant increase in exon skipping efficiency compared to ASO treatment alone, although VPA showed a trend toward improvement. Only Givi, in combination with ASO, resulted in a significant enhancement of exon skipping efficiency, starting at the 50 nM dose, where the skipping efficiency was 1.6-fold higher than in cells treated with ASO alone. It is important to note that exon skipping efficiency is calculated as a percentage of total *DMD* transcripts and therefore may not fully capture the broader effects of HDACi on chromatin relaxation and transcript upregulation.

We next performed western blot analysis on treated cells to assess the levels of dystrophin restoration after the various treatments (a representative example of western blot is shown in Figures 2C and S3B). Quantification of the western blots (Figure 2D) revealed a trend toward increased dystrophin restoration with each dose of VPA, whereas TSA showed no significant effect, except for a slight trend at 150 nM. Notably, only the combination of 50 nM Givi with the exon 51-targeting ASO resulted in a significant increase in dystrophin restoration compared to ASO alone, with a 2-fold increase observed. Other Givi doses also showed a modest trend toward improvement. We next aimed to determine whether the significant increase in dystrophin restoration observed in human cells was due to a correction of the 5′-3′ *Dmd* transcript imbalance. To this end, we analyzed *DMD* transcript expression along the entire *DMD* gene at various exon junctions (Figure 3A) following treatment with different HDACi (VPA, Givi, and TSA) at varying concentrations. None of the HDACi succeeded in restoring the transcript imbalance. However, at the most distal junctions (exon 65–66), we observed a slight increase in *DMD* transcripts in human cells treated with VPA + ASO or Givi + ASO compared to ASO treatment alone. This modest increase may partially explain the higher levels of dystrophin restoration observed but does not account for the 2-fold increase seen at the optimal 50 nM dose of Givi combined with ASO. However, an improvement in the slope of this imbalance was observed (Figure 3B). A more negative slope indicates a higher 5′-to-3′ *DMD* transcript imbalance. ASO treatment alone appears to slightly improve the slope, and combining VPA or TSA with the exon 51-targeting ASO did not result in a significant improvement. Only Givi treatment led to a statistically significant improvement in the slope of the 5′-to-3′ *DMD* transcript imbalance, regardless of the dose used.

Given the increase in exon skipping and dystrophin protein with Givi treatment, we wondered whether treatment with Givi would increase dystrophin pre-mRNA levels and therefore increase the ASO target corresponding to an increase in exon skipping. To address this, we performed analysis of nascent RNA using bromouridine sequencing. Analysis of the data showed that coverage significantly decreased along the gene body ($p < 0.01$) and that DMD cells synthesized less nascent RNA compared to healthy cells ($p < 10^{-14}$) (Figure 3C, left). Interestingly, all 3 treatments increased the amount of nascent RNA (ASO, Givi, and ASO + Givi), with Givi showing the most significant effect ($p < 10^{-6}$), followed by ASO alone ($p < 0.01$) and the combination treatment ($p < 0.05$). This effect was not observed on other target genes such as *ACTB* or *GAPDH* (Figure 3C, middle and right). We next evaluated the effects of combining HDACi with the ASO in murine cells, using the same experimental protocol as for human cells. In murine cells, no toxicity was observed with any of the HDACi at any doses (Figure S2B). Exon skipping efficiency was quantified by ddPCR, revealing no significant enhancement across the various HDACi treatments, irrespective of the dose (Figure 4A). Only Givi exhibited a modest trend toward increased skipping efficiency. In contrast to human cells, HDACi did not increase the absolute levels of *Dmd* transcripts in murine cells (data not shown). We also performed western blot analyses of dystrophin expression in murine myotubes. No significant increase in dystrophin restoration was observed with the HDACi + ASO combination compared to ASO treatment alone. A slight trend toward improved dystrophin restoration was noted at 1 and 3 mM for VPA, and at 50 nM for TSA. For Givi, an increasing trend in dystrophin restoration was observed with each dose, although this did not reach statistical significance. Overall, these results are consistent with the exon skipping data as well as with the 5′-3′ *Dmd* transcript imbalance, which did not appear significantly corrected (Figure S4). Only at the most proximal exon junctions (exon 4–5), a substantial amount of *Dmd* transcripts was detected after 24 h of HDACi treatment, regardless of the HDACi used or its concentration (Figure 4C), suggesting that HDACi promote transcription at the proximal exon junctions of the *Dmd* gene. Yet, transcript levels at distal 3′ regions remain similar to those in untreated cells and no HDACi was able to significantly rescue the transcript imbalance in murine cells. Taken together, these *in vitro* results indicate that treatment with Givi was associated with increased exon skipping and dystrophin restoration in human myotubes, whereas in murine cells it induces only a modest, non-significant trend. These effects are not associated with a full correction of the 5′–3′ *Dmd* transcript imbalance; however, nascent RNA analyses indicate that Givi treatment is associated with an increase in *DMD* transcriptional output. Overall, these findings support a modulatory effect of Givi on ASO efficacy *in vitro* and provide a rationale for further evaluation *in vivo*.

In vivo evaluation of the combined treatment in dystrophic *mdx52* mice

Based on the *in vitro* results, the combination of Givi with an exon-skipping ASO appeared to be the most promising candidate for preliminary *in vivo* studies. We therefore conducted a pilot study in *mdx52* mice (Figure 5A), in which ASOs were administered intravenously (i.v.) (50 mg/kg/week) alongside daily oral gavage of Givi (10 mg/kg/day, 5 days/week) for 4 weeks. Molecular analyses were then performed on tissue samples from these animals. First, we assessed exon-skipping efficiency by droplet digital PCR (ddPCR) in various muscle tissues, including the tibialis anterior (TA), TRIs, DIA, and heart. No significant differences in exon-skipping efficiency were observed between mice co-treated with Givi and ASO compared to those treated with ASO alone (Figure 5B). We then

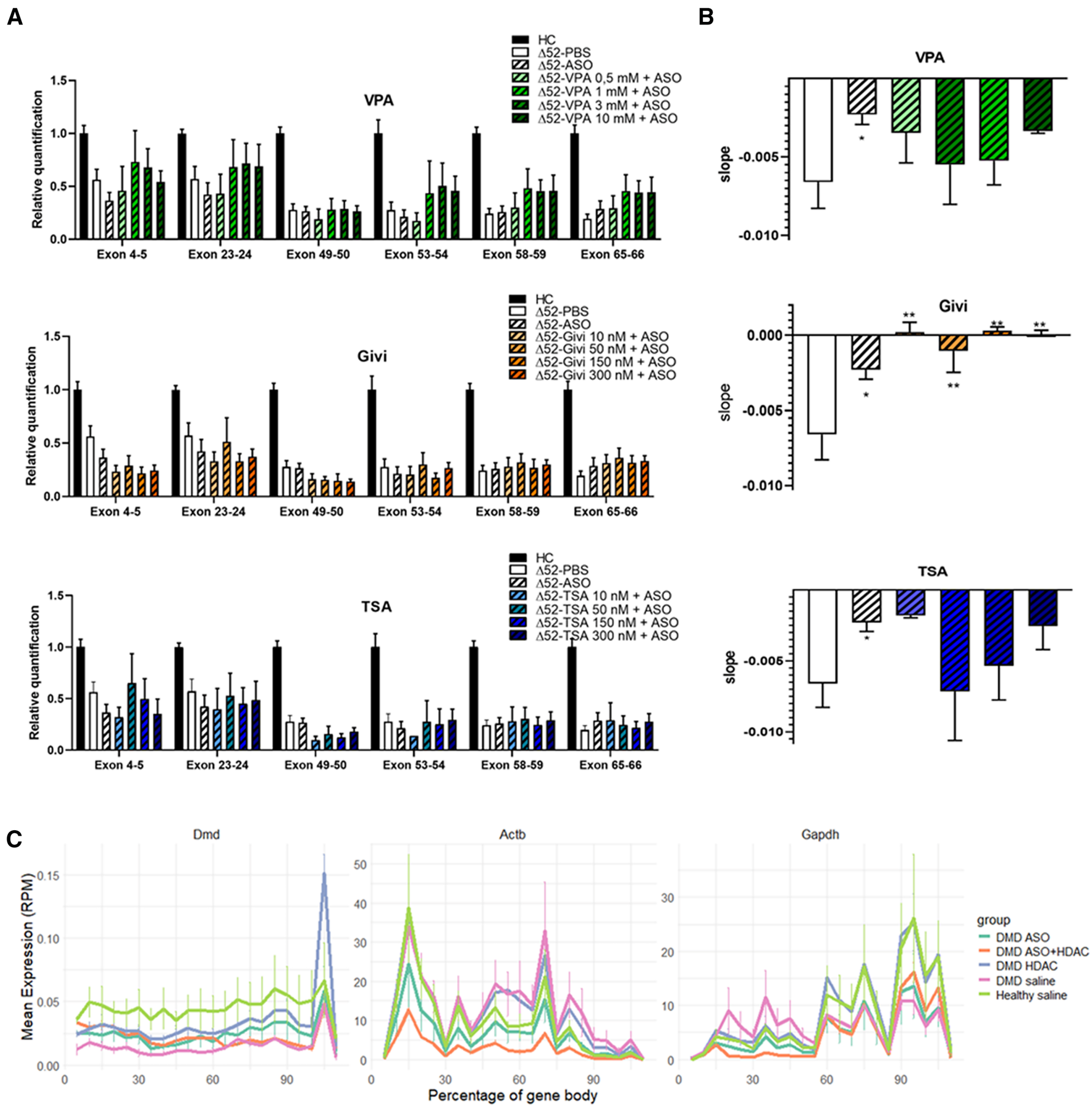

Figure 3. Effect of HDACi 5′–3′ *DMD* transcript imbalance and nascent RNA

(A) ddPCR quantification of *DMD* transcripts across exon-exon junctions in human myoblasts (n = 3/group) treated with ASO and increasing HDACi concentrations. Black = AB1167 healthy control (HC), white = KM571Δ52 treated with PBS; hatched = ASO-treated; green, orange, blue = VPA, Givi, TSA. Values normalized to GAPDH and expressed relative to HC. (B) Average slopes of linear regressions representing the 5′–3′ transcript imbalance across six exon-exon junctions. Each bar = average slope from 3 biological replicates per condition. Treatments: PBS, ASO, and HDACi + ASO (VPA 0.5–10 mM, Givi 10–300 nM, TSA 10–300 nM). Significant differences were observed between KM571Δ52 treated with ASO and PBS (p = 0.0356), between PBS-treated and human cells treated with ASO combined with Givi 10 nM or Givi 50 nM or Givi 150 nM or Givi 300 nM (p = 0.0015, p = 0.0073, p = 0.0014, p = 0.0020, respectively), analyzed by one-way ANOVA. (C) Line plots showing the coverage of nascent RNA detected by Bru-seq for *DMD*, *ACTB*, and *GAPDH*. The gene body is shown as percentage on the x axis while expression in reads per million (RPM) is plotted on the y axis. The lines represent the average of nascent RNA expression divided over 20 bins. Error bars represent standard error. The experiment was performed in duplicate. For *DMD* and *ACTB* promoters are located at the right end of each plot and the directionality of RNA synthesis is right to left, while for *GAPDH* the nascent RNA is synthesized left to right.

evaluated the percentage of dystrophin restoration in the same tissues and found a statistically significant increase in dystrophin restoration in the Givi + ASO group compared to the ASO-only group (p = 0.0159) (Figure 5C). However, the absolute levels of dystrophin remained very low, ranging from 0% to 4%, limiting the biological relevance of this difference. Importantly though, no alterations in liver or kidney markers were observed following treatment (Figure 5D). Interestingly, even after only 4 weeks of treatment and despite the low dystrophin restoration levels, a reduction in serum levels of myomesin-3, a biomarker of muscle damage, was detected in the Givi + ASO group compared to the ASO-only group (p = 0.0451) (Figure 5E). Following the encouraging findings from

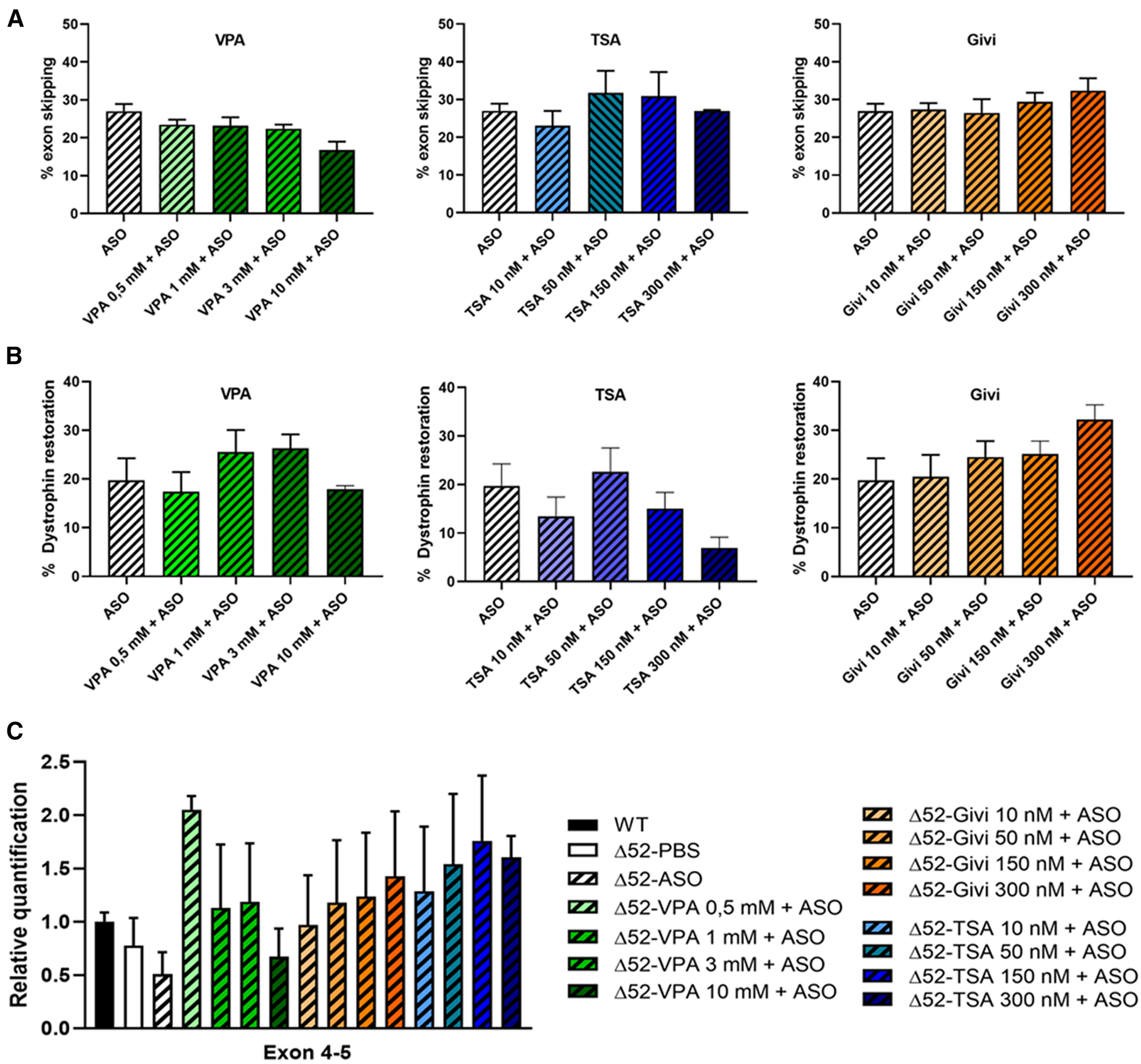

Figure 4. Effect of combined therapy in murine cells

(A) ddPCR quantification of exon 51 skipping in H2K*mdx52* myoblasts ($n = 6$/group for VPA and Givi-treated cells and $n = 3$/group for murine cells treated with TSA) after ASO and HDACi treatment following the same protocol as in Figure 2A. None of HDACi treatment reached statistical difference compared to saline, analyzed by one-way ANOVA. (B) Quantification of dystrophin restoration from western blots ($n = 6$/group for VPA and Givi treated cells and $n = 3$/group for murine cells treated with TSA) using Empiria Studio. Statistical analysis using one-way ANOVA did not reveal any significant differences. (C) ddPCR quantification of *Dmd* transcripts across exon-exon 4–5 junction in murine myoblasts ($n = 3$/group) treated with ASO and increasing HDACi concentrations. Values are normalized to *GAPDH* and expressed relative to WT controls.

the preliminary 4-week study combining Givi and an exon-skipping ASO, a 12-week treatment protocol was implemented, increasing the ASO dose to 75 mg/kg/week (to ensure higher levels of dystrophin rescue) while maintaining the same daily Givi dosage (10 mg/kg/day, 5 days/week) (Figure 6A). We first analyzed exon-skipping efficiency in samples from mice treated for 12 weeks with Givi + ASO and compared them to mice treated with ASO alone. Exon skipping was visualized by electrophoresis following RT-PCR as shown in Figure 6B and quantification using ddPCR revealed overall higher exon-skipping levels compared to the 4-week treatment, ranging from 5% to 20% (Figure 6C). However, no statistically significant difference in exon-skipping efficiency was observed between the ASO and Givi + ASO groups across all muscle tissues. We then investigated dystrophin restoration and performed western blot analyses on tissues from mice treated with either ASO alone or the Givi + ASO combination (Figures 6D and S5; Table S1). Overall, a modest but statistically significant increase in dystrophin expression was observed in the Givi + ASO group, with an average fold change of 1.3 compared to ASO alone ($p = 0.0444$). This increase was most notable in gastrocnemius (GAS) (2.7% in ASO vs. 4.22% in ASO + Givi), DIA (2.25% vs. 3.48%), and heart (5.21% vs. 7.26%) (Table S1). To validate these findings, dystrophin immunostaining was performed on the heart, DIA, and TRIs (Figure 6E), confirming the protein restoration observed by western blot.

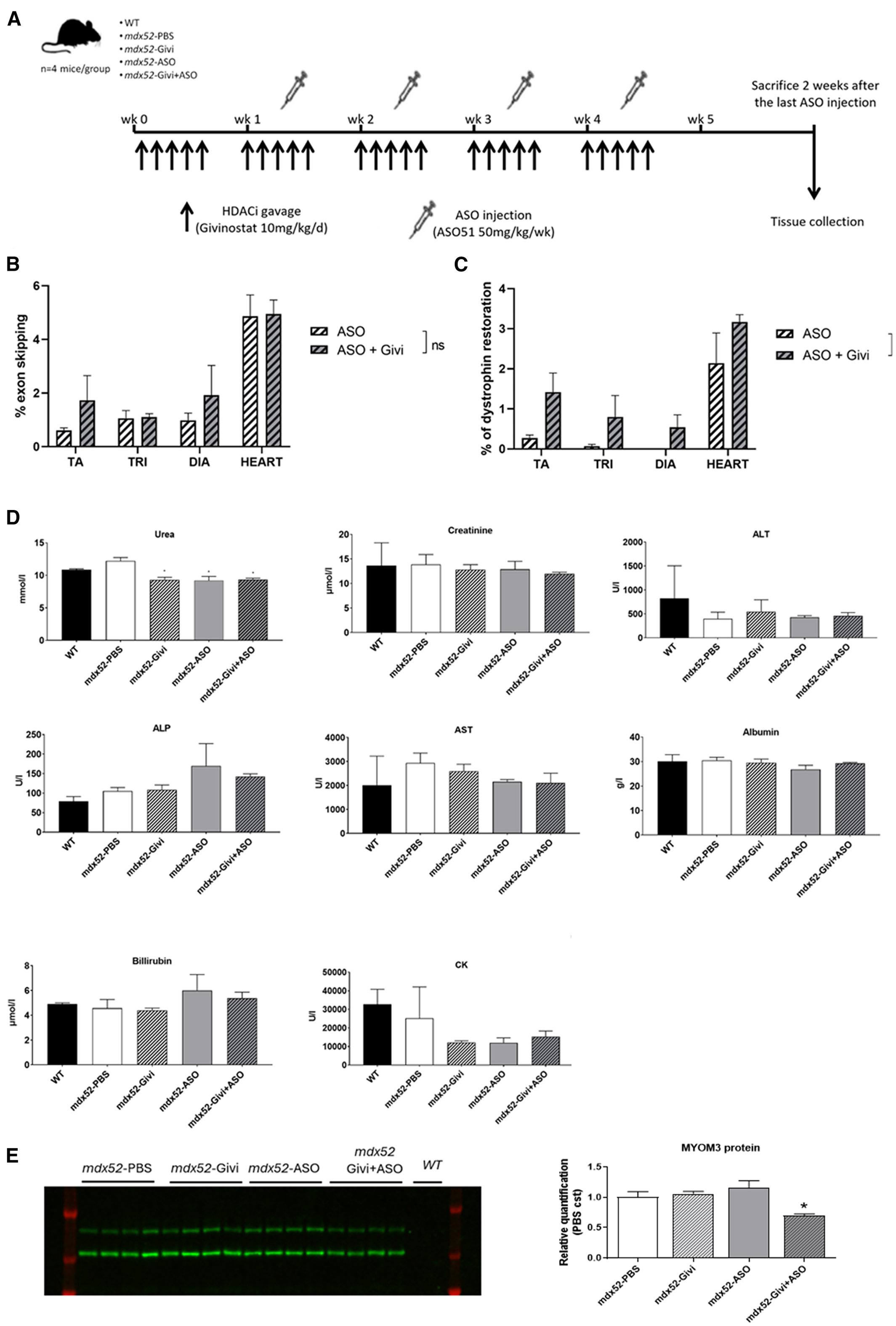

(legend on next page)

To investigate whether the combined Givi + ASO treatment had a beneficial impact on muscle function, we performed a force drop assay on the TA muscle (Figure 6F). Muscles were subjected to 10 consecutive eccentric contractions, with force measured after each contraction. The force drop, calculated as the percentage of force loss relative to the initial contraction, reflects the muscle's resistance to contraction-induced injury and its capacity for recovery. Overall, only mice treated with the Givi + ASO combination showed a significant reduction in force loss after 10 consecutive eccentric contractions (p = 0.0256), compared to *mdx52* control mice treated with PBS. The combined treatment was associated with greater improvements across these readouts compared with ASO treatment alone, suggesting that the combination may provide potential therapeutic benefit. We then assessed the overall muscle damage by measuring the amount of Myomesin 3 in the serum of treated mice (Figure 6G). Only the Givi + ASO treatment significantly reduced the serum level of Myomesin 3 (p = 0.0187).

We next investigated whether the observed increase in dystrophin levels and improved muscle function could be attributed to the restoration of the 5′–3′ *Dmd* transcript imbalance by Givi. To this end, ddPCR analyses targeting various exon junctions along the *Dmd* transcript were performed. No restoration of the 5′–3′ imbalance was detected, indicating that even after 12 weeks of daily Givi treatment, the compound was unable to correct the *Dmd* transcript imbalance in the *mdx52* model (Figures 6H and 6I; Figure S5). We also investigated whether differences in dystrophin restoration could be due to altered oligonucleotide distribution across tissues. Biodistribution analysis revealed similar ASO levels in muscles and off-target organs between Givi + ASO and ASO-only treated mice (Figure S6). These data suggest that major differences in bulk tissue ASO accumulation are unlikely to fully explain the observed increase in dystrophin levels, although more detailed analyses of intracellular ASO distribution and pharmacodynamics would be required to formally exclude effects on ASO delivery or activity. In order to investigate the possible mechanism behind the observed increase in dystrophin levels, we assessed muscle fiber morphology by analyzing fiber size after laminin immunostaining (Figure 7A). As expected, muscle fibers from WT mice appeared well-organized, whereas those from *mdx52* mice exhibited a more disorganized structure, with a higher proportion of small-diameter fibers. Treatment with Givi, ASO, or their combination appeared to partially restore muscle fiber organization. To further quantify this effect, we analyzed fiber size and number in TRIs muscles from WT, *mdx52*, and treated *mdx52* mice (Givi, ASO, or Givi + ASO) (Figure 7B). As anticipated, *mdx52* mice displayed a higher number of small fibers and fewer large fibers compared to WT controls. Treatment with either ASO or Givi reduced the number of small fibers while increasing the number of larger fibers, indicating partial phenotypic improvement. Notably, the combined treatment was associated with a greater reduction in small fibers and an increased proportion of larger fiber populations compared with ASO treatment alone. We focused specifically on fibers ranging from 0 to 500 μm^2 (Figure 7C). All treatments reduced the number of small fibers compared to PBS-treated *mdx52* controls, with statistical significance observed for both Givi (p = 0.0059) and ASO (p = 0.0092). The effect was most pronounced with the combination treatment ($p < 0.0001$). A second analysis focused on larger fibers ranging from 2,000 to 3,000 μm^2 (Figure 7D). Only the Givi + ASO combination led to a significant increase in the number of large fibers compared to *mdx52* controls (p = 0.002), suggesting that neither treatment alone was sufficient to promote the formation of large fibers, but their combination produced a stronger effect on muscle remodeling. Finally, we measured the average cross-sectional area (CSA) of muscle fibers (Figure 7E), which confirmed the known effect of Givi in increasing fiber CSAs in muscles of DMD patients.[32] Givi alone significantly increased mean fiber area compared to untreated *mdx52* mice (p = 0.0351), as did ASO treatment (p = 0.0039). However, the combination treatment resulted in a marked and highly significant increase in fiber area (p = 0.0001), further supporting the additive effect in improving the dystrophic muscle phenotype.

Given the known properties of Givi to reduce fibrosis, we assessed its effect on fibrosis in the treated *mdx52* mice using a Sirius Red staining, which labels collagen fibers in red. As expected, muscle sections from WT mice showed minimal Sirius Red staining, in contrast with *mdx52* muscles. Following treatment with Givi, ASO, or their

Figure 5. Four-week treatment with givinostat and ASO

(A) Schematic overview of the 4-week treatment regimen in *mdx52* mice with ASO and givinostat. (B) ddPCR analysis of exon 51 skipping efficiency in *mdx52* mice (n = 4/group) treated with an exon 51-targeting antisense oligonucleotide (ASO) alone or in combination with givinostat (ASO + Givi). Skipping efficiency was assessed in multiple tissues including tibialis anterior (TA), triceps (Tri), diaphragm (Dia), and heart. Hatched white bars represent ASO-only treatment; hatched gray bars represent ASO + Givi. No statistical difference was detected overall between *mdx52* mice treated with ASO alone and *mdx52* mice treated with the Givi + ASO combination (p = 0.2298 analyzed by two-way ANOVA). (C) Quantification of dystrophin restoration in the same muscle groups using Empiria Studio. Hatched white bars = ASO only; hatched gray bars = ASO + Givi (n = 4/group). A significant difference was found p = 0.0159 between the ASO alone and ASO + Givi using a two-way ANOVA to compare the two groups (*$p < 0.05$), mean difference = 0.8619 ± 0.1389 (SEM), 95% CI: 0.4199 à to 1.304, partial η^2 (effect sizes) = 0.9277, analyzed by t test. The Givi-only treated group is not shown as exon skipping and dystrophin are undetectable without ASO treatment. (D) Serum quantification of general toxicity biomarkers 2 weeks after the last ASO injection: urea, creatinine, ALT, ALP, AST, albumin, bilirubin, and CK (n = 4/group). Significant differences were only observed between the treated (*mdx52*-Givi, ASO, or Givi + ASO) mice compared to saline-treated mice for urea biomarker (p = 0.0383, 0.0258, and 0.0422, respectively) *$p < 0.05$ analyzed by Kruskal-Wallis. (E) Myomesin-3 (MYOM3) levels were also analyzed by western blot and normalized to PBS-treated controls. *$p < 0.05$, **$p < 0.0001$ analyzed by Kruskal-Wallis. On the left, representative western blot of Myomesin-3, a biomarker of muscle damage, in serum samples from *mdx52* mice treated with PBS, ASO alone, or givinostat + ASO combination (n = 4/group). On the right, western blot quantification of MYOM3 levels (n = 4/group). MYOM3 levels were assessed by western blot and normalized to the PBS control group. A significant reduction was observed in the givinostat + ASO group compared to ASO alone (p = 0.0451, analyzed by one-way ANOVA).

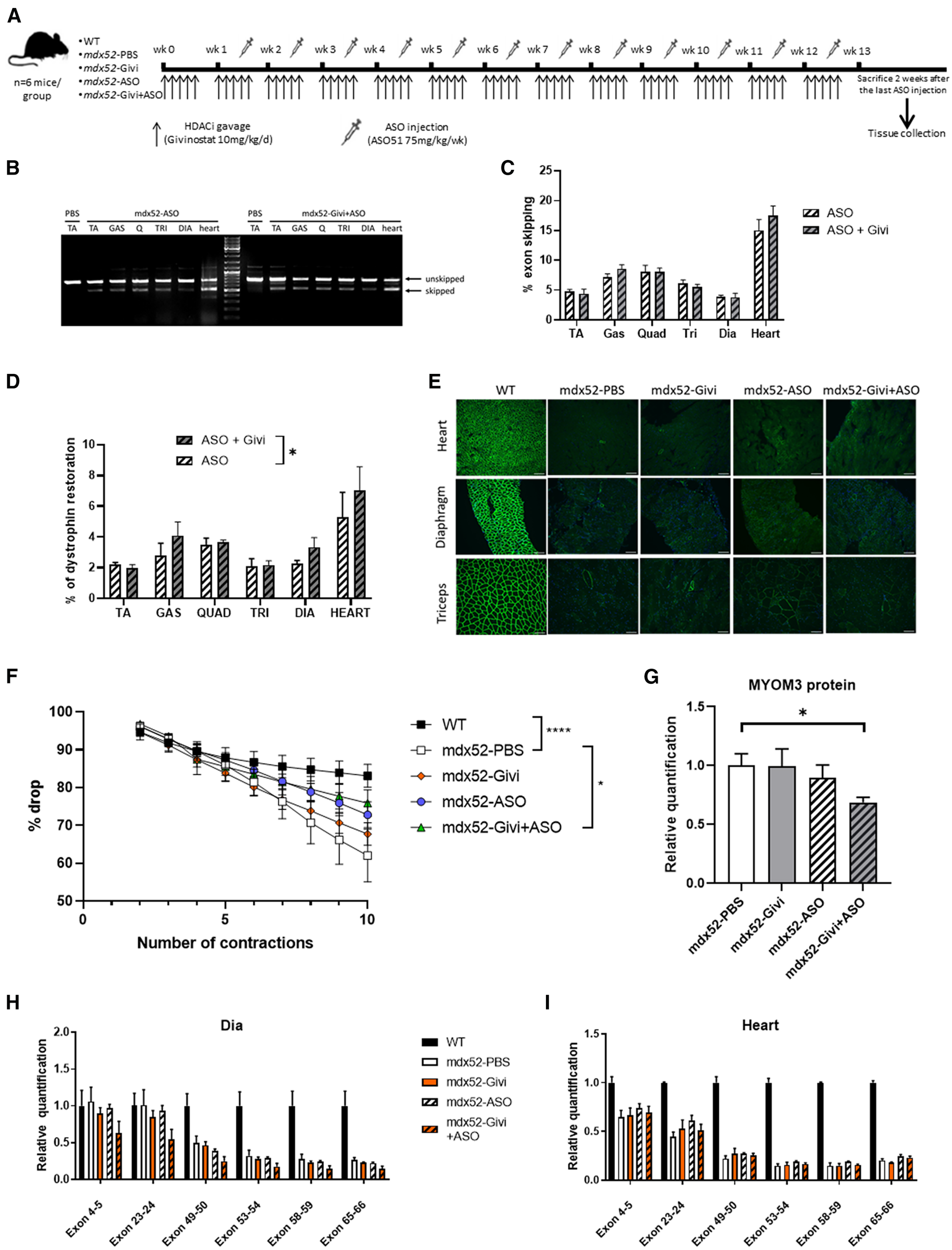

Figure 6. Twelve-week givinostat +ASO long-term treatment study

(A) Schematic representation of treatment regimen schematic for *mdx52* mice receiving ASO and givinostat over 12 weeks. (B) RT-PCR gel electrophoresis of exon skipping in various tissues (TA, GAS, QUAD [quadriceps], TRI, DIA, and heart). Control (TA PBS) shows unskipped band. In treated samples (ASO or Givi + ASO), two bands are observed: upper band = unskipped Δ52 transcript, lower band = exon 51 skipped. (C) ddPCR quantification of skipping efficiency (n = 6/group). White hatched bars = ASO; gray hatched = Givi + ASO. No statistical difference was detected overall between *mdx52* mice treated with ASO alone and *mdx52* mice treated with Givi + ASO (p = 0.4414)

(legend continued on next page)

combination, we quantified muscle fibrosis and found that only the Givi + ASO combination led to a significant reduction in fibrosis compared to the PBS-treated group, as determined by one-way ANOVA (p = 0.0058) (Figure 7G). Moreover, fibrosis levels in the combination group were also significantly lower than in the ASO-only group (t test, p = 0.0088). Of note, we could also observe on the Sirius Red stainings that the high number of centrally nucleated fibers, a hallmark of muscle regeneration observed in control *mdx52* mice appeared reduced in the Givi, ASO, and especially the Givi + ASO treated mice (Figure 7F). Additionally, fibrosis markers were assessed by qPCR and we found that expression of *CTGF* (connective tissue growth factor), *Col1a1* (collagen type 1 alpha 1 chain), *svep1* (Sushi, von Willebrand factor type A, epidermal growth factor (EGF) and pentraxin domain-containing 1) and *Mfap4* (microfibrillar-associated protein 4), four key genes in connective tissue biology such as fibrosis and extracellular matrix synthesis, was significantly reduced in the treated tissues (Figure 7H). Remarkably, only the Givi + ASO combination significantly decreased the expression of *Col1a1* and *Mfap4*.

Given the previously reported effect of Givi on *PGC-1α*,[36] we checked its expression in the treated mice compared to PBS-treated control mice and found a significant upregulation only in the combined Givi + ASO group (*p < 0.05) (Figure 7I). We next assessed PGC-1α protein expression in TRIs muscles of mice treated with Givi, ASO, or the combination of both, using western blot analysis (Figures 7J and 7K). In line with the qPCR results, we observed a significant upregulation of PGC-1α in the Givi + ASO group (p < 0.0001), corresponding to a 4.4-fold increase compared to PBS-treated controls. These findings further support the additive effect of the combined treatment, which likely contributes to the attenuation of the dystrophic phenotype and the encouraging improvements observed in muscle function.

To evaluate whether the combination of HDACi and ASO induced any specific toxicity, we assessed the serum levels of various general biomarkers in mice following the 12-week Givi treatment protocol (Figure S7A). Quantification of creatinine, alkaline phosphatase (ALP), bilirubin, and transaminases (aminotransferase [ALT] and aspartate aminotransferase [AST]) revealed no significant changes in HDACi-treated *mdx* mice compared to saline-treated *mdx* mice. Notably, we observed an improvement in urea and albumin levels in the serum samples of mice treated with Givi + ASO (*p = 0.05, **p = 0.01), suggesting a positive effect on overall metabolic function.

To further investigate the potential renal toxicity of Givi treatment and the combined therapy, several biomarkers were assessed in the urine of the treated mice. Urine samples were collected over 24 h following the final ASO injection, and levels of total protein and albumin were measured and normalized to creatinine. We observed an improvement in normalized total protein levels after treatment with the Givi + ASO combination (Figure S7B). Urinary albumin levels were elevated in *mdx52* mice compared to WT mice, and these levels were significantly reduced after treatment with Givi, ASO, or Givi + ASO (**p < 0.01, ***p < 0.0001).

Overall, these *in vivo* results indicate that while the combination of Givi and ASO does not restore the 5′–3′ *Dmd* transcript imbalance, it leads to a modest but significant increase in dystrophin levels accompanied by consistent improvements in muscle function, histopathology, and fibrosis, without detectable toxicity.

Transcriptomic analysis of Givi effect

In order to identify genes and biological pathways specifically regulated by Givi independently of ASO-mediated exon skipping, we performed RNA sequencing (RNA-seq) analysis of WT and H2K-*mdx52* myotubes treated with either saline PBS or Givi. This exploratory analysis was intended to identify candidate molecular pathways potentially contributing to the *in vivo* effects observed following combined treatment. Principal component analysis (PCA) revealed that PC1 separated *mdx52* from WT samples, while PC2 reflected treatment effects (Figure 8A). A total of 2,568 genes were upregulated and 2,741 downregulated in *mdx52* compared to WT cells (false discovery rate, FDR <0.05). Givi treatment was associated with increased expression of 946 genes and reduced expression of 344 genes (FDR <0.05) (Figures 8B and 8C). The heatmap in Figure 8D shows the 50 genes most significantly associated with genotype (WT vs. *mdx52*) and the 50 most associated with treatment (Givi vs. PBS). Hierarchical clustering clearly separated WT from *mdx52* samples, and within the *mdx52* group, distinguished PBS- from Givi-treated samples, indicating a partial shift of the *mdx52* transcriptome toward a WT-like profile. In Figure 8E, dot plots illustrate normalized expression of six representative genes (*Myh1*, *Apol9a*, *Ddx60*, *Isg15*, *Oasl2*, and *Myog*) significantly associated

analyzed by two-way ANOVA). (D) Dystrophin restoration quantified by Empiria Studio; same groups (n = 6/group) and coding. Data were analyzed using a two-way ANOVA with treatment and muscle type as factors, including the treatment × muscle interaction term. A significant treatment effect was observed between the ASO alone and ASO + Givi groups (p = 0.0168, *p < 0.05). Compared with the ASO + Givi group, the ASO alone group showed lower dystrophin restoration (mean difference = −0.8664 ± 0.3425 SEM, 95% CI: −1.498 to −0.2349). Partial η^2 (effect size) was 0.5614. (E) Immunostaining of dystrophin (green) in transverse sections (heart, diaphragm, and triceps). Nuclei = DAPI (blue). Scale bars, 100 μm. (F) Percentage of force drop over 10 eccentric contractions, expressed relative to initial trial. Values from both TA muscles per mouse are shown. Significant differences were observed between WT and *mdx52*-PBS (p = 0.0004); and between *mdx52*-PBS and *mdx52*-Givi + ASO (p = 0.0150, analyzed by two-way ANOVA), mean difference = 4.480 ± 4.668 (SEM), 95% CI: −5.415 à to 14.38, partial η^2 (effect sizes) = 0.05444. (G) Serum MYOM3 levels (2 weeks after the last ASO injection) quantified by western blot, normalized to PBS group. Hatched = ASO, white = PBS, gray = Givi. Significant differences were observed between *mdx52*-PBS and *mdx52*-Givi+ASO (p = 0.0159, analyzed by one-way ANOVA) (*p < 0.05). ddPCR quantification of *Dmd* expression in (H) diaphragm and (I) heart (n = 6/group). GAPDH-normalized, relative to WT. Black = WT, white = PBS, orange = givinostat, hatched = ASO. No significant differences were detected between the groups using two-way ANOVA.

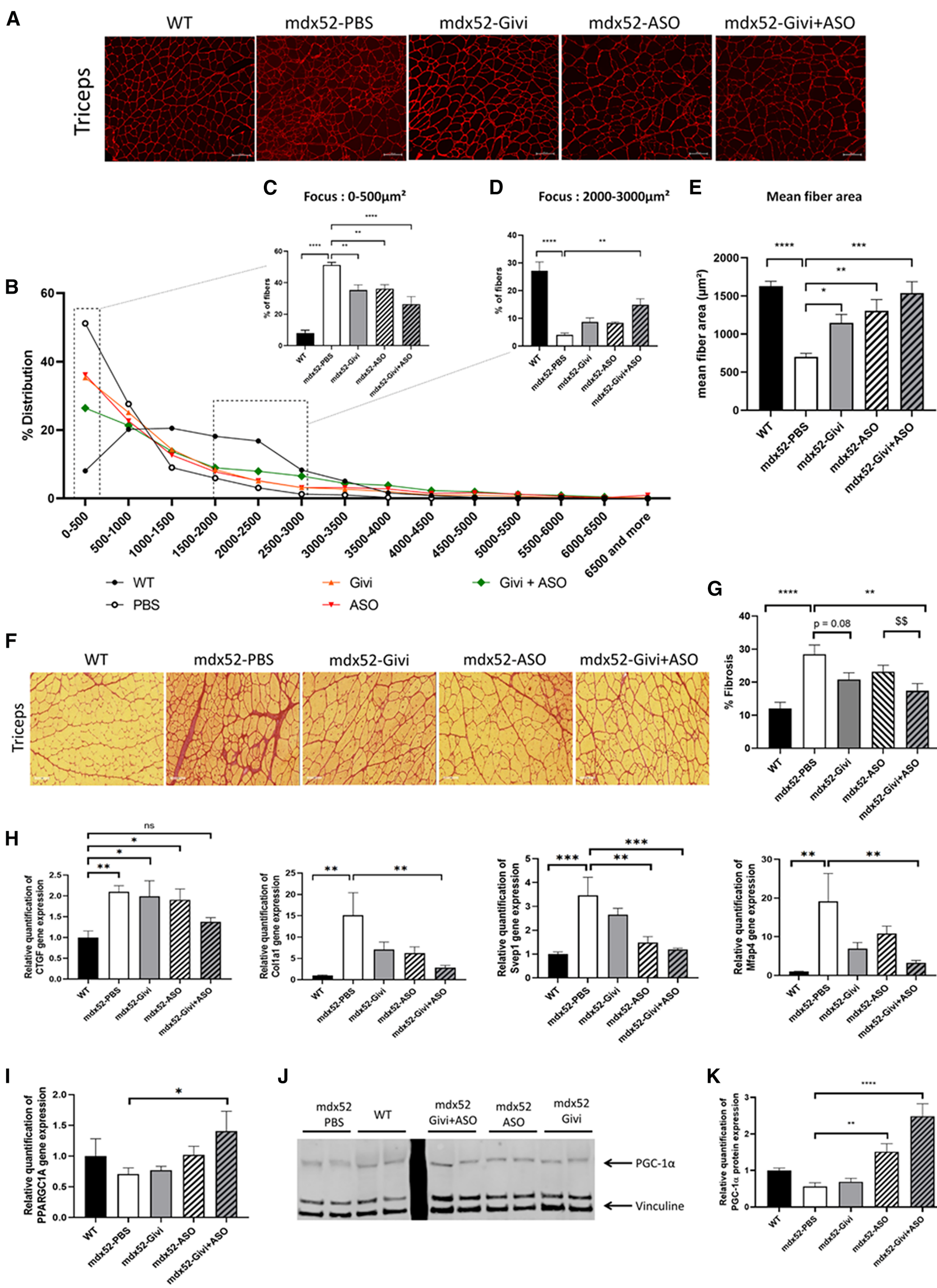

Figure 7. Combined ASO and givinostat treatment improves muscle histopathology

(A) Laminin (red) immunostaining on transverse triceps sections from WT and *mdx52* mice treated with PBS, ASO, Givi, or Givi + ASO ($n = 6$/group). Scale bars, 100 µm. (B) Distribution of fiber sizes across diameter intervals quantified from laminin stainings. (C) Analysis of small fibers (0–500 μm^2) revealed significant differences between WT and *mdx52*-PBS ($p < 0.0001$), as well as between *mdx52*-PBS and treated groups (Givi: $p = 0.0059$; ASO: $p = 0.0092$; Givi + ASO: $p < 0.0001$; one-way ANOVA). (D) Analysis of

(legend continued on next page)

with both genotype and treatment in the RNA-seq dataset. Across all six genes, WT samples (black) displayed distinct expression patterns compared to PBS-treated *mdx52* samples (white), while Givi-treated *mdx52* samples (orange) tended to move toward WT levels, indicating a consistent trend toward transcriptome normalization.

In vivo analysis of TRIs muscles by quantitative reverse-transcription PCR (RT-qPCR) (Figure 8F) confirmed a similar pattern of expression for these six representative genes (*Myh1*, *Apol9a*, *Ddx60*, *Isg15*, *Oasl2*, and *Myog*) in WT and *mdx52* mice treated with PBS and Givi (n = 6/group). Although variability prevented statistical significance for most genes, the expression of all six genes in Givi-treated mice tended to decrease toward WT levels compared to PBS-treated *mdx52* controls, in line with RNA-seq results. The combination of Givi with ASO further reduced expression for most genes, and reached statistical significance for *Myog*, where the Givi + ASO group was significantly lower than PBS-treated *mdx52* controls. Taken together, these transcriptomic analyses suggest that Givi induces a broad but partial remodeling of the dystrophic gene expression program. While these findings should be interpreted with caution given the limited sample size of the RNA-seq experiment, the consistency between *in vitro* RNA-seq data and *in vivo* RT-qPCR validation supports the biological relevance of the observed transcriptional trends.

DISCUSSION

DMD remains a significant challenge in neuromuscular medicine, largely due to the fact that the dystrophin gene is the largest known gene in the human genome, spanning 2.1 million base pairs and comprising 79 exons, which makes it highly susceptible to mutations and difficult to target therapeutically. Although ASO-mediated exon skipping holds therapeutic promise, its effectiveness is limited by suboptimal ASO delivery and the restricted availability of dystrophin pre-mRNA, further complicated by a 5′–3′ transcript imbalance. Increased H3K9me3 levels and enhanced epigenetic modifications at the *Dmd* locus in *mdx* mice suggest that transcriptional repression may result from a more condensed, transcriptionally restrictive chromatin state.[27] However, the *mdx* mouse model, which carries a nonsense mutation in exon 23, does not accurately reflect the spectrum of mutations observed in patients with DMD. In contrast, the *mdx52* model, which harbors a deletion of exon 52, is considered more relevant for studying the disease, as the mutation falls within a known mutational hotspot located in the central region of the *DMD* gene (specifically between exons 45 and 55). This region is particularly prone to exon deletions and represents the most frequently affected segment in DMD patients. Approximately 70% of *DMD*-causing mutations are exon deletions, with the majority clustering within this hotspot.[3,5,6]

In this study, we characterized for the first time the 5′–3′ *DMD* transcript imbalance both *in vitro* and *in vivo* in models carrying a deletion of exon 52. We found that the extent of 5′–3′ imbalance was more pronounced in models lacking exon 52 compared to those harboring a nonsense mutation in exon 23. These findings support the idea that the genomic context, whether sequence-specific elements or local epigenetic features, can significantly influence transcript levels within the same gene. Interestingly, both mutations yield comparable transcript abundance at the 5′ end, indicating that differential degradation by NMD alone cannot explain the more severe downstream loss observed in the *mdx52* model. This suggests that additional mechanisms, such as altered transcriptional elongation or splicing kinetics, may contribute to the imbalance. Notably, our observation that exon skipping tends to modestly increase transcript levels (although without correcting the imbalance) further supports a link between splicing modulation and transcriptional output, echoing recent evidence of genetic compensation mechanisms identified for utrophin.[37] Moreover, emerging human data indicate that deletions in the 3′ region of the *DMD* gene are associated with a more severe transcript imbalance than other mutation types,[38] reinforcing the hypothesis that co-transcriptional processes, rather than purely post-transcriptional degradation, may drive this phenomenon. Taken together, our results indicate that distal mutations, which are common due to the known mutation hotspot, are associated with stronger transcript imbalance and consequently lower *DMD* transcript levels precisely at the region targeted by antisense therapies. This may partly explain the limited efficacy of exon-skipping drugs targeting exons 51 or 53 in humans, compared with generally more robust data obtained preclinically in the classical *mdx* model. Consistent with this, in our study, a 12-week ASO treatment restored

large fibers (2,000–3,000 μm^2) showed significant differences between WT and *mdx52*-PBS (p < 0.0001) and between *mdx52*-PBS and *mdx52*-Givi + ASO (p = 0.002; one-way ANOVA). (E) Mean triceps fiber area analysis demonstrated significant differences between WT and *mdx52*-PBS (p < 0.0001) and between *mdx52*-PBS and treated groups (Givi: p = 0.0351; ASO: p = 0.0039; Givi + ASO: p = 0.0001; one-way ANOVA). (F) Sirius Red staining of triceps sections to assess fibrosis (n = 6/group). Scale bars, 100 μm. (G) Quantification of collagen content from Sirius Red staining (n = 6/group) showed significant differences between WT and *mdx52*-PBS (p < 0.0001) and between *mdx52*-PBS and *mdx52*-Givi + ASO (p = 0.0058; one-way ANOVA). A significant difference was also observed between *mdx52*-ASO and *mdx52*-Givi + ASO ($$$p$ = 0.0335; t test). (H) RT-qPCR analysis of fibrosis-related genes (*CTGF*, *Col1a1*, *Mfap4*, and *Svep1*) in triceps muscles (n = 6/group). Expression levels were normalized to *GAPDH* and expressed relative to WT. For *CTGF*, significant differences were observed between WT and *mdx52*-PBS (p = 0.0061) and between *mdx52*-PBS and treated groups (Givi: p = 0.0140; ASO: p = 0.0269; one-way ANOVA). For *Col1a1*, significant differences were detected between WT and *mdx52*-PBS (p = 0.0016) and between *mdx52*-PBS and *mdx52*-Givi + ASO (p = 0.0081; one-way ANOVA). *Svep1* expression differed significantly between WT and *mdx52*-PBS (p = 0.0002), *mdx52*-PBS and *mdx52*-ASO (p = 0.0032), and *mdx52*-PBS and *mdx52*-Givi + ASO (p = 0.0007; one-way ANOVA). For *Mfap4*, significant differences were observed between WT and *mdx52*-PBS (p = 0.0019) and between *mdx52*-PBS and *mdx52*-Givi + ASO (p = 0.0088; one-way ANOVA). (I) RT-qPCR analysis of *PPARGC1A* expression in triceps muscles (n = 6/group) revealed a significant difference between *mdx52*-PBS and *mdx52*-Givi + ASO (p = 0.049; one-way ANOVA). (J) Representative western blot of PGC-1α protein levels in triceps muscles from WT and treated *mdx52* mice. Vinculin was used as a loading control. (K) Quantification of PGC-1α protein normalized to vinculin and expressed relative to WT using Empiria Studio (n = 6/group). Significant differences were observed between *mdx52*-PBS and *mdx52*-ASO (p = 0.0071) and between *mdx52*-PBS and *mdx52*-Givi + ASO (p < 0.0001; one-way ANOVA). *p < 0.05; **p < 0.01; ***p < 0.001; and ****p < 0.0001.

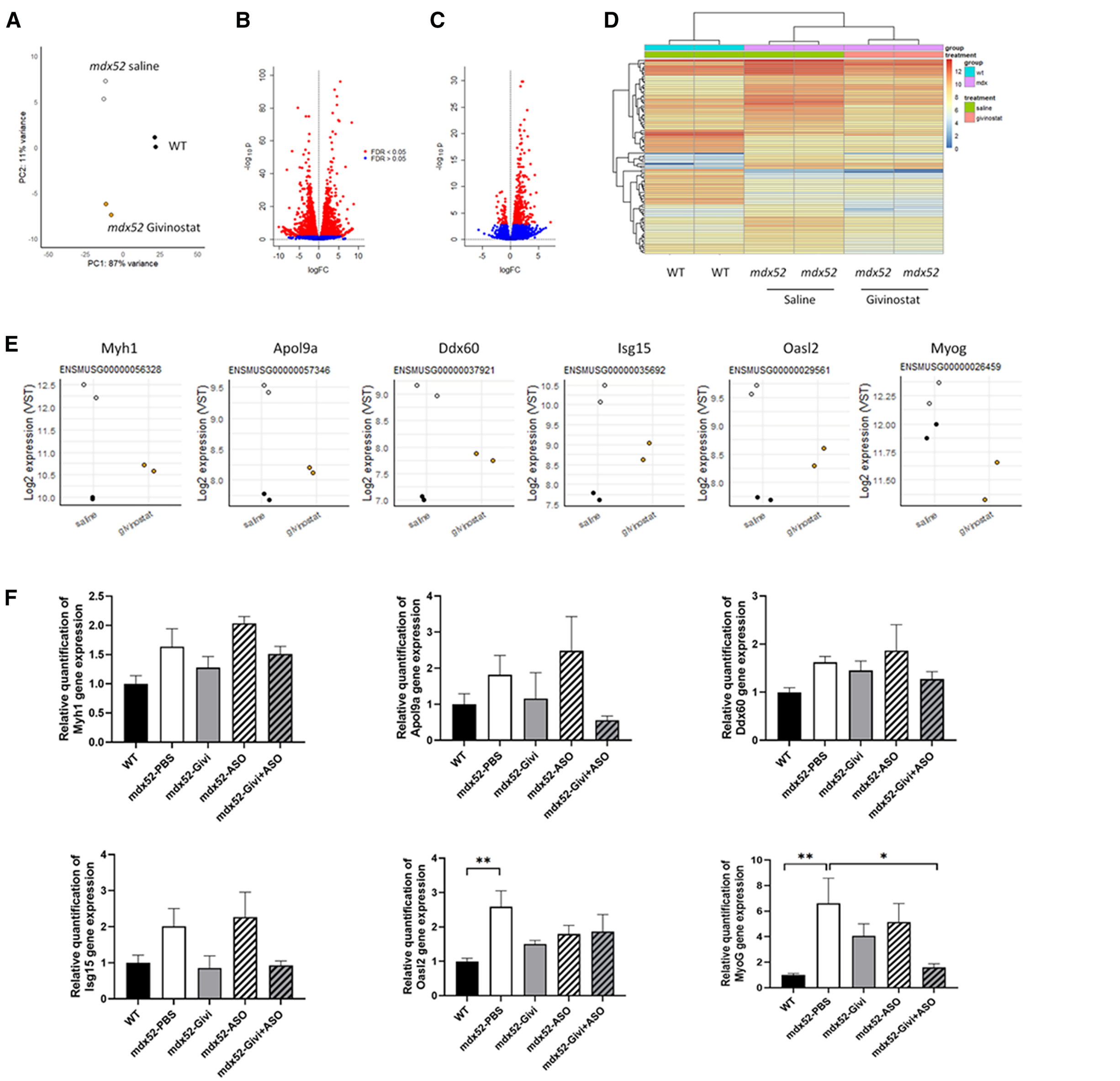

Figure 8. Transcriptomic analysis of givinostat effect

(A) PCA plot showing the separation of WT and H2k *mdx52* samples treated with saline or givinostat ($n = 2$/group). (B and C) Volcano plots ($n = 2$/group) showing fold-change and statistical significance of genes associated with (B) group and (C) treatment. (D) Heatmap showing the expression of the 50 genes most significantly associated with group (*mdx52* vs. WT, $n = 2$/group) and the 50 genes most significantly associated with treatment (givinostat vs. saline, $n = 2$/group). (E) Dot plots showing the expression of six selected genes (*Myh1*, *Apol9a*, *Ddx60*, *Isg15*, *Oasl2*, and *MyoG*) significantly associated with both group and treatment. WT samples are shown in black, saline-treated *mdx52* samples in white, and givinostat-treated *mdx52* samples in orange. (F) RT-qPCR validation of *Myh1*, *Apol9a*, *Ddx60*, *Isg15*, *Oasl2*, and *MyoG* expression in triceps muscle of WT or *mdx52* mice treated with PBS, Givi, ASO, or Givi + ASO ($n = 6$/group). For *Oasl* expression, a significant difference was observed between WT and *mdx52*-PBS ($p = 0.0046$; one-way ANOVA). For *MyoG*, significant differences were detected between WT and *mdx52*-PBS ($p = 0.0051$) and between *mdx52*-PBS and *mdx52*-Givi + ASO ($p = 0.0168$; one-way ANOVA). Data are *GAPDH*-normalized and expressed relative to WT. *$p < 0.05$; **$p < 0.01$.

dystrophin levels to only 2%–6% of WT in *mdx52* mice, whereas a similar approach in *mdx* mice typically yields three times higher protein rescue, around 10%–20%.[34] This pattern aligns with previous reports showing consistently lower dystrophin restoration in *mdx52* compared to *mdx* mice,[39–41] reinforcing the idea that transcript imbalance may negatively impact the therapeutic efficacy of exon-skipping approaches, particularly for more distal mutations. These insights underscore that transcript imbalance remains an underappreciated factor that could limit the effectiveness of genetic therapies for DMD. We believe this phenomenon warrants greater attention

from the research community and therapeutic developers alike, as addressing it will be critical for setting realistic expectations for antisense therapies, optimizing their outcomes, and developing complementary strategies to stabilize or normalize *DMD* transcript levels.[38] Further mechanistic studies are urgently needed to clarify the drivers of transcript imbalance and translate this knowledge into more effective, personalized treatment strategies for patients with DMD.

Given this under-recognized barrier, it is crucial to explore complementary strategies that can mitigate transcript imbalance and increase the availability of targetable pre-mRNA. In this context, HDACi are promising candidates to modulate chromatin structure and enhance transcriptional dynamics. We therefore hypothesized that HDACi could promote histone acetylation, facilitate transcriptional activity, and expand the pool of nascent *DMD* transcripts, ultimately improving the substrate for exon-skipping interventions. Additionally, we aimed to investigate whether combining two clinically approved approaches, Givi and exon 51 skipping, could yield additive or greater-than-additive therapeutic benefits on dystrophin restoration and muscle health.

In our study, we first assessed the efficacy of combining HDACi with ASOs *in vitro* using human myoblasts (KM571Δ52 and their control AB1167HC) and murine myoblasts (H2K*mdx52* and H2KWT). In these studies, human cells responded differently compared to murine cells. This discrepancy may be attributed to species-specific differences in drug uptake, metabolism, and cellular pathways involved in dystrophin expression and exon skipping. Human cells likely have greater sensitivity or more efficient activation of Givi targets, resulting in stronger dystrophin restoration, whereas murine cells may possess distinct regulatory mechanisms or splicing machinery variations that limit treatment efficacy. Understanding these differences is essential for interpreting preclinical results and optimizing therapeutic strategies. Among the tested compounds, only Givi (at a minimum dose of 50 nM) enhanced exon skipping efficiency in human cells, resulting in a 1.6-fold increase in skipped transcripts compared to ASO treatment alone. This increase in exon skipping was also reflected at the protein level, where the 50 nM Givi + ASO condition led to a notable improvement in dystrophin restoration, doubling the amount of dystrophin compared to ASO treatment alone. Yet, this observed increase in dystrophin expression could not be attributed to the correction of the 5′–3′ transcript imbalance. In human cells, treatment with VPA and Givi led to an increase in the absolute levels of *DMD* transcripts, but without restoring transcript balance. In murine cells, no increase in total *Dmd* transcript levels was detected; however, we observed a consistent elevation in transcript expression at the 5′ end of the gene (particularly at the exon 4–5 junction), following treatment with each HDACi at all tested doses. In this study, we used two ASO chemistries: primarily tcDNA-ASO, but also 2′-O-methyl phosphorothioate (2′-O-Me PS) ASO for *in vitro* nascent RNA and RNA-seq experiments. While differences in chemical backbone could influence the magnitude of exon skipping, the directionality and qualitative outcomes (i.e., Givi-enhanced ASO activity) were consistent across both chemistries. This indicates that HDAC inhibition may potentiate ASO therapeutic action regardless of backbone type.

Given that Givi produced the most promising *in vitro* results, we next tested its effect in combination with an exon 51-targeting ASO in *mdx52* mice. In a short-term 4-week study, co-treatment modestly but significantly increased dystrophin restoration compared to ASO alone, despite overall low dystrophin rescue consistent with previous findings in *the mdx52* model. Notably, this early benefit was supported by reduced levels of circulating myomesin-3, suggesting an initial protective effect on muscle integrity. To build on these results, we conducted a 12-week study with a slightly increased ASO dose to improve exon skipping and dystrophin rescue. Although exon-skipping efficiency remained similar between ASO alone and Givi + ASO groups across skeletal and cardiac tissues, dystrophin levels were 1.3-fold higher with the combined treatment. The observed unchanged exon skipping efficacy may be attributed to the fact that exon skipping is expressed as a percentage of total transcripts and does not reflect absolute transcript abundance; one possible explanation is that HDAC inhibition may increase the total pool of *Dmd* transcripts, providing more skipped transcripts for translation and ultimately enhancing dystrophin production, although this hypothesis remains speculative. Additional studies will be required to determine the molecular mechanisms responsible for the increased dystrophin protein observed following the combined treatment. While modest, this slight increase may biologically meaningful given that even small gains in functional dystrophin can have clinical impact.[42–44] Importantly, this translated into measurable functional benefit: only mice receiving the combination therapy showed a significant reduction in force loss during repeated eccentric contractions of the TA muscle.

To assess whether this functional improvement was related to a correction of the 5′–3′ transcript imbalance, we examined *Dmd* transcript profiles. Our data showed that the increase in dystrophin was not accompanied by changes in transcript imbalance. To better understand how Givi enhances dystrophin restoration when combined with ASO therapy, we examined its effects on muscle histopathology. Our results suggest that Givi reduces the proportion of small muscle fibers (0–500 μm^2) and promotes the formation of larger, better-organized fibers, resulting in an overall increase in average fiber CSA. Consistent with its known antifibrotic action, Givi significantly reduced fibrotic deposition in TRIs muscles, as confirmed by Sirius Red staining. Notably, when combined with ASO treatment, this antifibrotic effect was further amplified. At the molecular level, the downregulation of key fibrotic and muscle remodeling genes (*CTGF*, *Col1a1*, *Svep1*, *Mfap4*) mirrored the histological findings, with the combination therapy showing the greatest reduction, supporting an association between combined treatment and improved muscle quality.

In addition to its histological effects, Givi treatment appeared associated with transcriptomic changes in a set of genes that may serve as molecular indicators of its biological activity. Among the six representative genes identified in our RNA-seq analysis (*Myh1*, *Apol9a*, *Ddx60*,

Isg15, *Oasl2*, *Myog*), all showed a trend toward reduced expression in Givi-treated *mdx52* mice compared to PBS controls, with the combination therapy (Givi + ASO) further decreasing expression for some of them. From a functional standpoint, *Myh1* encodes a fast-twitch myosin heavy chain predominantly expressed in type 2X fibers, known to be preferentially damaged in DMD. Fast-twitch fiber-specific biomarkers such as troponin I type 2 (TNNI2) are increasingly used in clinical trials to monitor muscle damage more specifically than creatine kinase.[45] A reduction in *Myh1* expression after Givi treatment may therefore reflect a fiber-type shift from fast toward slower-twitch fibers, a phenomenon reported with other therapeutic agents such as sevasemten in Becker muscular dystrophy.[46] Such a shift could reduce susceptibility to contraction-induced damage, lowering the need for regeneration, as reflected by decreased *Myog* expression. Future spatial transcriptomics or immunohistochemical analyses could confirm whether this transcriptional signature corresponds to a reduced prevalence of Myh1-positive fibers or the emergence of hybrid fibers co-expressing Myh1 and other myosin isoforms. Despite the limited replication, these transcriptomic results collectively suggest that Givi partially normalizes dystrophic gene expression, reducing fibrosis- and inflammation-related transcripts and restoring *MyoG* expression to near WT levels. This overall transcriptional rebalancing is consistent with the structural and histological improvements observed *in vivo*, and future immunohistochemistry analysis of MyoG (with or without Pax7 co-expression) will help determine whether active regeneration contributes to the improved muscle fiber phenotype. The other three genes (*Ddx60*, *Isg15*, and *Oasl2*) are interferon-stimulated genes (ISGs) implicated in innate immune responses. *Ddx60* and *Oasl2* also participate in RNA sensing or processing, functions that may be relevant in the dystrophic muscle context, where chronic inflammation and aberrant RNA metabolism are common features. The coordinated downregulation of these ISGs following Givi treatment could therefore indicate a broader anti-inflammatory or immunomodulatory effect, complementing the histological evidence of reduced fibrosis and improved fiber morphology. Overall, these six genes, particularly *Myh1* and the ISGs, could serve as useful molecular markers to assess Givi activity in preclinical and clinical settings. Their modulation by treatment suggests that beyond improving muscle structure, Givi may influence fiber-type composition and inflammatory signaling, potentially contributing to improved muscle preservation in combination with exon-skipping therapies in DMD.

One limitation of this proof-of-concept study is the absence of a dedicated vehicle-only group matching the exact composition of the givinostat vehicle (5% DMSO, 40% PEG300, 5% Tween-80). While these vehicle components have been widely used at similar concentrations without reported effects on muscle histology or nucleic acid delivery,[31,47] the lack of an internal matched vehicle control limits the extent to which the observed effects can be attributed specifically to Givi. Future studies should therefore include a matched vehicle control to further validate these observations.

Although Givi appears to modestly enhance dystrophin restoration, it did not improve ASO-mediated exon skipping or correct the 5′→3′ transcript imbalance in murine cells, indicating that additional mechanisms likely contribute to the observed treatment effects. This observation suggests that factors such as chromatin accessibility, transcriptional elongation, or local transcript availability may play a role. Future studies investigating histone modifications in the *mdx52* model (e.g., via chromatin immunoprecipitation, ChIP) and employing spatial transcriptomics could help clarify which transcripts are regulated in the specific cells where dystrophin is expressed, providing mechanistic insight into the observed improvement in dystrophin levels.

Given the low toxicity profile of the 12-week Givi + ASO treatment (reflected by the absence of hepatic or renal toxicity and modest improvements in blood urea and proteinuria markers) and the overall improvement in the dystrophic phenotype, the combined use of Givi and exon 51-targeted ASO may represent a viable and translatable therapeutic strategy for DMD patients. Notably, both types of compounds have already been approved or are advancing through regulatory pathways (Givi and some phosphorodiamidate morpholino oligomer (PMO)-ASO are FDA approved, while tcDNA-ASOs are currently in phase 2 clinical trials). The ability to simultaneously address the primary cause of the disease (lack of dystrophin) and its secondary pathological consequences (fibrosis, inflammation, and impaired energy metabolism) underscores the therapeutic potential of this combination to improve the global phenotype in DMD patients.

In summary, our findings demonstrate that combining Givi with ASO treatment modestly but significantly enhances dystrophin restoration and improves muscle pathology and function in the clinically relevant *mdx52* model, although the precise molecular mechanisms underlying these effects remain to be fully determined. The observed benefits appear associated with improved muscle fiber quality and reduced fibrosis rather than changes in transcript abundance alone, potentially reflecting broader effects of Givi on muscle remodeling. Overall, these results support the rationale for exploring approved drug combinations to help overcome limitations of current exon-skipping therapies and strengthen the case for developing combinatorial strategies to enhance muscle preservation in DMD.

MATERIALS AND METHODS

ASOs

The tcDNA-ASO targeting exon 51 of the *DMD* pre-mRNA was synthesized by SQY Therapeutics (Montigny-le-Bretonneux, France). Palmitic acid was conjugated to the 5′ end of the tcDNA-PO (phosphodiester) via a C6-amino linker and a phosphorothioate bond, as described previously.[31] A tcDNA-ASO targeting exon 51 was used in all experiments except for the nascent RNA and RNA-seq experiments, in which 2′-O-Me PS ASOs were used.

Cell culture, differentiation, HDACi treatment, and oligonucleotide transfection

Immortalized HC and DMD patient cell lines were kindly provided by Prof. Vincent Mouly (Institute of Myology, Paris).[48,49] Human

(KM571Δ52, AB1167HC) and murine myoblasts (H2K *mdx52*, H2KWT) were cultured in 6-well plates at a density of 300,000 cells per well in appropriate growth media. For human cells, the growth medium consisted of a 1:1 mixture of F-10 Nutrient Mix (1X) supplemented with GlutaMAX and Promocell Skeletal Muscle Cell Growth Medium, supplemented with 10% fetal bovine serum (FBS) and 1% penicillin/streptomycin. Murine cells were cultured in DMEM GlutaMAX (4,500 mg/L glucose, 4 mM L-glutamine, 110 mg/L sodium pyruvate) supplemented with 2% Gibco L-glutamine, 2% chicken embryo extract, 0.4% bovine gelatin, and 1% penicillin/streptomycin. Human cells were incubated at 37°C with 5% CO_2, while murine cells were maintained at 33°C in a humidified atmosphere with 10% CO_2. Twenty-four hours post-seeding, cells were treated with various concentrations of HDACis. Givi, and TSA were tested at 10, 50, 150, and 300 nM; VPA was applied at 0.5, 1, 3, and 10 mM. Simultaneously, cells were induced to differentiate by switching to differentiation media. For human cells, differentiation medium consisted of DMEM GlutaMAX supplemented with 2% horse serum and 1% penicillin/streptomycin. For murine cells, differentiation was performed using DMEM GlutaMAX supplemented with 5% horse serum, 2% L-glutamine, and 1% penicillin/streptomycin. Both cell types were incubated at 37°C with 5% CO_2 during differentiation. Twenty-four hours after the initiation of differentiation, cells were transfected with an ASO targeting exon 51 of the *DMD* pre-mRNA. Transfection was performed using Lipofectamine LTX (Thermo Fisher Scientific) following the manufacturer's protocol, with a final ASO concentration of 100 nM per well. Cells were incubated with the oligonucleotide for three consecutive days without medium replacement. For transcript-level analyses, cells were harvested on day 5 of differentiation using TRIzol reagent (Thermo Fisher Scientific), following the manufacturer's instructions. For protein analysis, cells were collected on day 6 of differentiation. Total protein lysates were prepared in radioimmunoprecipitation assay (RIPA) buffer supplemented with protease inhibitors and 5% SDS. Protein concentrations were determined using the BCA (bicinchoninic acid) Protein Assay Kit (Thermo Fisher Scientific).

All experimental conditions were conducted in three to six biological replicates, depending on the experiment. Untreated and vehicle-treated controls were included in all assays.

Analysis of nascent RNA

KM155 (HC) and KM1328 (carrying a deletion of exon 52) cells were seeded on 0.5% gelatin-coated Petri dishes and they were left growing for at 37°C with 5% CO_2. After 2 days, cells were differentiated into myotubes by switching to fusion medium for 3 days. KM155 cells were then treated with saline solution, while KM1328 were treated with either saline or Givi at 50 nM concentration. One day after treatment with either saline or Givi, cells were transfected with a 2′-*O*-methyl phosphorothioate ASO targeting exon 51 using Lipofectamine 2000 at a concentration of 100 nM. The ASO sequence was CCUCUGUGAUUUUAUAACUUGAU. Eight hours after transfection, bromouridine was added to the medium with a concentration of 1 mM for 30 min to allow sufficient labeling of nascent RNA. After the labeling, cells were washed twice with ice-cold PBS and cells were scraped after adding 1 mL TRIzol to the dish. Cells were then frozen in −80°C until further use. The experiment was performed in duplicate. After RNA purification, bromouridine carrying transcripts were pulled-down as previously described[27] using an anti-BrdU antibody coupled to anti-mouse IgG magnetic Dynabeads. Samples were then prepared for sequencing using a stranded RNA-seq protocol followed by adaptor ligation with barcoded adaptors using the KAPA HyperPrep Kit. Individual libraries were pooled and sequenced using Illumina NovaSeq sequencing, Paired-End, 150 bp.

Data were aligned with STAR (Spliced Transcripts Alignment to a Reference) and coverage was visualized using the *CoverageView* R package for .bam files using rpm normalization. Reads were assigned to 20 bins representing 5% of the gene body for each locus. Differences in levels of nascent transcripts were assessed using a linear model accounting for gene length (in percentage), category (KM155 vs. KM1328) and treatment (saline, ASO, Givi, and the combination treatment).

Animal experiments and quantification procedures

All animal procedures were conducted in accordance with national and European guidelines and were authorized by the French Ministry of Higher Education and Research (authorization APAFiS #6518). Exon 52-deleted X-chromosome-linked muscular dystrophy model mice (*mdx52* mice) were produced by replacement of exon 52 of the *Dmd* gene with a neomycin resistance cassette, thereby eliminating expression of Dp427, Dp260, and Dp140 dystrophin isoforms but preserving expression of isoforms Dp116 and Dp71.[35] Breeders were generously provided by Dr. Jun Tanihata and Dr. Shin'ichi Takeda (National Center of Neurology and Psychiatry, Tokyo, Japan). The *mdx52* mice were bred in the animal facility at Plateforme 2Care, UFR des Sciences de la Santé, Université de Versailles Saint-Quentin, and maintained on a standard 12-h light/dark cycle with unrestricted access to food and water. Mice were weaned at 4–5 weeks of age, with 2–5 mice housed per cage. Groups of 6–7-week-old *mdx*52 mice received i.v. injections of either 50 or 75 mg/kg/week of the tcDNA-ASO (administered weekly under general anesthesia with 2% isoflurane) in combination with Givi (CliniSciences, dissolved in a solution of 5% DMSO, 40% PEG300, 5% Tween80, and 50% ddH2O according to the manufacturer's instructions, at a final dose of 10 mg/kg/day) or saline for 12 weeks (n = 6 mice per group).

Givi was administered by oral gavage 5 times per week. Age-matched *mdx*52 mice receiving an equivalent volume of sterile saline were used as controls, with C57BL/6 WT mice included as additional controls. Although a dedicated vehicle-only group was not included in this study, the concentrations and dosing volume used are below levels reported to influence muscle physiology or ASO uptake in mice.[31,47] All animals that completed the treatment were included in analyses unless tissue damage occurred during dissection.

Humane endpoints followed institutional ethical standards, including daily monitoring of body weight, posture, and distress. Animals were euthanized 2 weeks after the final ASO injection, and muscle and brain tissues were harvested. The tissues were snap-frozen in liquid nitrogen-cooled isopentane and stored at −80°C for subsequent analysis.

Animals were randomly allocated across experimental groups while balancing litter origin, birth date, and body weight in order to minimize potential litter-related biases. Ear markings were used solely for animal identification and treatment follow-up. Although complete blinding during *in vivo* handling procedures was not always feasible because animals remained individually identifiable throughout treatment administration and monitoring, several measures were implemented to minimize observer bias during downstream analyses.

Force measurements were performed independently by a blinded external operator. Fiber size analysis was conducted using the Imaris machine-learning segmentation algorithm with identical parameters applied across all images. Fibrosis quantification was performed automatically using a macro-based analysis of Sirius Red signal intensity. Western blot quantifications were performed using Empiria Studio with standardized analysis procedures applied identically across all samples, while ddPCR quantifications were based on absolute droplet counts. Exclusion criteria were limited to technical issues during sample preparation. Group sizes were selected based on previously published *mdx* and *mdx52* studies demonstrating sufficient statistical power.

Serum and urine analysis

Blood samples were collected 2 weeks after the last ASO injection at the conclusion of the treatment for MYOM-3 and biochemical analyses. Serum levels of alanine ALT, AST, ALP, bilirubin, creatinine, urea, and albumin were measured by the pathology laboratory at the Mary Lyon Center, Medical Research Council, Harwell, Oxfordshire, UK. Urine samples were collected 1 week after the last ASO injection over a 24-h period using metabolic cages, and the concentrations of creatinine and total protein were assessed according to established protocols.[28]

Exon skipping analysis

Total RNA was isolated from snap-frozen muscle tissues using TRIzol reagent according to the manufacturer's instructions (Thermo Fisher Scientific, USA). Quantification of exon 51 skipping was performed by real-time quantitative ddPCR using 100 ng of cDNA and TaqMan assays targeting the exon 50–51 junction (mex50–51 for murine samples or hex50–51 for human samples) and the exon 50–53 junction (mex50–53 or hex50–53). Exon 51 skipping was calculated as the percentage of total *Dmd* transcripts, based on the sum of copy numbers for exon 50–51 and exon 50–53 junctions. For visualization of exon 51 skipping, RT-PCR was carried out using 50 ng of cDNA. In human samples, primers targeted exon 49 (hex49F, forward) and exon 53 (hex53R, reverse), while in murine samples, primers targeted exon 50 (mex50F, forward) and exon 55 (mex55R, reverse) (Table S2). PCR products were analyzed by gel electrophoresis.

5′–3′ DMD transcript imbalance analysis

Dmd transcript quantification by ddPCR at various exon-exon junctions in human samples was performed using 100 ng of cDNA, with probes targeting hex4–5, hex23–24, hex49–50, hex53–54, hex58–59, and hex65–66 junctions of the *DMD* gene, as well as *hGAPDH* (Integrated DNA Technologies) (Table S2). Similarly, for murine samples, quantification was carried out with 100 ng of cDNA using probes targeting mex4-5, mex23-24, mex49-50, mex53-54, mex58-59, and mex65-66 junctions of the *Dmd* gene, along with *mGAPDH* (Integrated DNA Technologies) (Table S2).

Quantification of fibrosis and other biomarkers was conducted by qPCR using 50 ng of TRIs-derived cDNA from mice, employing the iTaq Universal SYBR Green Supermix (Bio-Rad). Primer and probe sequences for all qPCR assays are listed in Table S2.

Western blot protocol for detection of dystrophin, PGC-1α and Myomesin-3

Protein lysates were prepared from muscle sections obtained during cryosectioning using the Precellys 24 (Bertin Instruments, France) in RIPA buffer (Thermo Fisher Scientific, USA) supplemented with SDS powder (5% final concentration, Bio-Rad, France) and a protease inhibitor cocktail (Thermo Fisher Scientific, USA). The protein extracts were denatured by heating at 100°C for 3 min, followed by centrifugation at 13,000 rpm for 10 min at 10°C. The supernatant was collected, and the total protein concentration was determined using the BCA Protein Assay Kit (Thermo Fisher Scientific, USA). For electrophoresis, 25 μg of protein was loaded onto NuPAGE 3%–8% Tris-Acetate Protein Gels (Invitrogen) according to the manufacturer's guidelines.

Dystrophin was detected using the iBind Flex Western Device (Fisher Scientific). Membranes were probed with the primary monoclonal antibody NCL-DYS1 (Novocastra, Newcastle, UK, 1:200 dilution) and hVin-1 (Sigma, 1:4,000 dilution) for vinculin. After incubation with a goat anti-mouse secondary antibody (IRDye 800CW Goat anti-mouse IgG, Li-Cor, Germany, 1:2,000 dilution), protein bands were visualized using the Odyssey CLx system (Li-Cor, Germany). Dystrophin expression was quantified using Empiria Studio software (Li-Cor, Germany), with normalization against a standard curve generated from pooled lysates of C57BL10 (WT and *mdx* control tissues. Each gel contained a 5-point calibration curve (0%–20% of WT dystrophin) prepared by mixing WT and *mdx52*-PBS lysates at defined ratios (0%, 5%, 10%, 15%, and 20%). Calibration standards were muscle-matched to the analyzed samples. The assay was linear across the tested range (with R^2 values ranging from 0.8649 to 1 depending of the standard curve).

For PGC-1α detection, membranes were incubated with the primary rabbit monoclonal antibody PGC-1α (NBP1-04676, Bio-Techne, 1:1,000 dilution) and the primary mouse monoclonal antibody

hVin-1 (Sigma, 1:4,000 dilution) to detect vinculin. After primary antibody incubation, membranes were incubated with secondary antibodies: Goat anti-Rabbit IgG (IRDye 680CW, Li-Cor, Germany, 1:2,000 dilution) for PGC-1α and Goat anti-Mouse IgG (IRDye 800CW, Li-Cor, Germany, 1:2,000 dilution) for vinculin. Protein bands were visualized using the Odyssey CLx system (Li-Cor, Germany). For quantification, the signal intensities for PGC-1α were normalized to vinculin expression, which served as a housekeeping protein (loading control). Data were expressed relative to the WT samples, which were set to 1.

For Myomesin-3 detection,[50] mouse serum was diluted 1:20 and loaded onto 3%–8% Criterion XT Tris-Acetate Protein Gels (Bio-Rad, France). The membrane was probed with a primary rabbit polyclonal antibody against MYOM3 (Proteintech, Manchester, UK), followed by incubation with a secondary goat anti-rabbit antibody (IRDye 800CW Goat anti-rabbit IgG, Li-Cor, Germany). Bands were visualized using the Odyssey Imaging System (Biosciences, Lincoln, USA). The intensity of the signals in treated samples was quantified and normalized to the PBS control group using Image Studio software (Li-Cor, Germany).

Immunohistochemistry analysis of dystrophin and laminin

Cryosections of muscle tissues (10 μm thickness for dystrophin and 8 μm thickness for laminin) were prepared from frozen tissue samples and stored at −80°C until use. Upon preparation for staining, the slides were removed from storage and allowed to dry at room temperature (RT) for 20–30 min. Tissue areas of interest were circled using a hydrophobic barrier pen (Super PAP Pen, Fisher Scientific #10120573) to retain reagents during incubation. Sections were rinsed twice in 1× PBS (0.1 M; Gibco DPBS (Dulbecco's Phosphate Buffered Saline), no calcium, no magnesium; Fisher Scientific #12037539) for 5 min each under gentle agitation. For blocking, slides were incubated for 1 h at RT in a humidified chamber with ∼200 μL of a blocking buffer containing 2% bovine serum albumin (BSA; Fisher Scientific #11413164) and 2% FBS (Fisher Scientific #11570636) diluted in 1× PBS. After blocking, sections were incubated overnight at 4°C with the primary antibody. For dystrophin analysis, a rabbit polyclonal anti-dystrophin antibody (1:500 dilution; Thermo Fisher Scientific RB-9024-P, Fisher #12603347) was used, while for laminin, a rabbit polyclonal anti-laminin antibody (1:200 dilution; Sigma-Aldrich L9393) was applied. Both primary antibodies were prepared in blocking buffer and applied in a humidified chamber.

The following day, slides were washed twice in 1× PBS for 5 min each. For secondary antibody incubation, a goat anti-rabbit IgG (H + L) F(ab')$_2$ fragment conjugated to Alexa Fluor 488 (1:500 dilution; Fisher Scientific #10082502) was used for dystrophin, and an anti-rabbit Alexa Fluor 488 (1:1,000 dilution; Fisher Scientific) was applied for laminin. The secondary antibodies were incubated for 1 h at RT, in the dark and in a humidified chamber. After secondary antibody incubation, the slides were washed three times with 1× PBS for 5 min each. For nuclear counterstaining, DAPI (1000X stock, diluted to 1X) was applied to each section for 15 min at RT, protected from light. Sections were then washed three times with 1× PBS for 5 min each in the dark. Finally, the sections were briefly air-dried without allowing them to dry completely and mounted using DAPI Fluoromount-G (Clinisciences #0100-20) for dystrophin or Fluoromount-G for laminin. Coverslips (24 × 50 mm; Fisher Scientific #11911998) were carefully applied. The slides were left to dry at RT in the dark, then stored at 4°C, protected from light. Fluorescence images for both dystrophin and laminin were acquired under consistent conditions, ensuring the same anatomical regions and exposure settings, using a Zeiss Axio Imager microscope coupled with an Orkan camera (Hamamatsu), and controlled through AxioVision software. Quantitative image analysis was performed using automated segmentation algorithms (Imaris software with identical parameters applied across all samples to minimize observer bias).

Fibrosis analysis

Frozen tissue sections were air-dried at RT for 1 h prior to fixation. Fixation was performed by immersing the slides in xylene for 10 min. Sections were then rehydrated through a graded ethanol series with 15-s incubations in 100%, 70%, and 35% ethanol, followed by a 30-s rinse in Milli-Q water. Staining was carried out using Picro Sirius Red solution (Abcam, ab246832) for 45 min at RT. Slides were then washed twice in 0.5% acetic acid for 1 min each, followed by a brief 15-s rinse in Milli-Q water. Dehydration was performed by sequential immersion in 70%, 95%, and 100% ethanol for 30 s each. Sections were cleared in xylene twice for 2 min each and mounted using a non-aqueous mounting medium (e.g., VectaMount Permanent Mounting Medium). Stained sections were imaged using the LEICA ST5020 automated slide stainer, and quantitative analysis was performed using Aperio ImageScope software (macro-based intensity analysis with identical parameters applied across all samples to minimize bias).

Susceptibility to contraction-induced loss of function

Maximal muscle force before and after eccentric contractions was evaluated by measuring the *in situ* contraction of the TA muscle in response to sciatic nerve stimulation, following established protocols.[31,51] Force-drop assays were performed by an independent blinded operator. To assess the vulnerability of *mdx52* mice to contraction-induced damage, the immediate reduction in isometric force after eccentric contractions was recorded. A series of ten eccentric contractions was applied, with 45-s intervals between each contraction. Maximal isometric force was measured after every contraction and expressed as a percentage of the initial peak force. Upon completion of the functional assessment, animals were euthanized via cervical dislocation.

RNA-seq experiment

To study gene expression of cells exposed to Givi, total RNA of WT and *H2K-mdx52* was purified and used for RNA-seq. We used the Watchmaker mRNA Library Prep Kit. Sequencing was performed on Illumina NovaSeq aiming to obtain 5 million reads per sample. Sequencing data were analyzed using the BIOPET

Gentrap in-house pipeline (https://biopet-docs.readthedocs.io/en/stable/pipelines/gentrap/). Quality control was performed using FastQC and MultiQC. Reads were aligned to mouse reference genome GRCm38 using STAR aligner; an average of ~90% of reads was uniquely assigned to known genes. Analysis of count data was performed using the Deseq2 package to compare expression across groups and treatment. Multiple testing correction was done using Benjamini-Hochberg FDR.

Statistical analysis

All *in vivo* data were processed and analyzed using GraphPad Prism v.10 (GraphPad Software, San Diego, CA, USA). Results are expressed as mean values ± standard error of the mean (SEM), with "*n*" indicating the number of animals per group. Statistical comparisons between groups were performed using one-way or two-way analysis of variance (ANOVA), incorporating repeated measures where appropriate (e.g., comparisons across distinct exon junctions or muscle types). Post hoc analyses, including Dunnett's or Sidak's multiple comparisons tests, were applied when relevant. To evaluate the global effects of treatment across tissues or exon junctions, a two-way ANOVA was employed, with the associated *p* value for treatment effects specified in the figure legends. For datasets not meeting the assumptions of normality, as determined by the Shapiro-Wilk test, group differences were assessed using the non-parametric Kruskal-Wallis test.

DATA AND CODE AVAILABILITY

The primary data for this study are available from the authors upon request.

ACKNOWLEDGMENTS

We thank Dr. Vincent Mouly (Institute of Myology, Paris, France) for providing the myogenic cell lines used in the study. We thank Dr. Katsuki Motoya and Prof. Sasaoka Toshikuni (Department of Comparative and Experimental Medicine/Brain Research Institute; Niigata University, Japan), and Dr. Jun Tanihata and Dr. Shin'ichi Takeda (National Center of Neurology and Psychiatry, Tokyo, Japan) for providing the *mdx52* mouse breeders. We would like to thank the personnel of the plateforme 2CARE for taking care of the animals used in this work. The animal study protocol was approved by the Ethics Committee CNREEA47 and the French government (ministère de l'enseignement supérieur et de la recherche, authorization APAFiS #6518).

This work was supported by the Association Française contre les Myopathies (AFM grant number 24114), the Institut National de la Santé et de la Recherche Médicale, the Association Monegasque contre les myopathies (AMM), the Paris Ile-de-France Region and the Fondation UVSQ. X.P. is the recipient of a doctoral fellowship from the AFM (#24563).

AUTHOR CONTRIBUTIONS

Conceptualization, X.P., P.S., and A.G.; methodology, X.P., C.G., A.M., M.D., O.L.C, S.d.H., P.S., L.G., and A.G.; formal analysis, X.P., C.G., S.d.H., P.S., and A.G.; writing – original draft preparation, X.P. and A.G.; writing – review and editing, P.S. and A.G.; supervision, P.S. and A.G.; funding acquisition, A.G. All authors have read and agreed to the published version of the manuscript.

DECLARATION OF INTERESTS

L.G. is co-founder of SQY Therapeutics, which produces tcDNA oligomers.

SUPPLEMENTAL INFORMATION

Supplemental information can be found online at https://doi.org/10.1016/j.omtn.2026.103047.

REFERENCES

1. Bushby, K., Bourke, J., Bullock, R., Eagle, M., Gibson, M., and Quinby, J. (2005). The multidisciplinary management of Duchenne muscular dystrophy. Curr. Paediatr. *15*, 292–300. https://doi.org/10.1016/j.cupe.2005.04.001.
2. Hoffman, E.P., Brown, R.H., and Kunkel, L.M. (1987). Dystrophin: the protein product of the Duchenne muscular dystrophy locus. Cell *51*, 919–928. https://doi.org/10.1016/0092-8674(87)90579-4.
3. Koenig, M., Beggs, A.H., Moyer, M., Scherpf, S., Heindrich, K., Bettecken, T., Meng, G., Müller, C.R., Lindlöf, M., Kaariainen, H., et al. (1989). The molecular basis for Duchenne versus Becker muscular dystrophy: Correlation of severity with type of deletion. Am. J. Hum. Genet. *45*, 498–506.
4. Aartsma-Rus, A., Van Deutekom, J.C.T., Fokkema, I.F., Van Ommen, G.-J.B., and Den Dunnen, J.T. (2006). Entries in the Leiden Duchenne muscular dystrophy mutation database: an overview of mutation types and paradoxical cases that confirm the reading-frame rule. Muscle Nerve *34*, 135–144. https://doi.org/10.1002/mus.20586.
5. Den Dunnen, J.T., Grootscholten, P.M., Bakker, E., Blonden, L.A., Ginjaar, H.B., Wapenaar, M.C., van Paassen, H.M., van Broeckhoven, C., Pearson, P.L., and van Ommen, G.J. (1989). Topography of the Duchenne muscular dystrophy (DMD) gene: FIGE and cDNA analysis of 194 cases reveals 115 deletions and 13 duplications. Am. J. Hum. Genet. *45*, 835–847.
6. Nobile, C., Marchi, J., Nigro, V., Roberts, R.G., and Danieli, G.A. (1997). Exon-intron organization of the human dystrophin gene. Genomics *45*, 421–424. https://doi.org/10.1006/geno.1997.4911.
7. Campbell, K.P., and Kahl, S.D. (1989). Association of dystrophin and an integral membrane glycoprotein. Nature *338*, 259–262. https://doi.org/10.1038/338259a0.
8. Wilson, D.G.S., Tinker, A., and Iskratsch, T. (2022). The role of the dystrophin glycoprotein complex in muscle cell mechanotransduction. Commun. Biol. *5*, 1022. https://doi.org/10.1038/s42003-022-03980-y.
9. Ervasti, J.M., and Campbell, K.P. (1993). A role for the dystrophin-glycoprotein complex as a transmembrane linker between laminin and actin. J. Cell Biol. *122*, 809–823. https://doi.org/10.1083/jcb.122.4.809.
10. Marrone, A.K., and Shcherbata, H.R. (2011). Dystrophin Orchestrates the Epigenetic Profile of Muscle Cells Via miRNAs. Front. Genet. *2*, 64. https://doi.org/10.3389/fgene.2011.00064.
11. Kodippili, K., and Rudnicki, M.A. (2023). Satellite cell contribution to disease pathology in Duchenne muscular dystrophy. Front. Physiol. *14*, 1180980. https://doi.org/10.3389/fphys.2023.1180980.
12. Mozzetta, C., Sartorelli, V., Steinkuhler, C., and Puri, P.L. (2024). HDAC inhibitors as pharmacological treatment for Duchenne muscular dystrophy: a discovery journey from bench to patients. Trends Mol. Med. *30*, 278–294. https://doi.org/10.1016/j.molmed.2024.01.007.
13. Ahn, A.H., and Kunkel, L.M. (1993). The structural and functional diversity of dystrophin. Nat. Genet. *3*, 283–291. https://doi.org/10.1038/ng0493-283.
14. Davies, K.E., and Nowak, K.J. (2006). Molecular mechanisms of muscular dystrophies: old and new players. Nat. Rev. Mol. Cell Biol. *7*, 762–773. https://doi.org/10.1038/nrm2024.
15. Feron, M., Guevel, L., Rouger, K., Dubreil, L., Arnaud, M.-C., Ledevin, M., Megeney, L.A., Cherel, Y., and Sakanyan, V. (2009). PTEN Contributes to Profound PI3K/Akt Signaling Pathway Deregulation in Dystrophin-Deficient Dog Muscle. Am. J. Pathol. *174*, 1459–1470. https://doi.org/10.2353/ajpath.2009.080460.
16. Wallace, G.Q., and McNally, E.M. (2009). Mechanisms of muscle degeneration, regeneration, and repair in the muscular dystrophies. Annu. Rev. Physiol. *71*, 37–57. https://doi.org/10.1146/annurev.physiol.010908.163216.
17. Fortunato, F., Rossi, R., Falzarano, M.S., and Ferlini, A. (2021). Innovative Therapeutic Approaches for Duchenne Muscular Dystrophy. J. Clin. Med. *10*, 820. https://doi.org/10.3390/jcm10040820.
18. Godfrey, C., Desviat, L.R., Smedsrød, B., Piétri-Rouxel, F., Denti, M.A., Disterer, P., Lorain, S., Nogales-Gadea, G., Sardone, V., Anwar, R., et al. (2017). Delivery is key: lessons learnt from developing splice-switching antisense therapies. EMBO Mol. Med. *9*, 545–557. https://doi.org/10.15252/emmm.201607199.

19. Hammond, S.M., Aartsma-Rus, A., Alves, S., Borgos, S.E., Buijsen, R.A.M., Collin, R.W.J., Covello, G., Denti, M.A., Desviat, L.R., Echevarría, L., et al. (2021). Delivery of oligonucleotide-based therapeutics: challenges and opportunities. EMBO Mol. Med. *13*, e13243. https://doi.org/10.15252/emmm.202013243.

20. Blitek, M., Phongsavanh, X., and Goyenvalle, A. (2024). The bench to bedside journey of tricyclo-DNA antisense oligonucleotides for the treatment of Duchenne muscular dystrophy. RSC Med. Chem. *15*, 3017–3025. https://doi.org/10.1039/d4md00394b.

21. Roberts, T.C., Langer, R., and Wood, M.J.A. (2020). Advances in oligonucleotide drug delivery. Nat. Rev. Drug Discov. *19*, 673–694. https://doi.org/10.1038/s41573-020-0075-7.

22. Ferlini, A., Goyenvalle, A., and Muntoni, F. (2021). RNA-targeted drugs for neuromuscular diseases. Science *371*, 29–31. https://doi.org/10.1126/science.aba4515.

23. Relizani, K., Echevarría, L., Zarrouki, F., Gastaldi, C., Dambrune, C., Aupy, P., Haeberli, A., Komisarski, M., Tensorer, T., Larcher, T., et al. (2022). Palmitic acid conjugation enhances potency of tricyclo-DNA splice switching oligonucleotides. Nucleic Acids Res. *50*, 17–34. https://doi.org/10.1093/nar/gkab1199.

24. Chamberlain, J.S., Pearlman, J.A., Muzny, D.M., Gibbs, R.A., Ranier, J.E., Caskey, C.T., and Reeves, A.A. (1988). Expression of the murine Duchenne muscular dystrophy gene in muscle and brain. Science *239*, 1416–1418. https://doi.org/10.1126/science.3347839.

25. Haslett, J.N., Sanoudou, D., Kho, A.T., Bennett, R.R., Greenberg, S.A., Kohane, I.S., Beggs, A.H., and Kunkel, L.M. (2002). Gene expression comparison of biopsies from Duchenne muscular dystrophy (DMD) and normal skeletal muscle. Proc. Natl. Acad. Sci. USA *99*, 15000–15005. https://doi.org/10.1073/pnas.192571199.

26. Spitali, P., van den Bergen, J.C., Verhaart, I.E.C., Wokke, B., Janson, A.A.M., van den Eijnde, R., den Dunnen, J.T., Laros, J.F.J., Verschuuren, J.J.G.M., 't Hoen, P.A.C., and Aartsma-Rus, A. (2013). DMD transcript imbalance determines dystrophin levels. FASEB J. *27*, 4909–4916. https://doi.org/10.1096/fj.13-232025.

27. García-Rodríguez, R., Hiller, M., Jiménez-Gracia, L., van der Pal, Z., Balog, J., Adamzek, K., Aartsma-Rus, A., and Spitali, P. (2020). Premature termination codons in the DMD gene cause reduced local mRNA synthesis. Proc. Natl. Acad. Sci. USA *117*, 16456–16464. https://doi.org/10.1073/pnas.1910456117.

28. Tennyson, C.N., Shi, Q., and Worton, R.G. (1996). Stability of the human dystrophin transcript in muscle. Nucleic Acids Res. *24*, 3059–3064. https://doi.org/10.1093/nar/24.15.3059.

29. Gehring, N.H., Kunz, J.B., Neu-Yilik, G., Breit, S., Viegas, M.H., Hentze, M.W., and Kulozik, A.E. (2005). Exon-junction complex components specify distinct routes of nonsense-mediated mRNA decay with differential cofactor requirements. Mol. Cell *20*, 65–75. https://doi.org/10.1016/j.molcel.2005.08.012.

30. Lamb, Y.N. (2024). Givinostat: First Approval. Drugs *84*, 849–856. https://doi.org/10.1007/s40265-024-02052-1.

31. Consalvi, S., Mozzetta, C., Bettica, P., Germani, M., Fiorentini, F., Del Bene, F., Rocchetti, M., Leoni, F., Monzani, V., Mascagni, P., et al. (2013). Preclinical studies in the mdx mouse model of duchenne muscular dystrophy with the histone deacetylase inhibitor givinostat. Mol. Med. *19*, 79–87. https://doi.org/10.2119/molmed.2013.00011.

32. Licandro, S.A., Crippa, L., Pomarico, R., Perego, R., Fossati, G., Leoni, F., and Steinkühler, C. (2021). The pan HDAC inhibitor Givinostat improves muscle function and histological parameters in two Duchenne muscular dystrophy murine models expressing different haplotypes of the LTBP4 gene. Skelet. Muscle *11*, 19. https://doi.org/10.1186/s13395-021-00273-6.

33. Bizot, F., Goossens, R., Tensorer, T., Dmitriev, S., Garcia, L., Aartsma-Rus, A., Spitali, P., and Goyenvalle, A. (2022). Histone deacetylase inhibitors improve antisense-mediated exon-skipping efficacy in mdx mice. Mol. Ther. Nucleic Acids *30*, 606–620. https://doi.org/10.1016/j.omtn.2022.11.017.

34. Phongsavanh, M., Bizot, F., Saoudi, A., Gastaldi, C., Le Coz, O., Tensorer, T., Brisebard, E., Garcia, L., and Goyenvalle, A. (2025). Valproic Acid Improves Antisense-Mediated Exon-Skipping Efficacy in mdx Mice. Int. J. Mol. Sci. *26*, 2583. https://doi.org/10.3390/ijms26062583.

35. Araki, E., Nakamura, K., Nakao, K., Kameya, S., Kobayashi, O., Nonaka, I., Kobayashi, T., and Katsuki, M. (1997). Targeted disruption of exon 52 in the mouse dystrophin gene induced muscle degeneration similar to that observed in Duchenne muscular dystrophy. Biochem. Biophys. Res. Commun. *238*, 492–497. https://doi.org/10.1006/bbrc.1997.7328.

36. Giovarelli, M., Zecchini, S., Catarinella, G., Moscheni, C., Sartori, P., Barbieri, C., Roux-Biejat, P., Napoli, A., Vantaggiato, C., Cervia, D., et al. (2021). Givinostat as metabolic enhancer reverting mitochondrial biogenesis deficit in Duchenne Muscular Dystrophy. Pharmacol. Res. *170*, 105751. https://doi.org/10.1016/j.phrs.2021.105751.

37. Falcucci, L., Dooley, C.M., Adamoski, D., Juan, T., Martinez, J., Georgieva, A.M., Mamchaoui, K., Cirzi, C., and Stainier, D.Y.R. (2025). Transcriptional adaptation upregulates utrophin in Duchenne muscular dystrophy. Nature *639*, 493–502. https://doi.org/10.1038/s41586-024-08539-x.

38. Rossi, R., Aartsma-Rus, A., Muntoni, F., Goyenvalle, A., and Spitali, P. (2026). The complexity of dystrophin transcription and processing: implications of transcript imbalance on dystrophin gene targeting strategies. Neuromuscul. Disord. *59*, 106306. https://doi.org/10.1016/j.nmd.2025.106306.

39. Aupy, P., Zarrouki, F., Sandro, Q., Gastaldi, C., Buclez, P.-O., Mamchaoui, K., Garcia, L., Vaillend, C., and Goyenvalle, A. (2020). Long-Term Efficacy of AAV9-U7snRNA-Mediated Exon 51 Skipping in mdx52 Mice. Mol. Ther. Methods Clin. Dev. *17*, 1037–1047. https://doi.org/10.1016/j.omtm.2020.04.025.

40. Saoudi, A., Barberat, S., le Coz, O., Vacca, O., Doisy Caquant, M., Tensorer, T., Sliwinski, E., Garcia, L., Muntoni, F., Vaillend, C., and Goyenvalle, A. (2023). Partial restoration of brain dystrophin by tricyclo-DNA antisense oligonucleotides alleviates emotional deficits in mdx52 mice. Mol. Ther. Nucleic Acids *32*, 173–188. https://doi.org/10.1016/j.omtn.2023.03.009.

41. Doisy, M., Vacca, O., Fergus, C., Gileadi, T., Verhaeg, M., Saoudi, A., Tensorer, T., Garcia, L., Kelly, V.P., Montanaro, F., et al. (2023). Networking to Optimize Dmd exon 53 Skipping in the Brain of mdx52 Mouse Model. Biomedicines *11*, 3243. https://doi.org/10.3390/biomedicines11123243.

42. Clemens, P.R., Rao, V.K., Connolly, A.M., Harper, A.D., Mah, J.K., Smith, E.C., McDonald, C.M., Zaidman, C.M., Morgenroth, L.P., Osaki, H., et al. (2020). Safety, Tolerability, and Efficacy of Viltolarsen in Boys With Duchenne Muscular Dystrophy Amenable to Exon 53 Skipping. JAMA Neurol. *77*, 982. https://doi.org/10.1001/jamaneurol.2020.1264.

43. Clemens, P.R., Rao, V.K., Connolly, A.M., Harper, A.D., Mah, J.K., McDonald, C.M., Smith, E.C., Zaidman, C.M., Nakagawa, T., CINRG DNHS Investigators, and Hoffman, E.P. (2023). Efficacy and Safety of Viltolarsen in Boys With Duchenne Muscular Dystrophy: Results From the Phase 2, Open-Label, 4-Year Extension Study. J. Neuromuscul. Dis. *10*, 439–447. https://doi.org/10.3233/JND-221656.

44. Iff, J., Desguerre, I., Liu, Y., Sarkozy, F., Tuttle, E., Muntoni, F., McDonald, C.M., Nougues, M.-C., Amthor, H., Zhong, Y., and Wahbi, K. (2025). Association between exon-skipping therapy with eteplirsen and cardiac outcomes in Duchenne muscular dystrophy. J. Neuromuscul. Dis. *13*, 179–189. https://doi.org/10.1177/22143602251366721.

45. Babuin, L., and Jaffe, A.S. (2005). Troponin: the biomarker of choice for the detection of cardiac injury. CMAJ *173*, 1191–1202. https://doi.org/10.1503/cmaj.050141.

46. Donovan, J., Silverman, J.A., Barthel, B., DuVall, M., Madden, M., MacDougall, J., Kilburn, N.R., Bronson, A., Evanchik, M., Gordon, G., et al. (2025). A Phase 1, Double-Blind, Placebo-Controlled Trial of Sevasemten (EDG-5506), a Selective Modulator of Fast Skeletal Muscle Contraction, in Healthy Volunteers and Adults With Becker Muscular Dystrophy. Muscle Nerve *72*, 399–407. https://doi.org/10.1002/mus.28444.

47. Bettica, P., Petrini, S., D'Oria, V., D'Amico, A., Catteruccia, M., Pane, M., Sivo, S., Magri, F., Brajkovic, S., Messina, S., et al. (2016). Histological effects of givinostat in boys with Duchenne muscular dystrophy. Neuromuscul. Disord. *26*, 643–649. https://doi.org/10.1016/j.nmd.2016.07.002.

48. Echigoya, Y., Lim, K.R.Q., Trieu, N., Bao, B., Miskew Nichols, B., Vila, M.C., Novak, J.S., Hara, Y., Lee, J., Touznik, A., et al. (2017). Quantitative Antisense Screening and Optimization for Exon 51 Skipping in Duchenne Muscular Dystrophy. Mol. Ther. *25*, 2561–2572. https://doi.org/10.1016/j.ymthe.2017.07.014.

49. Mamchaoui, K., Trollet, C., Bigot, A., Negroni, E., Chaouch, S., Wolff, A., Kandalla, P.K., Marie, S., Di Santo, J., St Guily, J.L., et al. (2011). Immortalized pathological human myoblasts: towards a universal tool for the study of neuromuscular disorders. Skelet. Muscle *1*, 34. https://doi.org/10.1186/2044-5040-1-34.

50. Rouillon, J., Poupiot, J., Zocevic, A., Amor, F., Léger, T., Garcia, C., Camadro, J.-M., Wong, B., Pinilla, R., Cosette, J., et al. (2015). Serum proteomic profiling reveals fragments of MYOM3 as potential biomarkers for monitoring the outcome of therapeutic interventions in muscular dystrophies. Hum. Mol. Genet. *24*, 4916–4932. https://doi.org/10.1093/hmg/ddv214.

51. Relizani, K., Griffith, G., Echevarría, L., Zarrouki, F., Facchinetti, P., Vaillend, C., Leumann, C., Garcia, L., and Goyenvalle, A. (2017). Efficacy and Safety Profile of Tricyclo-DNA Antisense Oligonucleotides in Duchenne Muscular Dystrophy Mouse Model. Mol. Ther. Nucleic Acids *8*, 144–157. https://doi.org/10.1016/j.omtn.2017.06.013.

OMTN, Volume *37*

Supplemental information

Givinostat enhances antisense oligonucleotide efficacy in the *mdx52* model of Duchenne muscular dystrophy

Xaysongkhame Phongsavanh, Cecile Gastaldi, Astrid Mottais, Mathilde Doisy, Olivier Le Coz, Sandra de Haan, Pietro Spitali, Luis Garcia, and Aurélie Goyenvalle

Supporting methods

Cell viability assay following HDACi treatment during early differentiation

To evaluate the cytotoxic effects of histone deacetylase inhibitors (HDACi) during early myogenic differentiation, cell viability was assessed using the MTT Cell Proliferation Assay Kit (VWR, OZBIMT01000) after 48 hours of treatment. Human and murine myoblasts were seeded in 6-well plates at a density of 300,000 cells per well in their respective proliferation media (see *Cell Culture, Differentiation, HDACi Treatment, and Oligonucleotide Transfection* for detailed compositions). Cells were maintained under standard incubation conditions (human cells: 37°C, 5% CO_2; murine cells: 33°C, 10% CO_2). Twenty-four hours after seeding, cells were treated with HDAC inhibitors at various concentrations: Givinostat (Givi) and trichostatin A (TSA) at 10 nM, 50 nM, 150 nM, and 300 nM; valproic acid (VPA) at 0.5 mM, 1 mM, 3 mM, and 10 mM. Differentiation was induced simultaneously by replacing the proliferation medium with the appropriate differentiation medium (see *Cell Culture* section for formulations), and cells were incubated under differentiation conditions (37°C, 5% CO_2) for 48 hours. Following treatment, the MTT assay was performed according to the manufacturer's instructions. Cells were incubated with MTT reagent for the recommended duration (typically 2–4 hours), after which the resulting formazan crystals were solubilized using the provided solubilization buffer. Absorbance was measured at 570 nm using a microplate reader. Cell viability was expressed as a percentage relative to untreated controls. All conditions were tested in triplicate (n = 3) and appropriate vehicle controls were included for each HDACi treatment.

ASO quantification by fluorescent hybridization assay

Tissues were homogenized using the Precellys 24 (Bertin Instruments, France) in lysis buffer (100 mmol/l Tris–HCl, pH 8.5, 200 mmol/l NaCl, 5 mmol/l EDTA, 0.2% sodium dodecyl sulfate) containing 2 mg/ml of proteinase K (Invitrogen), at a final tissue concentration of 50 mg per ml of buffer. The homogenates were incubated overnight at 55°C in a hybridization oven. After a 15-minute

centrifugation at 7,000 g (Sorval ST 8R centrifuge, 75005719 rotor), the supernatant was collected for further analysis. ASO quantification was performed using a hybridization assay with a molecular beacon probe, as previously described [39]. The concentration of tcDNA in tissues was determined by comparing the fluorescence signals to a standard curve generated from known amounts of tcDNA dissolved in tissue lysates from PBS-injected animals.

Table S1. Quantification of dystrophin restoration across muscles following 4- and 12-week treatment protocols.

Tables showing the values for quantification of dystrophin restoration for each muscle following the 4-week (n = 4 per group) and 12-week (n = 6 per group) treatment protocols (mean ± SEM, expressed as a percentage of wild-type dystrophin). Fold-change values between treatments are shown for the 12-week protocol. An average fold change of ~1.3-fold is shown and corresponds to the average fold change across all muscles, indicating that the combined Givi + ASO treatment increases dystrophin restoration compared with ASO monotherapy. TA: tibialis anterior, GAS: gastrocnemius, Q: quadriceps, TRI: triceps, DIA: diaphragm, heart.

4-weeks	TA	TRI	DIA	Heart	Average
**ASO**	**0.27** ± 0.07 %	**0.07** ± 0.04 %	**0.00** ± 0.00 %	**2.14** ± 0.8 %	**0.62** ± 0.22
**Givi+ASO**	**1.42**	**0.80**	**0.54**	**3.17**	**1.48**

12-weeks	TA	GAS	Q	TRI	DIA	Heart	Average
ASO	**2.17** ± 0.12 %	**2.7** ± 0.76 %	**3.44** ± 0.42 %	**2.10** ± 0.48 %	**2.25** ± 0.22 %	**5.21** ± 1.59 %	**2.99** ± 0.60
Givi+ASO	**2.09** ± 0.21 %	**4.22** ± 0.89 %	**3.77** ± 0.13 %	**2.91** ± 0.26 %	**3.48** ± 0.60 %	**7.26** ± 1.48 %	**3.86** ± 0.60
Fold change	**0.96**	**1.53**	**1.09**	**1.10**	**1.55**	**1.39**	**1.29**

Table S2. List of genes with their associated primers and TaqMan probes

Targets	Forward (5' →3')	Reverse (5'→3')	Probes
hex4-5 (Hs.PT.58.27519607)	TGCCCTGAACAATGTCAACA	CTGCCAGTGGAGGATTATATTCC	/56-FAM/CAAACCAAG/ZEN/AGTCAGTTTATGATTTCCATCTACGA/3IABkFQ/
hex23-24 (Hs.PT.5821042878)	GAAGAAGCTCTCCTCCCA	CTTCAGAAAAACATCAACTTCAGC	/56-FAM/CTCCGAAAA/ZEN/ATTCAGAATCACATACAAACCCTG/3IABkFQ/
hex49-50 (hDMD49-50 set 3)	GTTTACCGCCTTCCACTCA	TTTGTCTAAAGGGCAGCATTTG	/56-FAM/CTTCCTCTT/ZEN/CACTGGCTGAGTGGC/3IABkFQ/
hex53-54 (Hs.PT.58.24504618)	GTCCCTATACAGTAGATGCAATCC	ATAATCCCGGAGAAGTTTCAGG	/56-FAM/CACTGGCGG/ZEN/AGGTCTTTGGC/3IABkFQ/
hex58-59 (Hs.PT.58.21052090)	CTCTTGAGACTGTACGAATATTTCTG	GAGTGACATTCTGGGCTCTC	/56-FAM/TCTCTAGTC/ZEN/CTTCCAAAGGCTGCTCT/3IABkFQ/
hEx65-66 (Hs.PT.58.1307753)	CTCTGCGTGGATATGTGTCTG	CTGTACTTGTCTTCCAAATGTGC	/56-FAM/TGGCTGCTG/ZEN/AATGTTTATGATACGGGA/3IABkFQ/
hGAPDH (Hs.PT.39a22214836)	ACATCGCTCAGACACCATG	TGTAGTTGAGGTCAATGAAGGG	/56-FAM/AAGGTCGGA /ZEN/GTCAACGGATTTGGTC/3IABkFQ/
mex4-5 (Mm.PT.58.41636025)	ATCCACTATGTCAGTGCTTCC	AGGGATCTACAAGAGTTCATGC	/56-FAM/AACAATGTC /ZEN/AACAAGGCACTGCGG /3IABkFQ/
mex23-24 (Mm.PT.58.434322707)	CAGGCCATTCCTCTTTCAGG	GAAACTTTCCTCCCAGTTGGT	/56-FAM/TCAACTTCA /ZEN/GCCATCCATTTCTGTAAGGT/3IABkFQ/
mex49-50 (Mm.PT.58.6233636)	GATGTGGAAAGGCTTTTGTCG	CTCCCACTCAGACCTCAGAT	/56-FAM/AACCAAGCA /ZEN/CTCAGCCAGTGAAGA/3IABkFQ/
mex53-54 (Mm.PT. 58.41709800)	AGAAGGTCCTCACACAGTAGA	CAGCAGAATAGTCCCGAAGAAG	/56-FAM/CGGCAGATA/ZEN/AGTGTAGACGTGGCA/3IABkFQ/
mex58-59 (Mm.PT.58.43613256)	TTCTGACAGAGCAGCCTTTG	GAGCCGAGTGACATTCTGAG	/56-FAM/AGCCCAGAG /ZEN/AACTGCCTCCTG/3IABkFQ/
mex65-66 (Mm.PT.58.42993407)	GTGTCTCAACTGGCTTCTCAA	CTTTACACAGAAATGATGCCA	/56-FAM/TTATGATAC/ZEN/GGGACGAACAGGGAGGA/3IABkFQ/

mGAPDH (Mm.PT.39a.1)	AATGGTGAAGGTCGGTGTG	GTGGAGTCATACTGGAACATGTA	/56-FAM/TGCAAATGG/ZEN/CAGCCCTGGTG/3IABkFQ/
mex50-51 (MM.PT.58.41685801)	CAAAGCAGCCTGACCGT	TGACAGTTTCCTTAGTAACCACAG	/56-FAM/TGGACTGAG/ZEN/CACTACTGGAGCCT/3IABkFQ/
mexon 50-53	CTTCCAGCCATTGTGTTGAATC	GCACTACTGGAGCCTTTGAA	/56-FAM/ACAGCTGCA/ZEN/GAACAGGAGACAACA/3IABkFQ/
hex49F	AAACTGAAATAGCAGTTCAAGC		
hex53R		TTGCCTCCGGTTCTGAAGG	
mex50F	AGGAAGTTAGAAGATCTGAGG		
mex55R		GGAACTGCTGCAGTAATCTATGA	
CTGF	TTGACAGGCTTGGCGATT	GTTACCAATGACAATACCTTCTGC	
Col1a1	CATTGTGTATGCAGCTGACTTC	CGCAAAGAGTCTACATGTCTAGG	
Mfap4	CAGCTTGTAGTCGCTCCAG	CGTCTTCTGCGACATGACAA	
PGC-1α	TCGCTCAATAGTCTTGTTCTCAA	AGAAGTCCCATACACAACCG	
Myh1	GGACAAACTGCAATCAAAGGTC	CTGGATCTTGCGGAATTTGGG	
Apol9A	GCAGCTACTGTGACTGATGT	GGCAATCCTATCCACACTTGT	
Ddx60	CTCCTTGTTATGATCCGATGTCC	ATGGTACTGTCTACTTGCTTCC	
Isg15	CACAGTGATCAAGCATTTGCG	CCCCCATCATCTTTTATAACCAAC	
Oasl2	GCTTCACAGAGTTACAGAGACA	GCACCTCTTCCATATTTACGTCT	
MyoG	GACCGAACTCCAGTGCATT	CTTGCTCAGCTCCCTCAAC	

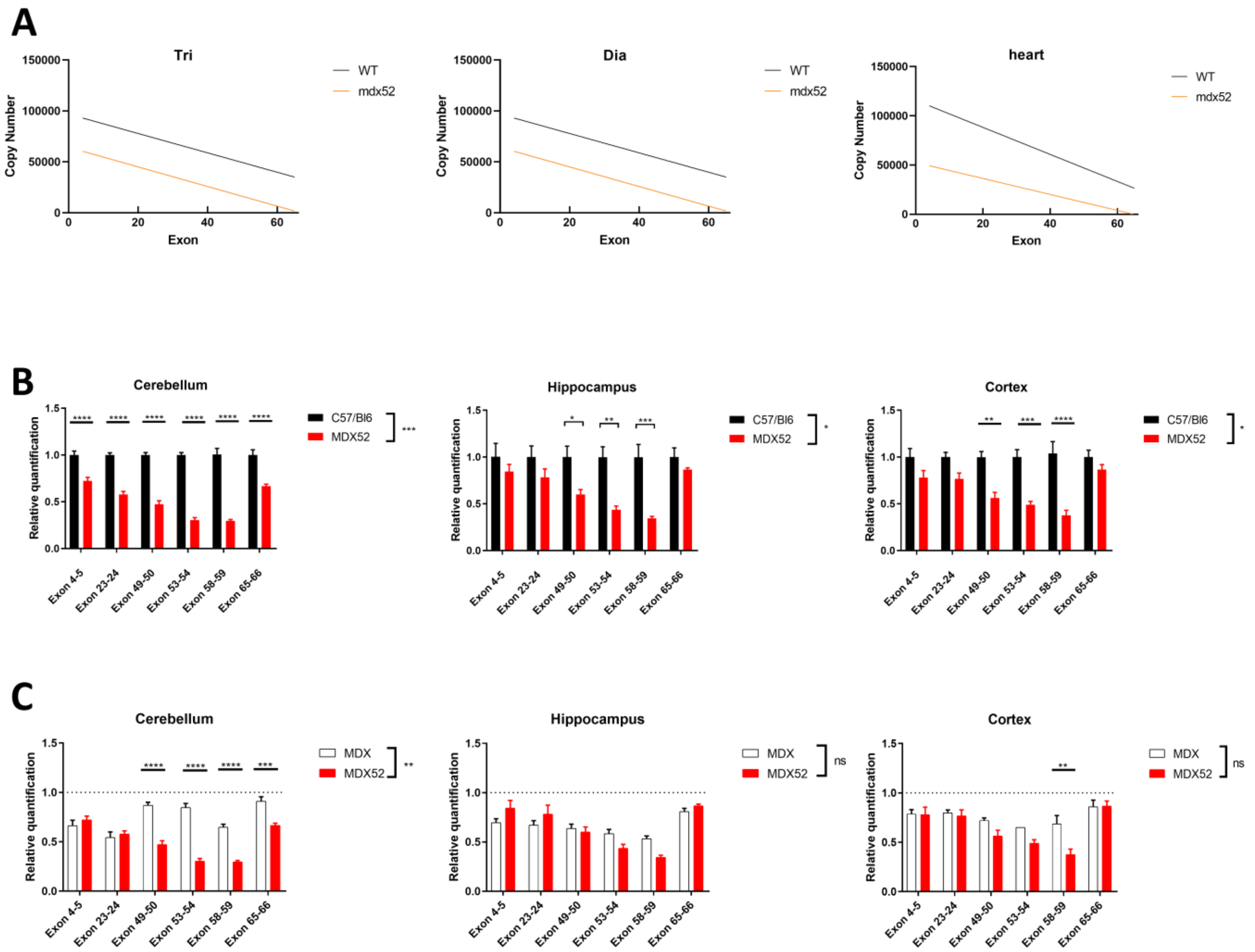

Figure S1. Additional transcriptomic and viability analyses. (A) Comparison of *Dmd* 5′–3′ transcript imbalance between *C57BL/6 WT* and *mdx52* mice. Linear regression analysis of 5′–3′ *Dmd* transcript imbalance was performed across different exon–exon junctions in triceps, diaphragm, and heart tissues from wild-type (*WT*; black) and *mdx52* (orange) mice (n = 3/group). **(B)** In vivo characterization of *Dmd* transcript imbalance in the brain by qPCR. Relative transcript levels across exon–exon junctions of the *Dmd* gene were measured in the cerebellum, hippocampus, and cortex of *mdx52* and *C57BL/6 WT* mice (n = 3/group), normalized to GAPDH and presented relative to WT. Significant imbalances: cerebellum (p = 0.0001), hippocampus (p = 0.0497), cortex (p = 0.0188); two-way ANOVA. **(C)** Comparison of *Dmd* transcript imbalance in *mdx* vs *mdx52* brain tissues. Transcript levels were quantified in cerebellum, hippocampus, and cortex (n = 3/group), normalized to *GAPDH* and WT. A significant difference was found in the cerebellum (p = 0.0083); hippocampus and cortex were not significantly different (p = 0.8773, p = 0.1538); two-way ANOVA.

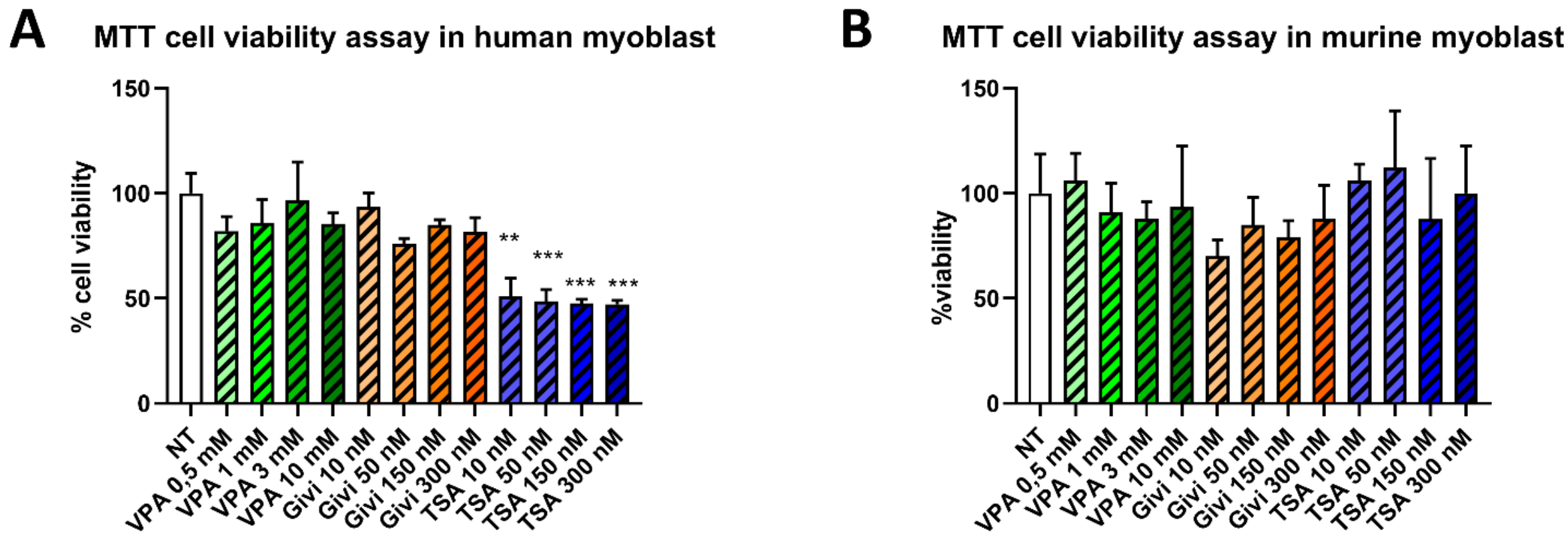

Figure S2. Cell Viability of Human and Murine Myoblasts Following HDACi Treatment. (A) Human and **(B)** murine myoblasts were plated at 300,000 cells/well, treated with 24h of Valproic Acid (VPA), Givinostat (Givi), or Trichostatin A (TSA), and differentiated for 2 days (n = 3/group). Cell viability was assessed using MTT assay. Left: viability in human myoblasts. Right: murine myoblasts. White = PBS; green, orange, blue = VPA, Givi, TSA; hatched = ASO. One-way ANOVA: Significant differences between PBS and human myotubes treated with TSA 10 nM, 50 nM, 150 nM and 300 nM (p = 0.0011, p = 0.0006, p = 0.0005 and p = 0.0004 respectively).

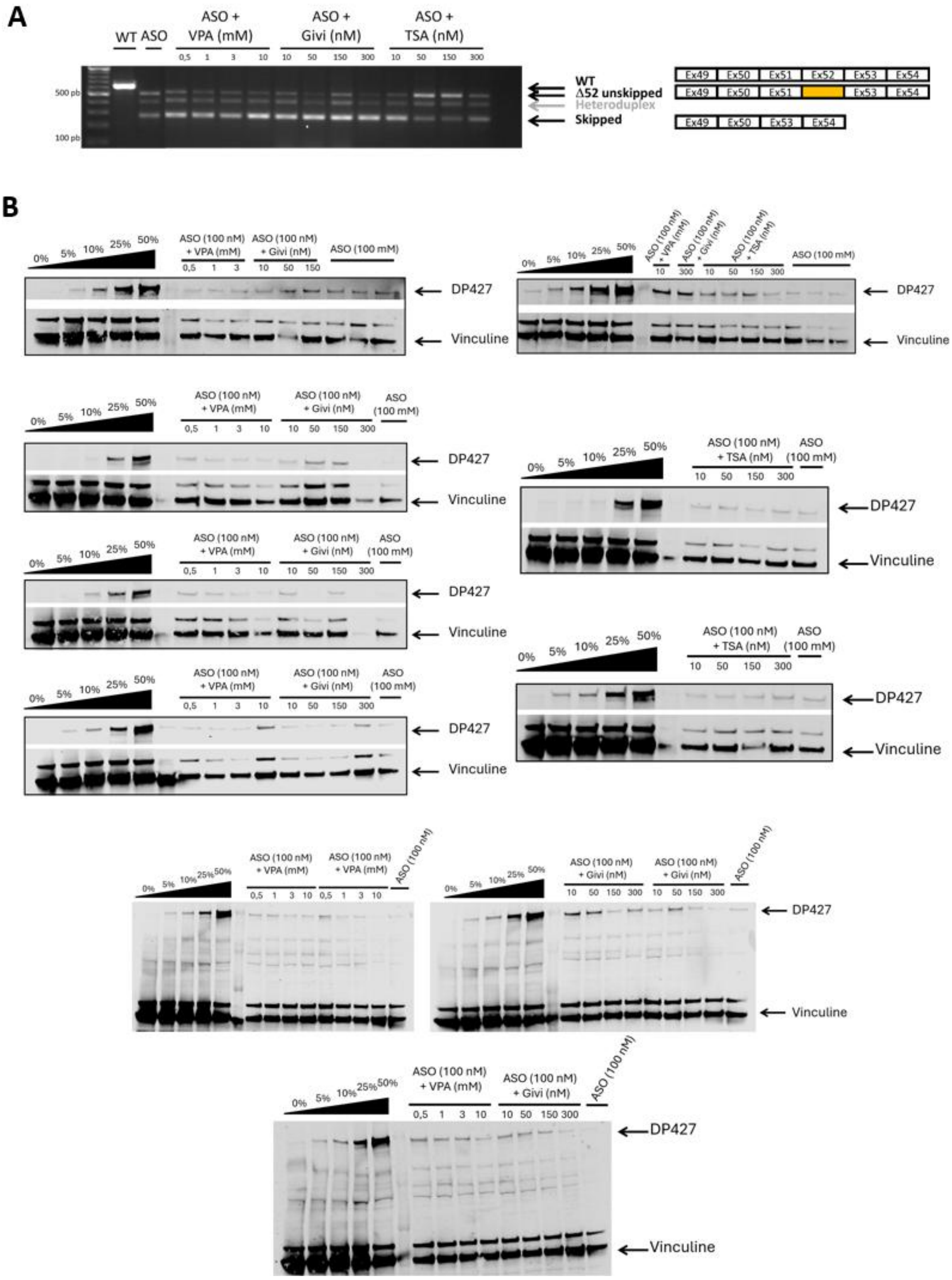

Figure S3. Effect of combined therapy in human cells. **(A)** Example of RT-PCR gel electrophoresis showing exon 51 skipping after treatment with ASO and increasing HDACi concentrations. Amplification spans exons 49 to 54. The untreated wild-type control shows the top band. In treated *KM571Δ52* samples, three bands are visible: unskipped Δ52 (top), a likely heteroduplex (middle) in gray, and skipped (bottom). **(B)** Western blot showing vinculin for normalization and dystrophin (DP427) restoration following ASO treatment alone or in combination with HDAC inhibitors. Three HDACi were tested at multiple doses: VPA (n=7 replicates for each dose 0.5, 1, 3, and 10 mM), Givinostat (n=7 replicates for each dose 10, 50, 150, and 300 nM), and TSA (n=3 for each dose 10, 50, 150, and 300 nM). A standard curve was generated using mixtures of WT and *mdx52*-PBS protein lysates containing 0–50% dystrophin.

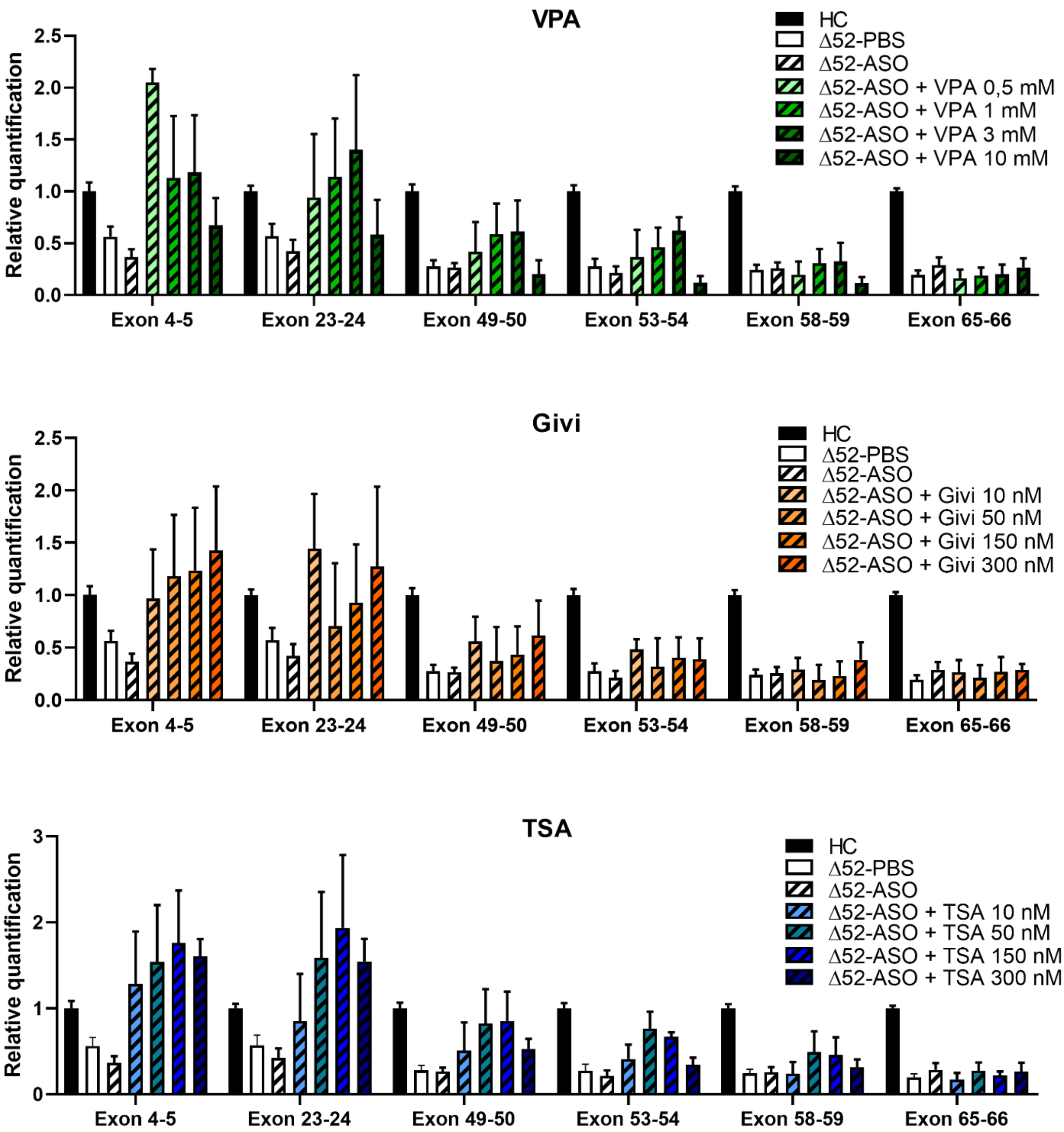

Figure S4. 5′–3′ *Dmd* transcript imbalance following HDACi+ASO treatment in murine cells. ddPCR quantification of *Dmd* transcripts across exon–exon junctions in murine myoblasts (n=3/group) treated with ASO and increasing HDACi concentrations. White = untreated; green, orange, blue = VPA, Givi, TSA; hatched = ASO-treated; black = healthy control (HC). Values normalized to *GAPDH*.

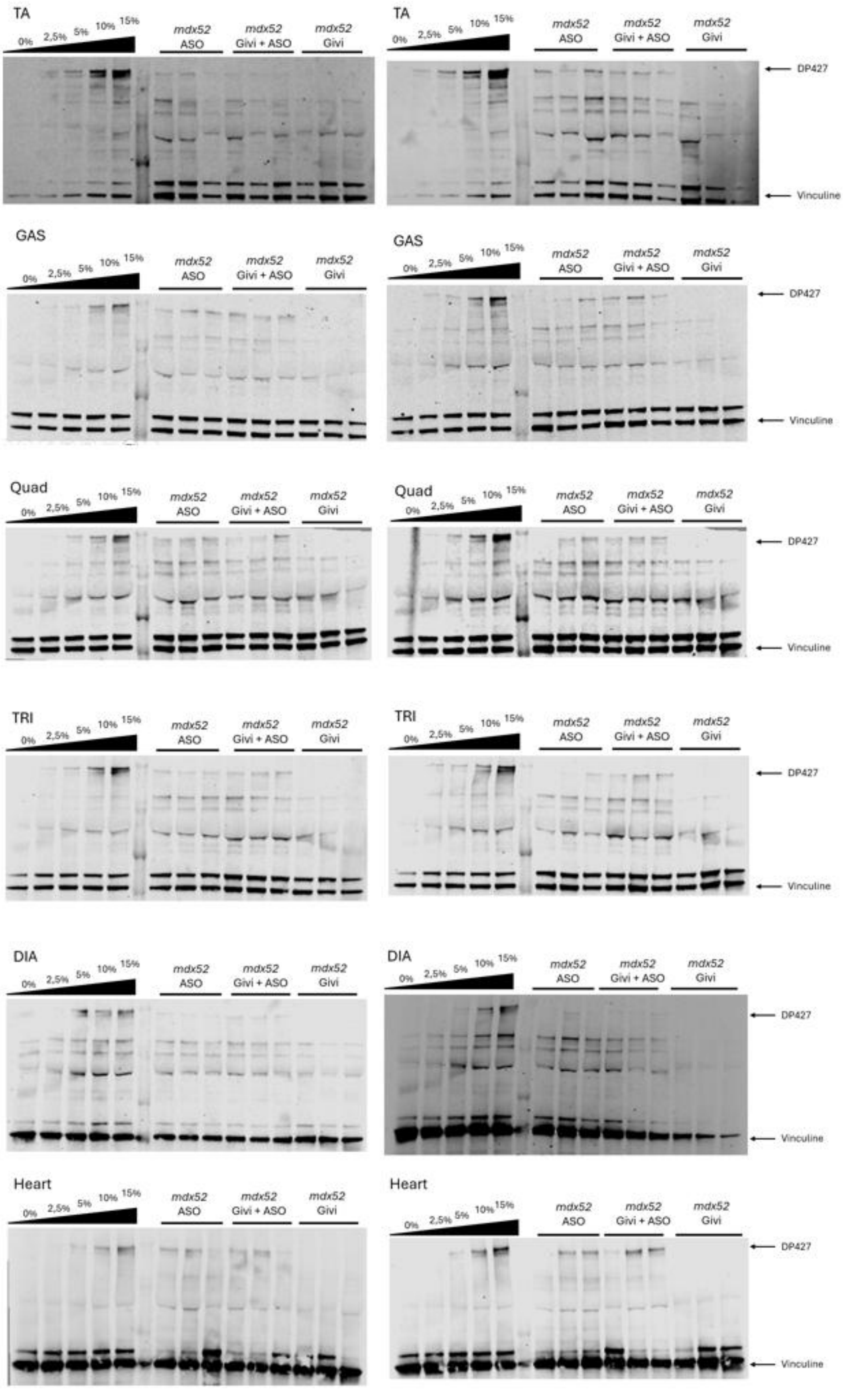

Figure S5. Western blot analysis of dystrophin restoration across muscles after 12-week Givi and/or ASO treatment. All western blot showing vinculin for normalization and dystrophin (DP427) restoration following 12-weeks of Givi or ASO treatment or combined therapy Givi + ASO in different muscles (TA, GAS, Quad, Tri, Dia, Heart). A standard curve was generated using mixtures of WT and *mdx52*-PBS protein lysates containing 0–15% dystrophin. N=6 per group.

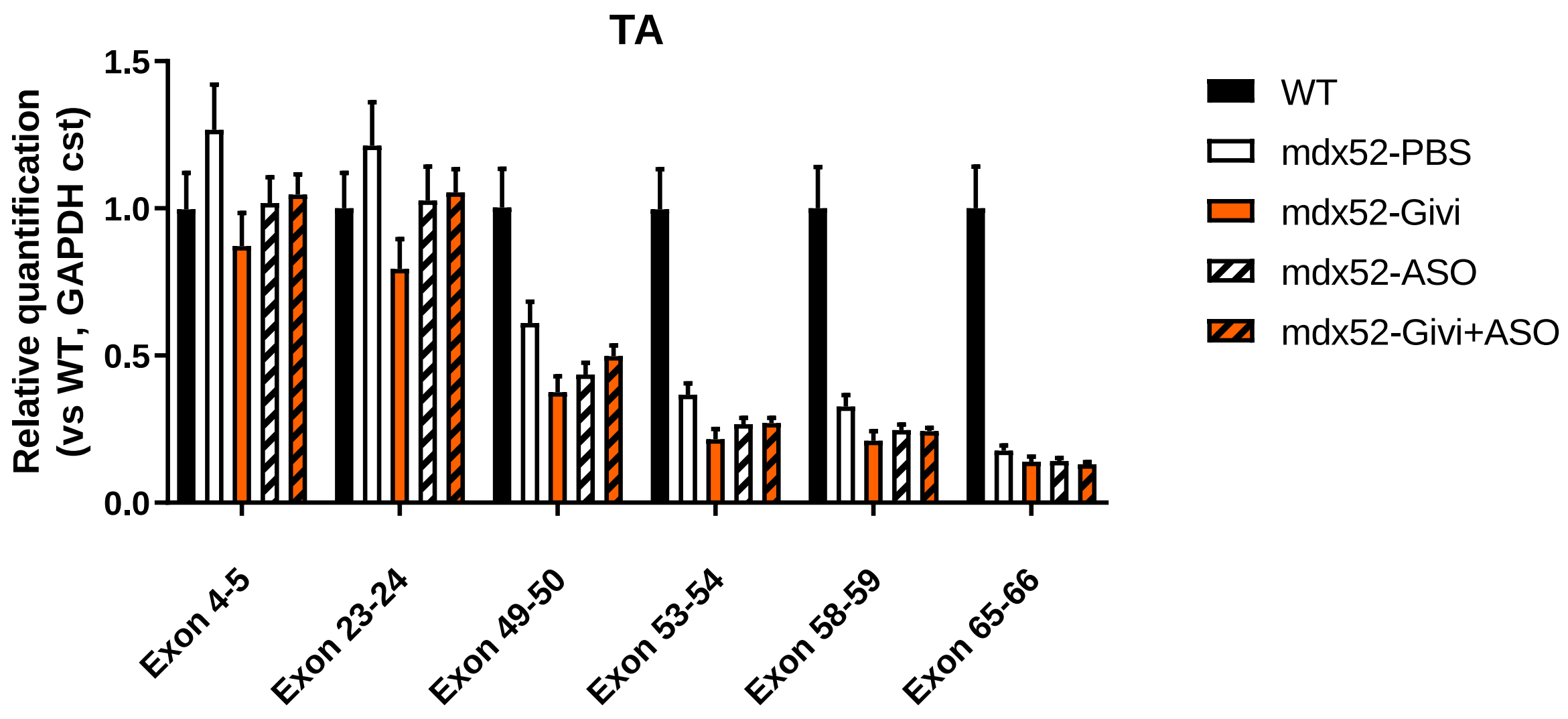

Figure S6. ***Dmd* transcript expression in tibialis anterior after 12-week treatment.**

Dmd expression in tibialis anterior (TA) was analyzed by ddPCR at various exon–exon junctions in *WT* and *mdx52* mice (n = 6/group) treated with saline, Givinostat (Givi), ASO, or Givi+ASO. Data were normalized to GAPDH and relative to WT. No significant differences were detected between HDACi-treated and saline groups (two-way ANOVA).

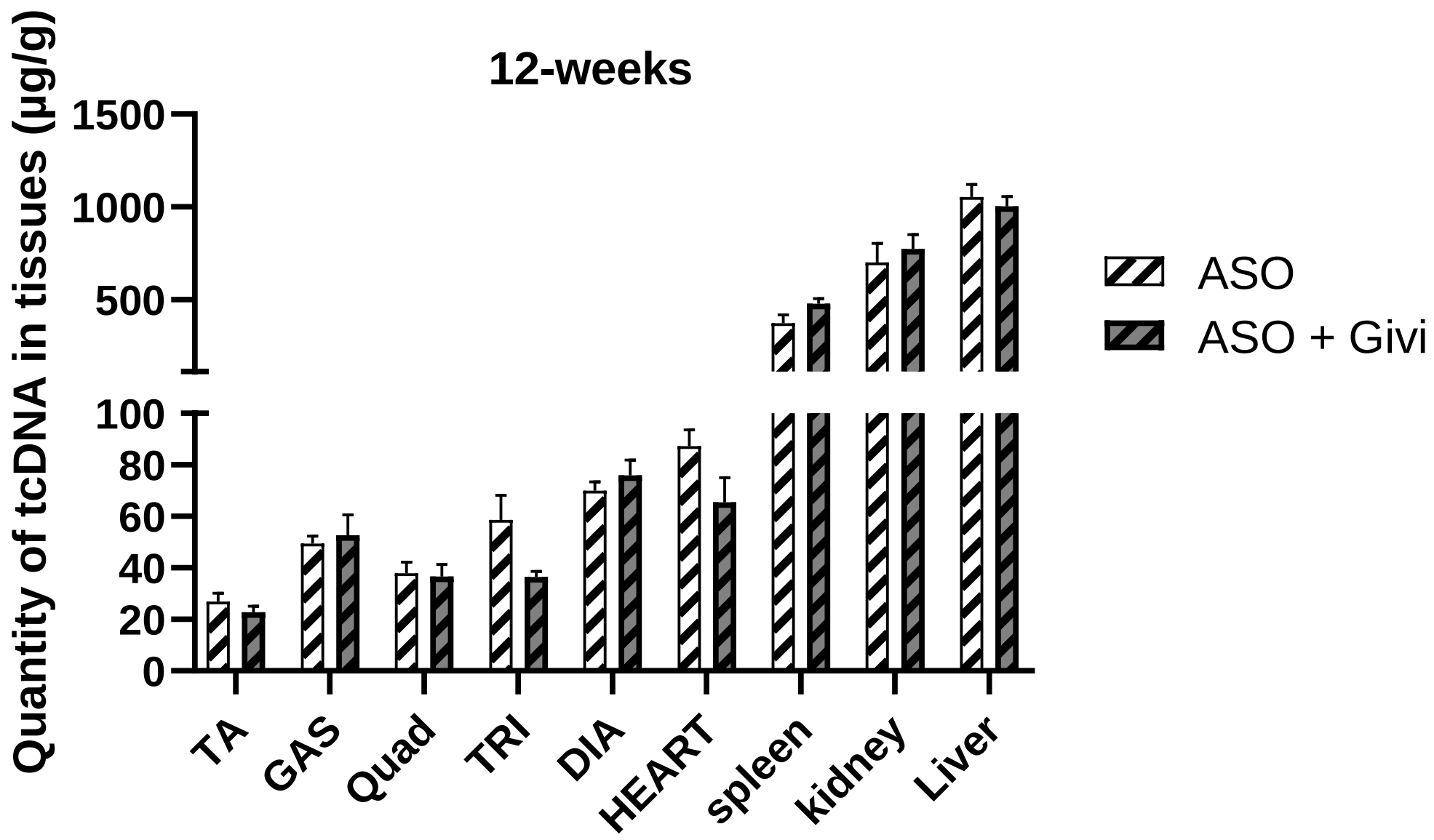

Tissue	TA	GAS	Quad	TRI	Dia	Heart	Spleen	Kidney	Liver
ASO vs Givi+ASO (*p* value)	> 0,9999	> 0,9999	> 0,9999	> 0,9999	> 0,9999	> 0,9999	0,4236	0,8358	0,9846

Figure S7. Quantification of ASO biodistribution in muscle and major organs after 12 weeks. ASO levels were measured in TA, gastrocnemius (GAS), quadriceps (QUAD), triceps (TRI), diaphragm (DIA), heart, and in spleen, liver, and kidney (n = 6/group). No statistical difference was detected between the ASO alone group and the ASO+Givi group using a two-way ANOVA (p = 0.5908). For full transparency, the table below reports the p values from Sidak's multiple comparisons post-hoc test following a two-way ANOVA comparing ASO and ASO+Givi treatments for each tissue after 12 weeks of treatment (n = 6/group).

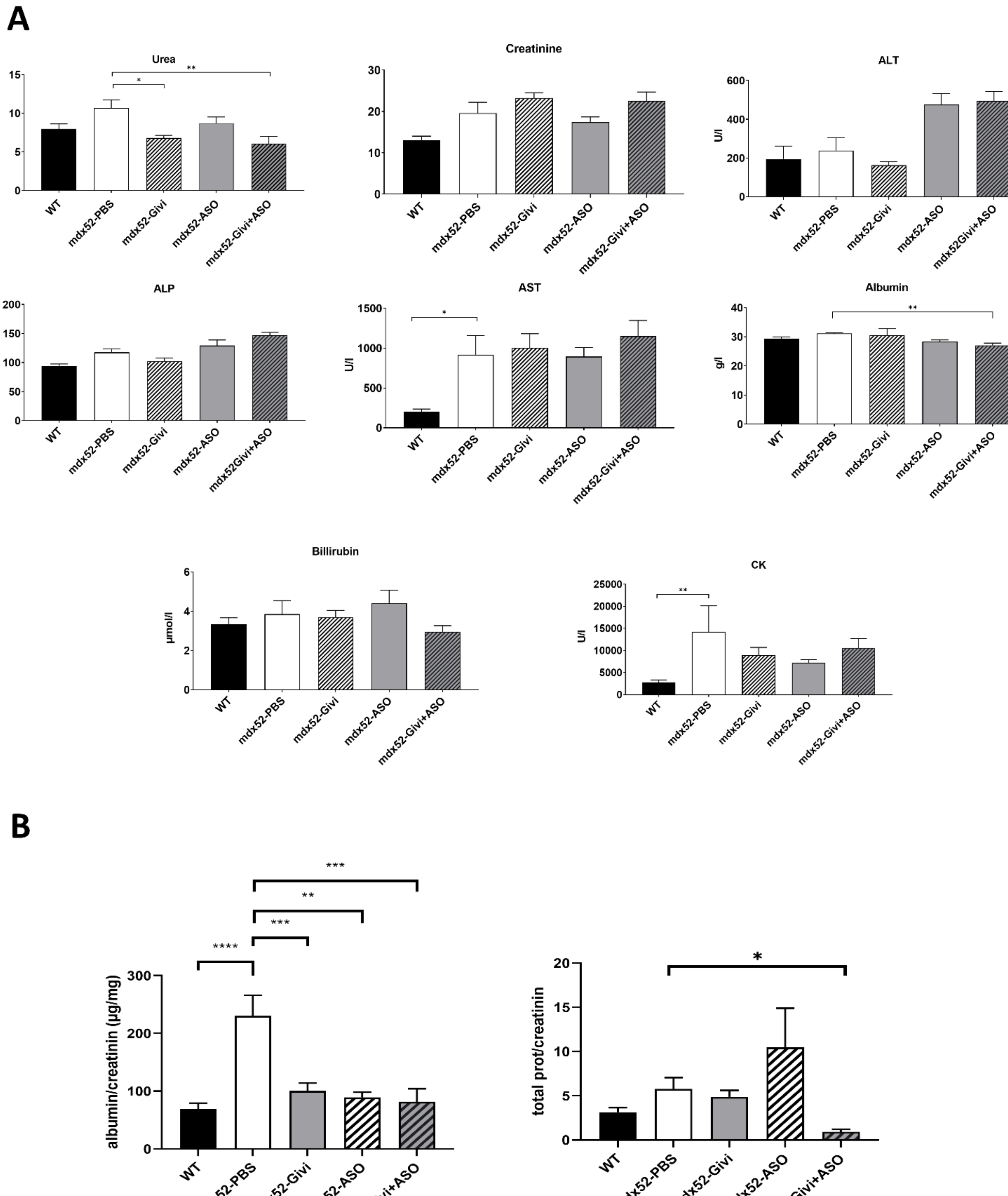

Figure S8. Evaluation of toxicity profile (A) Quantification of general toxicity biomarkers in serum collected two weeks after the last ASO injection. Serum levels of urea, creatinine, ALT, ALP, AST, albumin, bilirubin, and CK were measured in all groups (n = 6 mice per group). Urea levels differed significantly between *mdx52*-PBS and *mdx52*-Givi (p = 0.0291) as well as between *mdx52*-PBS and *mdx52*-Givi+ASO (p = 0.007; Kruskal–Wallis test). AST levels showed a significant difference between WT and *mdx52*-PBS (p = 0.0388; Kruskal–Wallis test). A significant difference in albumin levels was observed between *mdx52*-PBS and *mdx52*-Givi+ASO (p = 0.0012; Kruskal–Wallis test). CK levels were

significantly different between WT and *mdx52*-PBS ($p = 0.0054$; Kruskal–Wallis test). **(B)** Urine analysis. Albumin-to-creatinine ratio (left panel) and total protein-to-creatinine ratio (right panel) were measured in WT and *mdx52* mice treated with saline solution, ASO, Givinostat (Givi), or Givi+ASO (n = 6 per group). For the albumin-to-creatinine ratio, significant differences were observed between WT and *mdx52*-PBS ($p < 0.0001$) and between *mdx52*-PBS and treated groups (Givi: $p = 0.0009$; ASO: $p = 0.0026$; Givi+ASO: $p = 0.0002$; one-way ANOVA). For the total protein-to-creatinine ratio, a significant difference was detected only between *mdx52*-PBS and *mdx52*-Givi+ASO ($p = 0.0365$; one-way ANOVA).
